# A multi-ancestry meta genome-wide association study of migraine among veterans: associations with traumatic brain injury, depression, and post-traumatic stress disorder

**DOI:** 10.1038/s41380-025-03392-4

**Published:** 2025-12-19

**Authors:** Marianna Gasperi, Sara Brin Rosenthal, Adam X. Maihofer, Armand Gerstenberger, Daniel Dochtermann, Hélène Choquet, Alice Pressman, Matthew S. Panizzon, Murray B. Stein, Nathaniel M. Schuster, Saiju Pyarajan, VA Million Veteran Program, Niloofar Afari, Caroline M. Nievergelt

**Affiliations:** 1 VA Puget Sound Health Care System (VAPSHCS), Seattle, WA, USA.; 2 Mental Illness Research Education and Clinical Center (MIRECC), VAPSHCS, Seattle, WA, USA.; 3 Department of Psychiatry and Behavioral Sciences, University of Washington School of Medicine, Seattle, WA, USA.; 4 VA San Diego Healthcare System (VASDHS), San Diego, CA, USA.; 5 VA Center of Excellence for Stress and Mental Health (CESAMH), VASDHS, San Diego, CA, USA.; 6 Department of Psychiatry, University of California San Diego, La Jolla, CA, USA.; 7 Center for Computational Biology & Bioinformatics, Department of Medicine, University of California San Diego, La Jolla, CA, USA.; 8 Center for Data and Computational Sciences (C-DACS), VA Boston Healthcare System (VABHS), Boston, MA, USA.; 9 Division of Research, Kaiser Permanente Northern California (KPNC), Pleasanton, CA, USA.; 10 Kaiser Permanente Bernard J. Tyson School of Medicine, Pasadena, CA, USA.; 11 Center for Behavior Genetics of Aging, University of California San Diego, La Jolla, CA, USA.; 12 Department of Anesthesiology, University of California San Diego, La Jolla, CA, USA.; 13 These authors contributed equally: Niloofar Afari, Caroline M. Nievergelt.

## Abstract

Migraine is a neurovascular disorder that poses a high burden to Veterans, who face a greater risk than sex-matched individuals in the general population. Genetic research on migraine in Veterans and its link to psychiatric comorbidities is limited. We present a meta-analysis of a genome-wide association study (GWAS) of migraine in a predominantly male sample of over 433,000 Veterans, including 87,859 cases, from the Million Veteran Program (MVP), identifying 49 genome-wide significant loci, with 36 novel to this study, of which 7 replicated in an independent prior GWAS (after Bonferroni correction for number of loci tested). Our analyses revealed 283 genes, including some newly associated with migraine: *MAML3, CELF4, IRX1, ASXL1, SPOCD1, CXCL*, and *TLR4*. In silico analyses showed enrichment in brain and uterine tissues, which may reflect broader hormonal or neuroendocrine pathways. Compared to previous migraine GWAS, our results show minimal vascular tissue enrichment, potentially reflecting the sample composition, which was predominantly men and Veterans. Migraine SNP-based heritability was 10% for men and 16% for women, and several sex-specific loci were identified through sex-stratified analyses. Despite high genetic correlations with neuropsychiatric disorders – including post-traumatic stress disorder, depression, and traumatic brain injury – Mendelian randomization analyses found no causal links. Finally, we prioritized potential migraine drug targets, including losmapimod (which reduces production of toxic DUX4 protein) and *TLR4* antagonists.

## INTRODUCTION

Migraine is a neurovascular brain disorder that poses a heavy burden on Veterans and contributes to higher disability and diminished quality of life. Among Veterans, migraine has been shown to have a bidirectional interplay with both physical and psychological stressors associated with military service [1]. Veterans with migraine report worse general health and higher levels of pain and are more likely to have a history of highly comorbid psychiatric disorders, including major depressive disorder (MDD), post-traumatic stress disorder (PTSD), anxiety, as well as neurological health conditions, including a history of traumatic brain injury (TBI) [2, 3]. Epidemiological studies, including our previous work with Veterans, have shown strong associations between migraine and comorbid conditions, including PTSD, MDD, and TBI [4–6]. These comorbidities are associated with greater symptom severity and worse clinical outcomes among those with migraine [7, 8], and Veterans with migraine show a similar level of cognitive symptoms as individuals with history of TBI [5]. Among Veterans, migraine is also a powerful risk factor for suicide, highlighting the potential interplay between migraine, psychiatric disorders, and neurological conditions in this population [9–11]. The psychological and physical stressors associated with military service, including trauma exposure and head injury, may exacerbate migraine symptoms and contribute to a unique presentation of migraine in this group [3]. While Veterans are at a higher risk of migraine than the general population [2, 12, 13], research into the genetic factors contributing to migraine among Veterans and the relationship of migraine to these psychiatric comorbidities remains limited [14].

Migraine is characterized by recurring and, at times, incapacitating episodes of headaches and associated symptoms, including nausea, vomiting, light and sound sensitivity, and other physical, cognitive, and psychological symptoms [15]. Migraine is a major contributor to global disability [16] and exhibits notable sex disparities, with the cumulative lifetime risk estimated at 33% for women and 17% for men [17]. Although migraine incidence rates are similar in male and female children, they increase more significantly in females beginning at puberty, leading to women being three to four times more likely to experience migraine throughout their lifetimes [15]. Variations in biological factors, especially sex hormones including estrogen and progesterone, are believed to contribute to these differences [18]. However, a conclusive explanation for the difference in migraine prevalence between men and women is lacking.

Because research on migraine has primarily centered on women due to their higher prevalence [19], the understanding of migraine in men, including potential sex-specific genetic factors, is underexplored. Although twin studies suggest a similar heritability between men and women [20], it is unclear whether women are at a greater risk for migraine or if men possess genetic protection against migraine. Recent genomic efforts have begun to evaluate genetic differences in migraine with several potential loci specific to women, but these have not been evaluated in a large sample of men [20–23]. Despite recent advances in migraine genomics [23–30], the full genetic landscape, particularly regarding sex-specific differences and comorbid psychiatric disorders, is not fully understood [19, 20, 23, 30]. Research focusing on male Veterans, a population with unique exposures and comorbidities, can offer unprecedented insights.

The Million Veteran Program (MVP) offers a unique opportunity to advance our understanding of migraine and its comorbidities. The MVP is a large biorepository of genetic, electronic health records (EHR), and survey data from U.S. military Veterans. The predominantly male sample, along with the high prevalence of migraine and relevant comorbidities, including MDD, PTSD, TBI, and anxiety disorders, provide a valuable resource for exploring the genetic basis of migraine. In this study, we used MVP’s rich sample of Veterans to explore the genetic nature of migraine. We aimed to 1) elucidate the genetic architecture of migraine in a large, predominantly male Veteran population and 2) explore the relationship between migraine and key Veteran comorbidities—PTSD, MDD, and TBI—to better understand these associations.

In this work, we conducted a multi-ancestry GWAS meta-analysis of migraine in a predominantly male sample of more than 433,000 Veterans from the MVP. This is the first genomic study to focus on migraine in men and to conduct sex-stratified analyses. Migraine phenotype was based on EHR diagnostic codes and self-report survey data. Using data from individuals of European, African, and Hispanic ancestry, including 87,859 cases and 345,151 controls, we report 49 genome-wide significant loci. Of these, 36 loci were novel to this study, with 7 nominally replicating findings from previous migraine GWAS [21]. In the cross-strata analysis, we identified 283 genes associated with migraine, including those from the novel loci such as *MAML3, CELF4, IRX1, ASXL1, SPOCD1, CXCL*, and *TLR4*. In silico characterization revealed significant enrichment of brain tissues and the uterus. While genetic correlations between migraine and neuropsychiatric disorders, which involve both neurological and psychiatric characteristics, including PTSD, MDD, and TBI, were high, Mendelian randomization showed no causal relationships among these conditions. Genomic structural equation modeling revealed a stronger association between migraine and neuropsychiatric disorders than with anxiety/stress, mood, and alcohol use disorders. We identified several potential drug targets, including p38 mitogen-activated protein kinase inhibitors such as losmapimod, and TLR4 antagonists.

## METHODS

### Participants and study design

The current observational study was conducted in the Million Veteran Program (MVP), a national research project to determine how genetic factors, health behaviors, and environment affect Veteran health and illness. The MVP cohort has been previously described in detail [31].

### Race and ethnicity information, and genetic ancestry

Genetic ancestry was defined using Harmonizing Genetic Ancestry and Self-identified Race/Ethnicity (HARE) groups [32]. HARE improves the classification process by combining self-identified race/ethnicity (SIRE) with genetically inferred ancestry (GIA) and enhances classification accuracy by using GIA to refine and, when necessary, impute SIRE. This methodology enhances the reliability of race and ethnicity assignment in genetic research. Fewer than 2% of individuals remain unassigned to a HARE group when there is a discrepancy between participant-identified and genetically inferred ancestry data. In this context, the designation “Hispanic (HIS)” denotes the HARE race and ethnicity groups comprising individuals who identify as Latino or Hispanic. “European (EUR)” refers to individuals who are White but not Hispanic, while “African (AFR)” is used for Veterans who are Black but not Hispanic. The samples representing East Asian and South Asian ancestry were insufficient in size to be incorporated into the analyses.

### Phenotype development and GWAS Cohort

Migraine case and control definitions were derived using EHR ICD-9/10 migraine codes (Supplementary Data 1) and self-reported physician diagnoses of migraine, based on data collected from the MVP Baseline Survey. The MVP Baseline Survey has been described elsewhere [31]. Briefly, on the survey, participants were asked, “Please tell us if you have been diagnosed with…” followed by a list of various health conditions, including migraine headaches. Answers were recorded as “yes” responses. Cases were defined as individuals with *either* one or more inpatient or outpatient ICD codes for migraine or self-reported migraine on the MVP Baseline Survey (Supplementary Data 2). Controls were defined as individuals with no ICD 9/10 codes for migraine *and* those who completed the MVP Baseline Survey but did not endorse a history of migraine diagnosis.

Of the 831,455 individuals eligible, 93,893 had one or more ICD codes for migraine (of these, 26,333 also indicated migraine on the MVP Baseline Survey, 14,037 did not indicate migraine on the MVP Baseline Survey, and 53,523 did not complete the MVP Baseline Survey; Supplementary Data 3). Of the 737,562 individuals with no ICD code for migraine, 24,186 indicated migraine on the MVP Baseline Survey and were added to the cases; 440,255 did not indicate migraine on the MVP Baseline Survey and were classified as controls. Individuals who did not have a migraine ICD code *and* did not complete the MVP Baseline Survey were excluded from the controls to avoid contamination (n = 273,121), resulting in 118,079 potential cases and 440,255 potential controls. This exclusion aimed to avoid potential control misclassification by individuals with migraine who did not complete the self-report survey. Individuals were excluded if they did not have sufficient EHR records (n = 121), were missing sex (n = 131), were not genotyped (n = 116,556), or were missing ancestry (n = 8516). Overall, analyses included 433,010 individuals, 391,622 men (66,083 cases, 325,539 controls), and 41,388 (21,776 cases, 19,612 controls) women.

### Genotyping, imputation, and quality control

Genotyping, imputation, and quality control processes within the MVP have been previously documented and carried out by the MVP project core working group [31]. MVP samples were genotyped with a 723,305 SNP Affymetrix Axiom Biobank array, specially designed for MVP to incorporate variants of interest across ancestries [31]. Minimac4 was used for imputation with data from the TopMed reference panel. The analyses were executed using MVP Release 4 data (GRCh38). The final genotype dataset comprised 96 million genetic variants.

### Computation and statistical analysis

#### GWAS regression and meta-analysis.

GWAS analysis was carried out for migraine in MVP (MVP-migraine) stratified by HARE-derived ancestries (EUR, AFR, HIS) to test the association between migraine and imputed dosages using regenie v3.1.3 [33]. Regenie is a two-step machine-learning method that accounts for relatedness. The initial phase involved an analysis of MVP genotype array data, segmenting SNPs into distinct blocks and utilizing ridge regression to generate predictions. These predictions were subsequently aggregated in a second ridge regression and decomposed by chromosome to allow for leave-one-chromosome-out analyses, which served as covariates for second phase analyses. The second phase utilized MVP Release 4 imputed data for cross-validation and implemented Firth logistic regression alongside saddle point approximation for the binary trait analysis. The analysis models included the first 10 principal components of genotype as covariates. In models that included both men and women together, sex was used as a covariate. SNPs with imputation INFO scores > 0.3, minor allele frequency (MAF) ≥ 0.01, and HWE > 1 × 10^−15^ were reported. A genome-wide significance (GWS) was set for the primary analysis as *p* ≤ 5.0 × 10^−8^. LocusZoom 1.4 was used for regional visualizations of GWS loci with ancestry-matched reference panels from the 1000 Genomes Project (Phase 3) [34].

Three GWAS were conducted for each of the three ancestries (EUR, AFR, HIS): separate sex-stratified analyses for men (referred to as EUR_M, AFR_M, and HIS_M) and women (referred to as EUR_W, AFR_W, and HIS_W) and combined analyses by HARE-derived ancestry, including men and women together (referred to as EUR_C, AFR_C, and HIS_C). In all, we evaluated migraine in a total of nine GWAS strata.

Meta-analysis was conducted for males from three ancestries (n = 391,622; META_M) and females from three ancestries (n = 41,388; META_W), and by meta-analyzing the three combined ancestries (EUR_C, AFR_C, and HIS_C) to include all participants (N = 433,010; META_C), using the METAL software package [35] with default parameters. After filtering for MAF ≥ 0.01 and correcting inconsistent allele labels and strands, 17,466,242 variants remained. Cochran’s Q-test was performed for each SNP to test for heterogeneity of effect.

#### Functional annotation with FUMA.

Genome-wide association study results were annotated using the Functional Mapping and Annotation of Genome-Wide Association Studies (FUMA) platform [36–38]. SNPs were annotated to nearby and relevant genes, positional mapping, and eQTL mapping from all current eQTL databases available on FUMA (excluding old versions of GTEX accessible on the FUMA platform) [36–38]. Default settings were used in all FUMA analyses unless specified. The SNP2Gene module was used to identify independent genomic risk loci and variants in LD with lead SNPs (r2 > 0.6, calculated using ancestry-appropriate 1000 Genomes reference: EUR for European Ancestry, AFR for African ancestry, and AMR for Hispanic Ancestry). FUMA results are reported in hg38. Both positional and eQTL information were used for gene mapping. The functional consequences of SNPs were determined by mapping them to their respective chromosomal positions and reference alleles using annotations from databases including ANNOVAR, Combined Annotation Dependent Depletion (CADD), RegulomeDB (RDB), as well as chromatin states across various tissues and cell types.

#### Fine-mapping.

Fine-mapping was conducted with the SuSiE R package [39]. We used the genome-wide significant loci, with ranges defined by FUMA, from the European summary statistics. LD matrices were computed using the 1000 Genomes European population. Credible sets were defined as all SNPs that had a cumulative PIP > 0.95.

#### Replication.

We used data from seven previous migraine studies (from cohorts with no overlap with MVP) to classify GWS loci identified in this study as known or novel, available through the GWAS catalog [21, 23, 25, 26, 28, 30, 40]. GWS loci identified in our study were classified as *known* if any SNP in the locus around the lead SNP (locus area defined by FUMA’s LD clumping algorithm, 10KB up/downstream) was found to be genome-wide significant in a previous migraine study (*p* < 5 × 10^−8^). Otherwise, the GWS loci in our study were classified as *novel*. To assess the replication status of the novel loci, we evaluated the significance of all SNPs within each GWS locus in summary stats from Choquet et al. [21], which combined GERA and UK Biobank (cases = 28,552, controls = 525,717). Choquet et al., migraine GWAS was used for replication as it was the most recent dataset with publicly available full summary statistics at the time of analysis. Loci that contained at least one SNP that was nominally significant (after Bonferroni correction for 36 loci tested: *p* < 0.05/36) were classified as ‘novel-replicated’. We label these 7 loci ‘novel-replicated’. Otherwise, the locus was classified as *novel-unreplicated*.

#### Gene-based and gene set, and tissue-enrichment analyses with MAGMA.

We conducted gene-based, gene-pathway, and tissue enrichment analyses using the Multi-Marker Analysis of GenoMic Annotation (MAGMA) v1.06 method [41] and the MsigDB v5.2 database [42] on the FUMA platform. SNP-level associations, to identify gene sets and pathways and evaluate tissue-specific enrichment, were aggregated using MAGMA at the gene level. We used default settings, including MAGMA correction for multiple comparisons, for analyses unless otherwise specified.

#### Linkage Disequilibrium Score Regression (LDSC) and SNP-based heritability.

The SNP-based heritability of MIG-MVP was estimated using linkage disequilibrium score (LDSC) regression [43]. The 1000 Genomes reference data phase 3 (1KGPp3) data were used to calculate LD [44]. Population prevalence for liability scale transformation of SNP-based heritability was based on self-report MVP population study migraine prevalence estimates, 8.2% for men and 30.1% for women. Because of the predominantly male (92%) composition of our sample, we prorated the combined lifetime prevalence for migraine [combined sample projected prevalence = (0.082 × 0.92) + (0.301 × 0.08) = 10.0%]. The degree of inflation in the test statistic (GC *λ*) attributable to polygenic signal, as opposed to population stratification, was computed using LD Score Regression (LDSC) with the formula: 1 − (LDSC intercept − 1) / (mean observed χ^2^ − 1) [43].

#### Genetic correlation within ancestries and with traits.

Bivariate LDSC regression was used to assess the genetic correlation (r_g_) of MIG-MVP among EUR_M and EUR_W with neurological and psychiatric traits (TBI, PTSD, ADHD, MDD, anxiety, Tourette’s Syndrome, alcohol dependence, autism spectrum disorder, anorexia nervosa, schizophrenia, obsessive-compulsive disorder, bipolar disorder); and ENIGMA brain imaging brain region variables. Data used for LDSC regression are detailed in Supplementary Data 3. Comparisons were made for EUR samples using the European LD score 1000 Genomes reference [45].

To further investigate the relationship between MVP migraine and additional phenotypes, we utilized publicly available GWAS data from the Complex Trait Genetics Virtual Lab [46]. (https://vl.genoma.io/). Cross-trait LDSC regression was carried out across traits to evaluate genetic correlations with MVP EUR_C migraine. To ensure the robustness of our findings, we limited our analyses to phenotypes exhibiting a SNP-based heritability z-score exceeding 4, resulting in a total of 844 phenotypes for evaluation. Bonferroni adjustment was applied to control for multiple comparisons, setting the threshold for statistical significance at (0.05/844) *p* < 5.92 × 10^−5^.

#### Genomic structural equation modeling (GSEM).

We used genomic structural equation modeling (GSEM; genomicSEM package [47]) to estimate the relationships between brain region volumes and migraine while controlling for intracranial volume (ICV). Genetic covariance and sampling covariance matrices across EUR_C and ENIGMA brain region variables were estimated using LDSC [43], with 1KGPp3 EUR LD reference data. Multiple regression models were fit to each brain region, specifying a linear regression structure where migraine was regressed on both the volume of a specific brain region (e.g., caudate nucleus, amygdala) and ICV (Supplementary Fig. 1). The models specified a covariance between the brain region volume and ICV based on LDSC. Standardized regression coefficients (β) and SE were calculated.

GSEM was used to simultaneously model the relationships among migraine and related neuropsychiatric disorders, including TBI, PTSD, and MDD. To estimate the appropriate number of latent factors in the model, we conducted exploratory factor analysis (EFA) using R factanal function on the odd chromosomes. EFA loadings were used to inform the confirmatory factor analyses (CFA) using the even chromosomes. A separate set of chromosomes was used for EFA and CFA to minimize overfitting [47]. Traits with EFA factor loadings above 0.35 were assigned to CFA factors. For some EFA solutions, traits under 0.35 were assigned using a lenient threshold of 0.20. When a factor had only two traits, loadings were made equal to maintain identification. CFAs were conducted utilizing the weighted least squares (WLS) estimator. We evaluated model fit using Akaike Information Criterion (AIC), Comparative Fit Index (CFI), and Standardized Root Mean Square Residual (SRMR).

#### Univariate and bivariate Gaussian model analysis.

We estimated the genetic overlap between EUR_M and EUR_W and between EUR_C and migraine results from Choquet et al. (2022) [21] using the mixed effects score regression (MiXeR) framework [48]. MiXeR employs a Bayesian methodology to provide posterior probabilities for estimating the number of shared and trait-specific loci. This approach enables an unbiased estimation of genetic overlap, independent of the power of individual GWAS. Polygenicity estimates represent the loci required to account for 90% of the SNP-based heritability. Bivariate MiXeR was used to estimate phenotype-specific and shared polygenicity. We assessed model fit using the Akaike Information Criteria (AIC) values. The analyses were conducted using MiXeR version 1.3.

#### Mendelian randomization.

To evaluate the presence of causal effects between migraine and related traits, we conducted Mendelian randomization (MR). MR analyses were performed using CAUSE, an MR approach using full GWAS summary statistics [49]. CAUSE models account for correlated and uncorrelated horizontal pleiotropy to reduce false positive results. We modeled MVP EUR combined migraine as the exposure variable and traits of interest (psychiatric, brain) with significant genetic correlations as the outcome variables. Converse relationships with migraine being the outcome variable were also evaluated. Default settings were used.

#### Drug-class and drug-set enrichment analyses.

The Open Targets Platform [50](https://platform.opentargets.org) was used to identify potential migraine drug targets based on the genes identified in our results. The Open Targets Platform is an online platform that combines open-source, publicly available data, including the EMBL-EBI ChEMBL (https://www.ebi.ac.uk/chembl/) drug database, alongside tools designed to facilitate evidence-based systematic prioritization of targets for treating diseases.

## RESULTS

### Study cohort and migraine prevalence

We determined migraine prevalence using EHR ICD codes and self-report Baseline Survey data (Supplementary Data 4). MVP Baseline Survey data was unavailable for 280,957 individuals, and Black and Hispanic women were more likely not to have Baseline Survey data (58 and 44%, respectively) than other strata (e.g., White men; 34%). Based on ICD, lifetime migraine prevalence was 10.4% for White, 13.5% for Black, and 13.9% for Hispanic Veterans. Self-reported migraine lifetime prevalence on the Baseline Survey was 9.3% for White, 12.5% for Black, and 13.2% for Hispanic Veterans. Women had a higher prevalence of migraine than men, with the highest ICD prevalence in Hispanic women (36.9%) and the lowest among White men (8.5%). The GWAS sample cases and control definitions were derived from the combination of EHR and the Baseline Survey (see Methods). There were 118,079 individuals with any history of migraine on either or both EHR or Baseline Survey identified as cases, and 440,255 individuals with no history of migraine on both sources were controls (Supplementary Data 2). While individuals with a history of migraine on any one data source could be counted as cases, individuals with incomplete data (missing Baseline Survey) were excluded from the control classification. Consequently, the GWAS sample proportions (Table 1) do not reflect MVP migraine phenotypic lifetime prevalence but are a function of conservative control definitions to minimize contamination (Supplementary Data 4).

The MVP migraine GWAS sample included 433,010 participants, 87,859 cases, and 345,151 controls from three HARE-derived ancestral backgrounds. Table 1 shows the average age of participants at enrollment and migraine prevalence by HARE-derived ancestry/ethnicity categories and sex. European HARE-derived ancestry was the largest ancestry group (EUR, n = 338,743; 59,975 cases, 278,768 controls), followed by African HARE-derived ancestry (AFR, n = 65,178; 19,358 cases, 45,820 controls), and HARE-derived Hispanic (HIS, n = 29,089; 8,526 cases, 20,563 controls). People of East Asian and South Asian ancestry were not analyzed due to the low number of Asian individuals in MVP. The MVP sample was predominantly male, with 90.4% (n = 391,622) men and 9.6% (n = 41,388) women, consistent with the distribution (10% women) in the U.S. VA population [51]. Most participants (88.1%) were more than 50 years old, with 402,234 individuals in this age group. Average age varied across the groups from 65.24 (SD = 12.87) years for EUR to 56.93 (14.94) years for HIS and was lower for migraine cases than controls in all ancestries (*p* < 0.001).

### Genome-wide significant loci

**Fig. 1:**
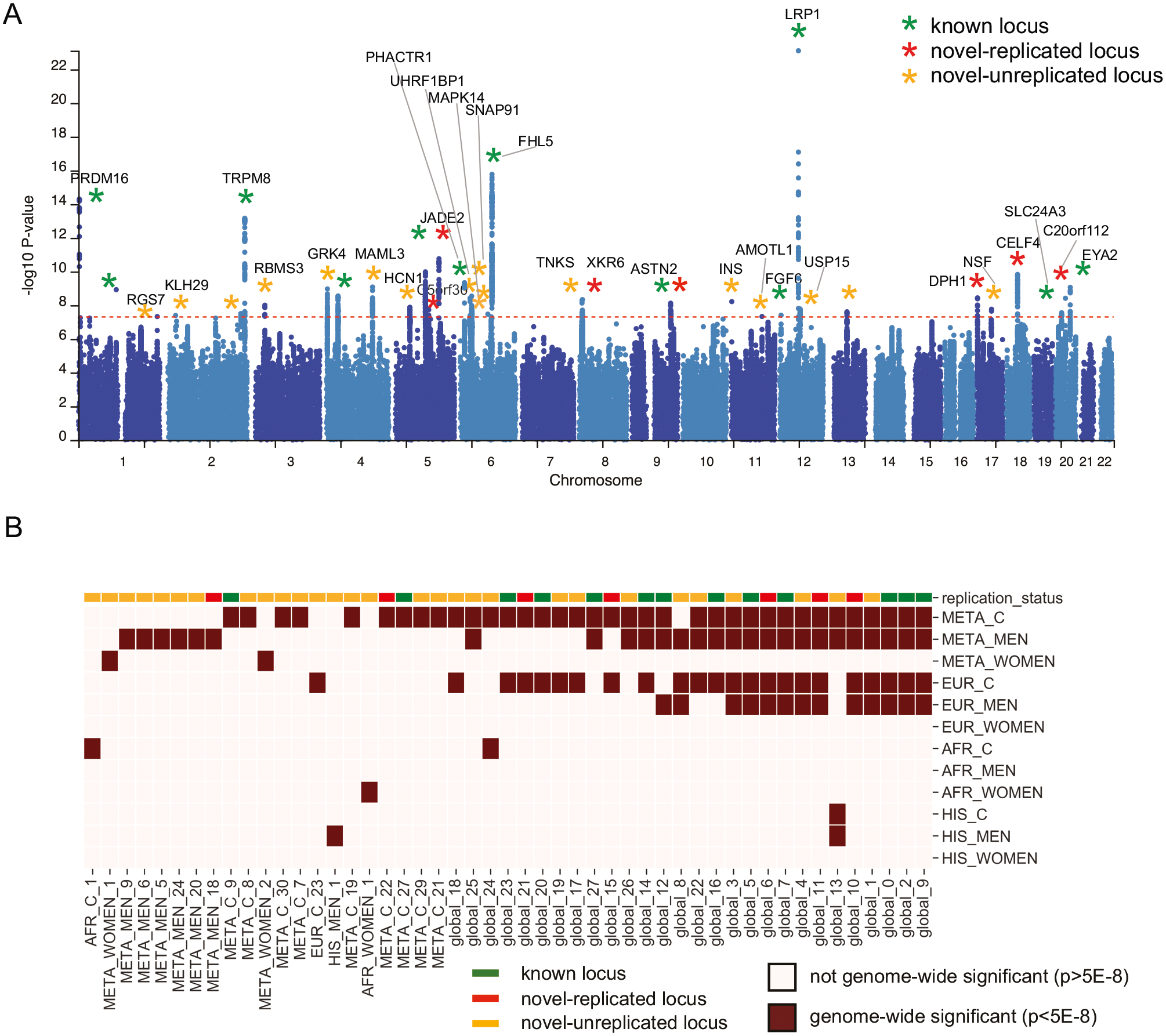
Genome-Wide Significant Loci. **A** Multi-ancestry Meta-Analysis Manhattan plot of GWAS of MVP-Migraine. Green stars indicate loci identified in previous migraine GWAS. Red stars indicate novel loci replicated after Bonferroni adjustment (p < 0.05/36) in the GERA-UKBB cohort. Yellow stars indicate novel loci that did not replicate in the GERA-UKBB cohort. Loci are annotated with selected genes mapped by FUMA. GWS loci were labeled with genes which were positionally mapped to the locus by FUMA (within 10KB of the lead SNP). In cases where more than 1 gene positionally mapped to the lead SNP, the gene with the largest number of positionally mapped GWS SNPs was labeled. GWS loci with no positionally mapped SNPs were not labeled. **B** Summary of genome-wide significant loci across ethnicity and sex. Dark red squares indicate GWS loci. Color bar along the top row indicates if the loci were known, novel-replicated, or novel-unreplicated.

The multi-ancestry meta-analysis (META_C; cases = 87,859, controls = 345,151) across three HARE-defined categories identified 36 genome-wide significant (GWS; *p* < 5 × 10^−8^) loci when accounting for linkage disequilibrium (*r*^*2*^ > 0.1) with 40 lead SNPs corresponding to 188 mapped genes. Quantile-quantile plots showed inflation of test statistic (Supplementary Fig. 3–l; GCl = 1.365), and polygenic effects accounted for 85.6% of this inflation based on the LDSC intercept of 1.0634 (0.009). The META_C findings are summarized in the Manhattan plot in Fig. 1A and Supplementary Data 5, which also reports all loci across all other strata. In total, we identified 23 GWS loci in the EUR strata (EUR_C), one in the HIS strata (HIS_C), and two in the AFR (AFR_C) strata. A meta-analysis of men (META_M; 66,083 cases, 325,539 controls) and women (META_W; 21,776 cases, 19,612 controls) revealed 26 and 2 GWS loci, respectively (Supplementary Data 6). In META_M, 3 loci were novel -replicated, and 14 were novel unreplicated. In META_W, two loci were novel unreplicated. There were no GWS loci in the EUR women, AFR men, and HIS women. QQ and Manhattan plots for all strata are presented in Supplementary Fig. 2(a–l) and Supplementary Fig. 3(a–l), respectively. Collectively, we identified 106 GWS loci across all nine GWAS and four meta-analyses, with some loci exhibiting overlap in genomic position while yielding different lead SNPs for various analytic strata. In total, 49 *distinct* loci across the different strata were identified (Fig. 1B, Supplementary Data 5).

We identified numerous shared loci across sex and ancestry (see Fig. 1B, Supplementary Data 5, Supplementary Data 6), with 11 loci consistently GWS in EUR_M, EUR_C, META_M, and META_C. These “global” loci can be cross-walked to META_C in Supplementary Data 6. For example, locus global_3 (see META_C_10, Supplementary Fig. 4–f) was novel and unreplicated, with lead SNP rs72712556, while locus global 6 (META_C_14, Supplementary Fig. 4–i) lead SNPs varied by strata (Supplementary Data 6).

To prioritize potentially causal SNPs, we ran fine-mapping on the 23 genome-wide significant loci identified in the European population, using the Sum of Single Effects (SuSiE) model [39]. Credible sets were identified for 21 out of 23 loci (sum of posterior inclusion probability>0.95; Supplementary Data 7). Credible sets contained a median of 9 SNPs (range 1–116). SuSiE identified one locus, EUR_11, with 8 separate credible sets. The presence of multiple credible sets within the same locus indicates allelic heterogeneity and the presence of distinct, statistically independent association signals. Many loci had large credible sets, indicating a diffuse signal that is not well localized. However, one locus (EUR_19), had a single SNP (rs11172113) in the credible set, with PIP = 1.0, indicating strong support for causality. This SNP is in an enhancer region of *LRP1*, a gene that has been previously associated with migraine [23].

### Replication of MVP-migraine genome-wide significant loci

We compared our findings to seven previous migraine studies (from non-overlapping cohorts, available through the GWAS catalog) to classify GWS loci identified in this study as known or novel [21, 23, 25, 26, 28, 30, 40]. GWS loci identified in our study were classified as known if any SNP in the locus around the lead SNP (locus area defined by FUMA’s LD clumping algorithm) was found to be genome-wide significant in a previous migraine study. Otherwise, the GWS loci in our study were classified as novel. Supplementary Data 6 presents all loci, genes, and replication status by strata. Of the 49 GWS loci in the MVP cross-strata analysis (Fig. 1B), 13 had prior associations with migraine, and 36 were new to this study (Fig. 1A; Supplementary Data 6). We used a previous large-scale GWA meta-analysis combining GERA and UKB data [21] to replicate the novel loci. Among the 36 new loci, seven loci contained at least one SNP that was nominally significant (after Bonferroni correction for 36 loci tested: *p* < 0.05/36). We label these seven loci ‘novel-replicated’. A further 23 loci contained at least one SNP with *p* < 0.05, but did not remain significant after Bonferroni correction (Supplementary Data 6) [21]. Finally, seven GWS loci were novel to the current study and did not replicate in the GERA-UKBB cohort (all *p* > 0.05). All seven loci had small (ORs near one) and non-significant effects, with three loci showing concordant effect direction and four showing opposing direction. This suggests that the differences may be due to statistical noise, though other sources of heterogeneity are possible. Within the 36 multi-ancestry meta-analyses (META_C) GWS loci, 12 were known (previous GWAS migraine associations), seven had at least one SNP replicate after Bonferroni correction in the GERA-UKBB cohort, and 17 were novel to this study (Fig. 1A). Locus Zoom plots are provided for each novel-replicated and novel-unreplicated SNPs (Supplementary Fig. 4a–kk) across all strata and are described in more detail below.

Given the potential differences between the study population (Veterans, mainly men), and previous migraine GWAS, we sought to understand which previously identified migraine loci were replicated in our data. We identified all previously identified SNPs associated with migraine in the GWAS catalog and cross-referenced them with the results from the current study (using meta-combined results). There were 180 SNPs associated with migraine in the GWAS catalog which were not overlapping a GWS locus from the current study. Of these 180 SNPs, 24 replicated after Bonferroni correction (*p* < 0.05/180), and an additional 65 SNPs were nominally significant in the study data (*p* < 0.05) (Supplementary Data 8). In particular, rs1003194 was Bonferroni-significant in our study (*p* = 0.0015), and was highlighted in a recent large migraine GWAS, mapped to *CALCA/B*, and proposed as a target of new migraine therapeutics [23].

### Genes and pathways mapped to novel and known loci

In the MVP cross-strata analysis incorporating all individual GWAS and meta-analysis results, we identified 283 genes associated with migraine (see Supplementary Data 9 for 188 genes from the META_C results). Of these, 76 genes mapped to the 13 known loci, 61genes mapped to the seven novel-replicated loci, and 146 genes mapped to the 29 novel-unreplicated loci. Among the 13 known loci were well-documented migraine genes (Supplementary Data 10), including *LRP1*, *TRPM8, PRDM16*, *ASTN2*, and *PHACTR1*, all found to be disease-associated genes in the DISEASES database migraine gene set [52] and identified in previous migraine GWAS [25, 30]. Among the genes mapped to novel replicated loci were *CELF4, CAV2*, and *FAM167A* (Supplementary Data 6 and 10). Notably, seven genes mapped to novel loci had been previously associated with migraine in GWAS, including *LINGO2* and *HTRA1*. In these cases, the loci we identified were novel, but the mapped gene was not novel. We also note that several genes we mapped to known migraine loci had not been previously linked with migraine, including *ABCC3* and *PARVB* (Supplementary Data 10). These differences may reflect differences in gene-mapping strategies.

Functional enrichment analysis of the mapped genes revealed significant association with gene sets from human disease databases, including lipidosis, triglycerides, and obesity (DisGeNet), the mammalian phenotype ontology, including decreasing levels of triglycerides and glucose (MPO), cholesterol metabolic process (GO), and other GWAS traits from UKBB and GWAS catalog, including irritability, neuroticism, and fatty acid levels (Supplementary Data 11, Fig. 2A).

### Gene tissue expression and pathway analysis

**Fig. 2:**
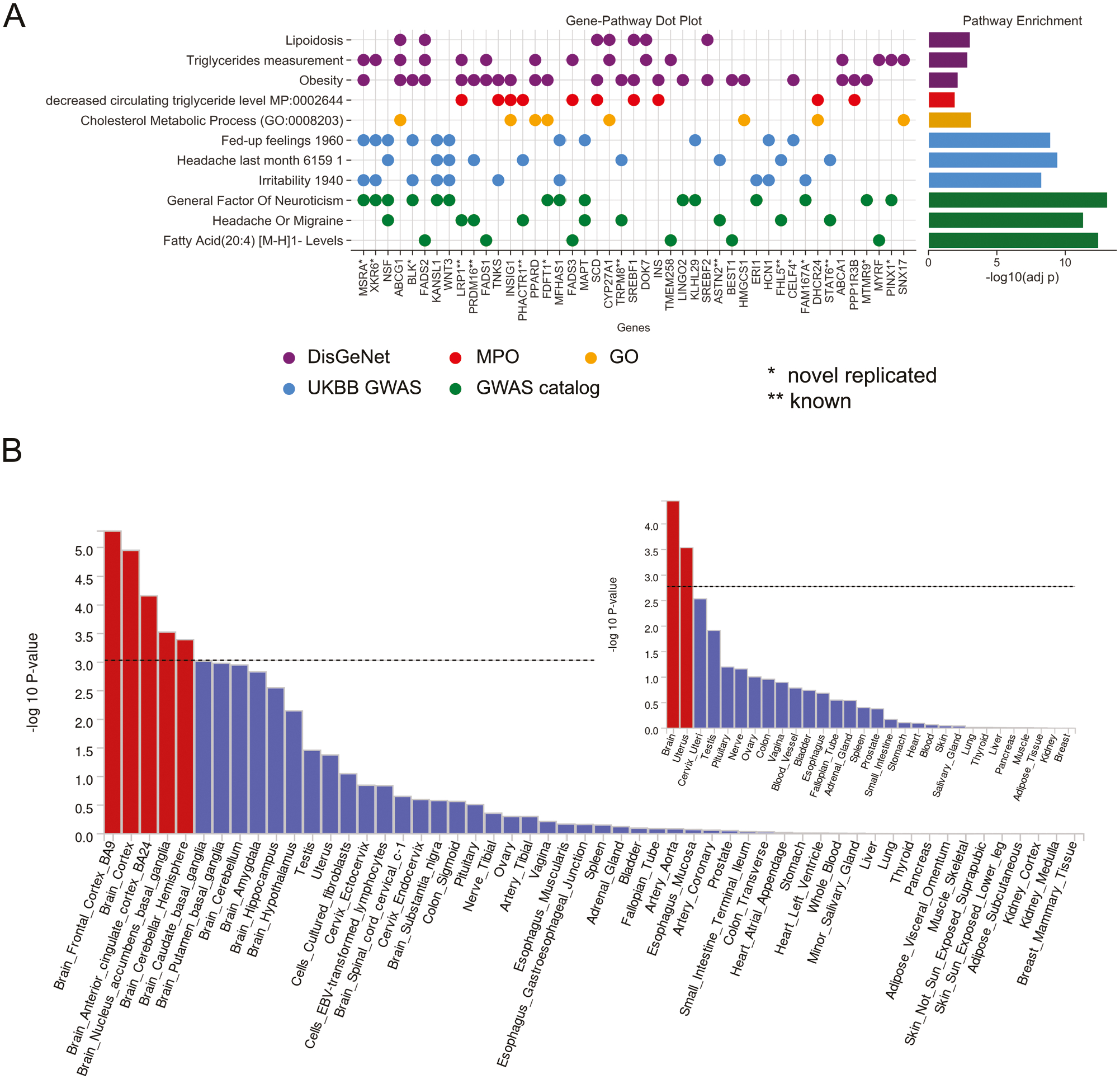
Tissue Expression and functional enrichment analysis of MVP migraine-mapped genes. **A** *The dot plot shows genes in pathways of interest significantly enriched in the genes associated with migraine GWS loci. Genes are shown if they were found in at least two traits selected*. ** indicates a gene from a known migraine locus. * Indicates a gene from a novel-replicated locus. The bar chart on the right side depicts the negative log p-value of the enrichment of the pathway with the genes associated with GWS migraine loci in the study cohort. **B** *MAGMA tissue expression results for multi-ancestry meta-analysis for aggregated (inset) and specific tissues using GTEx v8 data sets. The y-axis represents −log10 p values from one-sided t-tests. The dotted line shows Bonferroni-corrected significance. Findings show enrichment in the uterus and in five regions of the brain*.

Tissue-specific enrichment analysis of the multi-ancestry meta-analysis (META_C) using MAGMA revealed significant associations with brain tissues, including the frontal cortex, cortex, anterior cingulate cortex, nucleus accumbens, basal ganglia, and the cerebellar hemisphere (Fig. 2B; Supplementary Data 12). MAGMA tissue results are presented in Supplementary Data 12 for META_C and Supplementary Data 13 for META_EUR, with figures for all strata presented in Supplementary Fig. 5 a–l. Consistent with the MAGMA pathway results, DEPICT (Supplementary Data 14), an alternative method for identifying enriched pathways and gene sets, identified 17 brain regions as significantly enriched, largely consistent with the MAGMA results. Enriched brain regions included the cerebrum/cerebral cortex, parietal lobe, telencephalon, and temporal lobe.

MAGMA identified one significant gene set (at FDR adjusted threshold *p* = 0.05/19054 gene sets = 2.62 × 10^−6^) associated with cytotoxic T lymphocyte function and immune regulation (2.32 × 10^−08^, Supplementary Data 15). While no pathway met significance criteria following FDR adjustment (*p* = 0.05/14465 gene sets = 3.46 × 10^−6^), the most enriched DEPICT gene set term was the mammalian phenotype ontology term “increased brain weight” (*p* = 2.4 × 10^−5^; Supplementary Data 14). Notably, DEPICT does not consider the direction of effect.

It is noteworthy that none of the arterial tissues achieved nominal significance in MAGMA (Fig. 2B) and DEPICT (Supplementary Data 14) showed no vascular pathways (including NOTCH signaling subnetworks). This finding contrasts with earlier migraine GWAS [23, 30], which reported strong associations with vascular pathways such as NOTCH.

### SNP-based heritability

The SNP-based heritability (*h*^*2*^_SNP_) of MIG-MVP was estimated using LDSC. For the liability scale *h*^*2*^_SNP,_ we used a prevalence of 8.2% for men and 30.1% for women, with a prorated combined lifetime prevalence of 10.0%. Liability scale h2_SNP_ (Supplementary Data 16) was estimated at *h*^*2*^_SNP_ = 0.098 (SE = 0.005) for the combined EUR sample, *h*^*2*^_SNP_ = 0.100 (SE = 0.005) for EUR men, and h2_SNP_ = 0.155 (SE = 0.031) for EUR women.

### Sex-stratified GWAS results

The genetic correlation between EUR_M and EUR_W for migraine was estimated at r_g_ = 0.93 (SE = 0.07), indicating high trait similarity across sexes. When evaluating sex-stratified GWAS analyses, we observed six GWS loci specific to META_M (Supplementary Data 5), which may contain sex-specific signatures. Locus META_M_18, (Supplementary Fig. 4–cc) is novel to our study but was nominally replicated in a previous migraine GWAS [21]. The lead SNP in this locus falls in an exonic region of the gene *AHNAK*. Multiple SNPs in this locus have high CADD scores (> 20), indicating the potential for impact on gene function. Other novel loci specific to men included the unreplicated META_M_5, (Supplementary Fig. 4–y) and associated with *KIF3C, RAB10, EPT1, DRC1, MAPRE3, KHK, SNX17* associated with diabetes, and C-reactive protein function. In addition, unreplicated META_M_24, (Supplementary Fig. 4–ee), is associated with *LAMP5* and *PAK7*, both associated with depression.

The women sample was underpowered (cases = 21,776, controls = 19,612), comprising only 9% of the MVP population and revealing only two novel GWS loci. Both loci were novel and unreplicated (Supplementary Data 6). META_W_2 (Supplementary Fig. 4–ff), is associated with *LINGO2*, a gene previously associated with migraine [21, 23]. META_W_1 (Supplementary Fig. 4–gg) is associated with *CMTM1* and *CMTM3*, genes not previously associated with migraine. In addition, loci associated with migraine in our and previous GWAS trended towards significance in the meta-analysis of women in the MVP (Supplementary Data 5; META_W [e.g., rs11172113, *p* = 1.42E-06 on *LRP1*]).

We evaluated the sex-stratified results of four SNPs reported in the GERA-UKB migraine GWAS [53]. Choquet et al. reported rs1047891*(CPS1)*, rs11718509 *(PBRM1)*, and rs10150336 *(SLC25A21)*, rs7858153 *(ASTN2)* as significantly associated with migraine in women (*P* < 5.0 × 10^−8^) but not men. We evaluated these variants within the MVP men and women cohorts. One of these variants trended toward significance in the MVP META_W sample (rs11718509; *p* = 0.009) and two towards significance in META_M (rs7858153, *p* = 0.002; rs1047891, *p* = 0.059).

### Ancestry-stratified results

One locus was specific to AFR Women (AFR_W_1, Supplementary Fig. 4–jj) with lead SNP rs2864065 associated with *LSAMP* linked to neuronal activity within the limbic system and metabolic syndrome and body mass index. A locus in the AFR Combined strata (AFR_C_1, Supplementary Fig. 4–ii) coding for *IRX1* that may be involved in the development of the nervous system. A locus in the HIS men strata (HIS_M_1, Supplementary Fig. 4–ii) was associated with seven genes, including *RNF4*, previously associated with back pain, *TNIP2*, associated with inflammation, and *GRK4* involved in G protein-coupled receptor signaling and vascular regulation. None of the non-EUR lead variants showed nominal significance in any of the EUR strata (all *p* > 0.05).

### Genetic correlations with psychiatric disorders and brain regions

**Fig. 3:**
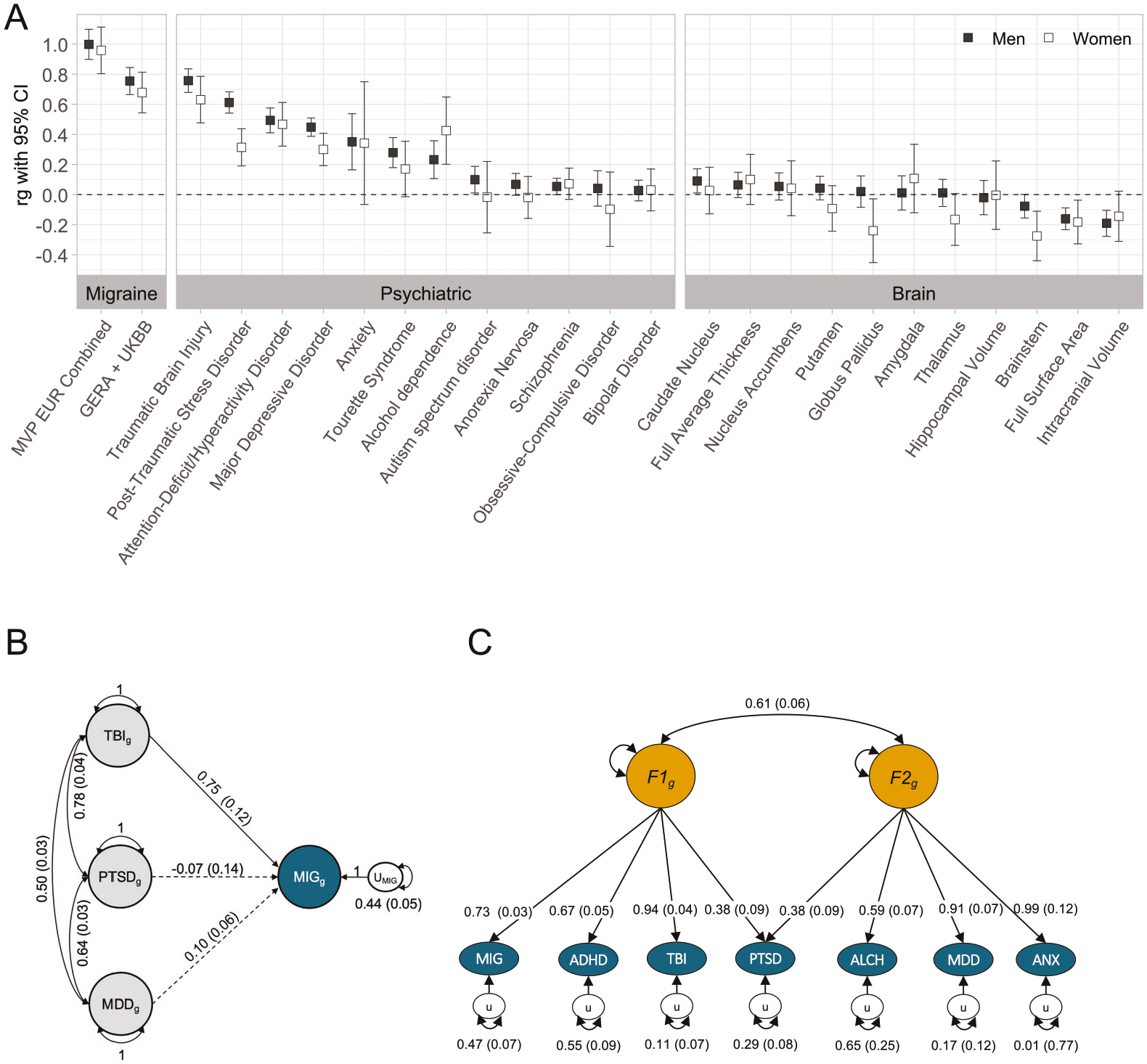
Relationship to other traits. **A** *Genetic correlations between MVP migraine (men and women) and other traits, including GERA-UKBB migraine, EUR_M, EUR_W, psychiatric disorders, and brain metrics. Error bars indicate 95% CI. Black squares represent EUR_M, and white squares represent EUR_W*. **B** *Genomic Structural Equation Modeling (GSEM) path model with standardized estimates and standard errors of the shared genetic architecture of EUR_C migraine, traumatic brain injury (TBI), post-traumatic stress disorder (PTSD), and major depressive disorder (MDD)*. This path model shows the genetic association between migraine (MIG, in blue) and TBI, PTSD, and MDD. Straight arrows represent partial regression coefficients, and bidirectional curved arrows indicate genetic correlations among TBI, PTSD, and MDD. Dotted lines are non-significant; solid lines are significant at p < 0.001. When modeled together, 56% of the genetic variance in MIG is shared with TBI, PTSD, and MDD, and U_MIG_ captures the unexplained variance in MIG (0.44, corresponding to 44%). **C** Best-fitting confirmatory factor analysis (CFA) path model and standardized estimates of MVP EUR_C migraine (MIG) and six psychiatric disorders. Multivariate LD-score regression of odd chromosomes informed the CFA model based on the covariance matrix of even chromosomes. ADHD attention-deficit/hyperactivity disorder; TBI traumatic brain injury; PTSD post-traumatic stress disorder; ALCH problematic alcohol use; MDD major depressive disorder; ANX anxiety. Two correlated genetic factors (F1_g_ and F2_g_) capture the genetic liability shared by the conditions. Straight arrows represent partial regression coefficients and standard errors, capturing the degree of association between a latent genetic factor and each disorder, and bidirectional curved arrows indicate the genetic correlation between latent factors. The proportion of genetic variance in each disorder explained by the latent factors can be calculated by squaring the factor loading (e.g., for MIG = 0.73^2^ = 0.53 or 53%). Genetic variance unexplained by the latent factors in this model is represented by the U_MIG_ oval (e.g., for MIG = 0.47 or 47%). F1_g_ was most strongly associated with TBI, MIG, ADHD, and PTSD, which cross-loaded on both factors. F2_g_ was also associated with ALCH, MDD, and ANX. The model indicated distinct but correlated genetic architecture contributing to these related conditions and suggests that the association with migraine and ADHD, TBI, and PTSD is due to shared genetic variants, while the association with ALCH, MDD, and ANX is at least in part due to the genetic correlation between the two latent factors rather than direct genetic overlap.

We evaluated LDSC genetic correlations between the EUR cohorts and meta-analysis with summary statistics from the PGC, TBI, and brain structure imaging regions from ENIGMA. Within the psychiatric domain (Supplementary Data 17, Fig. 3A), significant correlations ranged from r_g_ = 0.27 (SE = 0.05) for Tourette Syndrome to r_g_ = 0.76 (SE = 0.04) for TBI for the MVP EUR_M sample. Within the brain imaging data (Supplementary Data 17, Fig. 3A), significant correlations ranged from r_g_ = −0.28 (SE = 0.08) between EUR_W and brainstem volume to r_g_ = 0.09 (SE = 0.04) in caudate nucleus volume for the EUR_M sample. Significant differences in magnitude between genetic correlations for men and women (Fig. 3A) were observed for MDD (z = 2.34, *p* = 0.02), PTSD (z = 4.11, *p* < 0.001), brainstem volume (r_g_ = 0.20, SE = 0.09), and globus pallidus volume (r_g_ = 0.26, SE = 0.12).

To evaluate the correlation between specific cerebral regions and migraine while accounting for ICV, we simultaneously estimated the relationship between volumes of specific regions and migraine using GSEM path models. This approach incorporated the correlation between the brain region and ICV and the direct effect of ICV. Most cerebral regions did not exhibit a statistically significant association with migraine after adjusting for ICV (Supplementary Data 18, Supplementary Fig. 6). However, a notable positive association with migraine was observed for the caudate nucleus (β = 0.107, SE = 0.043, *p* = 0.012), suggesting that larger CN volume may correlate with an increased risk of migraine.

### Genomic structural equation modeling

To further explore the shared genetic architecture of migraine and common Veteran migraine comorbidities of TBI, PTSD, and MDD, we modeled the associations simultaneously using a GSEM path model (Fig. 3B) in EUR_C based on LDSC-derived correlations. This approach included both the genetic associations of TBI, PTSD, and MDD with migraine while accounting for the genetic correlations among these traits. Our findings suggest that MDD and PTSD show marginally significant (β = 0.10, SE = 0.06, *p* = 0.07) and non-significant (β = −0.07, SE = 0.14, *p* = 0.644) associations with migraine, respectively, while TBI, even after accounting for MDD and PTSD (which are genetically correlated, r_g_ = 0.64, SE = 0.03), maintains a strong association (β = 0.75, SE = 0.12, *p* < 0.001). Despite the strong genetic associations between migraine and TBI, PTSD, and MDD (Fig. 3A), only the association with TBI remains influential when modeling these conditions simultaneously.

We conducted EFA and CFA on EUR_C migraine and psychiatric disorders with strong genetic correlations (Fig. 3A). Parallel analysis (Supplementary Fig. 7) indicated the presence of one to two factors, and we evaluated one-, two-, and three-factor EFA factor loadings, comparing the corresponding CFA models for the EUR_C migraine sample. EFA factor loadings for two and three-factor models are presented in Supplementary Data 19A, and CFA model fit statistics in Supplementary Data 20A. The one-factor CFA model provided an adequate fit to the data (df = 14, AIC = 75.818, SRMR = 0.1203), but the two-factor model provided the best fit with a lower AIC and improved SRMR (df = 12, AIC = 43.184, SRMR = 0.0471). The three-factor model introduced an additional not-identified factor with a single trait (PTSD) and only a slight improvement in SRMR but an increase in AIC (df = 11, AIC = 44.032, SRMR = 0.038). Consequently, the two correlated factor model provided the most parsimonious and best-fitting solution. (Fig. 3C) The first latent factor (F1) was associated with migraine and neuropsychiatric disorders (ADHD, PTSD, and TBI), while the second factor was associated with anxiety/stress, mood, and alcohol use disorders (ALCH, MDD, PTSD, and ANX). The two latent factors were correlated at r = 0.61 (SE = 0.06), showing a moderate positive relationship between them. The model explained 53% of the genetic variance in EUR_C migraine. As a sensitivity analysis, we repeated the EFA and CFA using the EUR_M and EUR_W GWAS results, both of which indicated that the two-factor model, with similar factor loadings (Supplementary Data 19B–C), provided the best fit (Supplementary Data 20B–C).

### Genetic overlap of migraine with other traits

We conducted an unbiased LDSC screen of genetic correlations with 844 publicly available GWAS using the Complex Trait Genetics Virtual Lab (CTG-VL) [46]. These data include phenotypes from the UK Biobank, GIANT consortium, Psychiatric Genomics Consortium (PGC), FinnGen, and CHARGE, identifying 305 significant genetic correlations with migraine (Bonferroni-corrected *p* < 5.92 × 10^−5;^ Supplementary Data 21). Notable correlations were observed between migraine and multisite chronic pain (r_g_ = 0.71, SE = 0.02), absence of pain (r_g_ = −0.67, SE = 0.03), back pain (r_g_ = 0.54, SE = 0.03), headache in the last month (rg = 0.71, SE = 0.04), hip pain (r_g_ = 0.51, SE = 0.04), as well as medication use including paracetamol (r_g_ = 0.65, SE = 0.04; r_g_ = 0.68, SE = 0.04), codeine-acetaminophen (r_g_ = 0.72, SE = 0.06), and omeprazole (r_g_ = 0.67, SE = 0.05). Mental health conditions included MDD (r_g_ = 0.53, SE = 0.03) and ADHD (r_g_ = 0.47, SE = 0.03). We observed modest but significant associations with triglycerides (r_g_ = 0.153, SE = 0.033) and circulating calcium (r_g_ = 0.091, SE = 0.021), as well as a negative correlation with HDL cholesterol (r_g_ = −0.156, SE = 0.036), while the correlations with total and LDL cholesterol were not significant. Additionally, our results showed positive genetic correlations between migraine and cardiovascular disease, including stroke (r_g_ = 0.544, SE = 0.124), myocardial infarction (r_g_ = 0.229, SE = 0.045), and angina (r_g_ = 0.327, SE = 0.042). Finally, general health indicators including long-standing illness or disability (r_g_ = 0.52, SE = 0.03), and impairment factors, including the inability to work due to health-related issues (r_g_ = 0.62, SE = 0.04) and financial difficulties arising from illness (r_g_ = 0.55, SE = 0.03), were also associated.

**Fig. 4:**
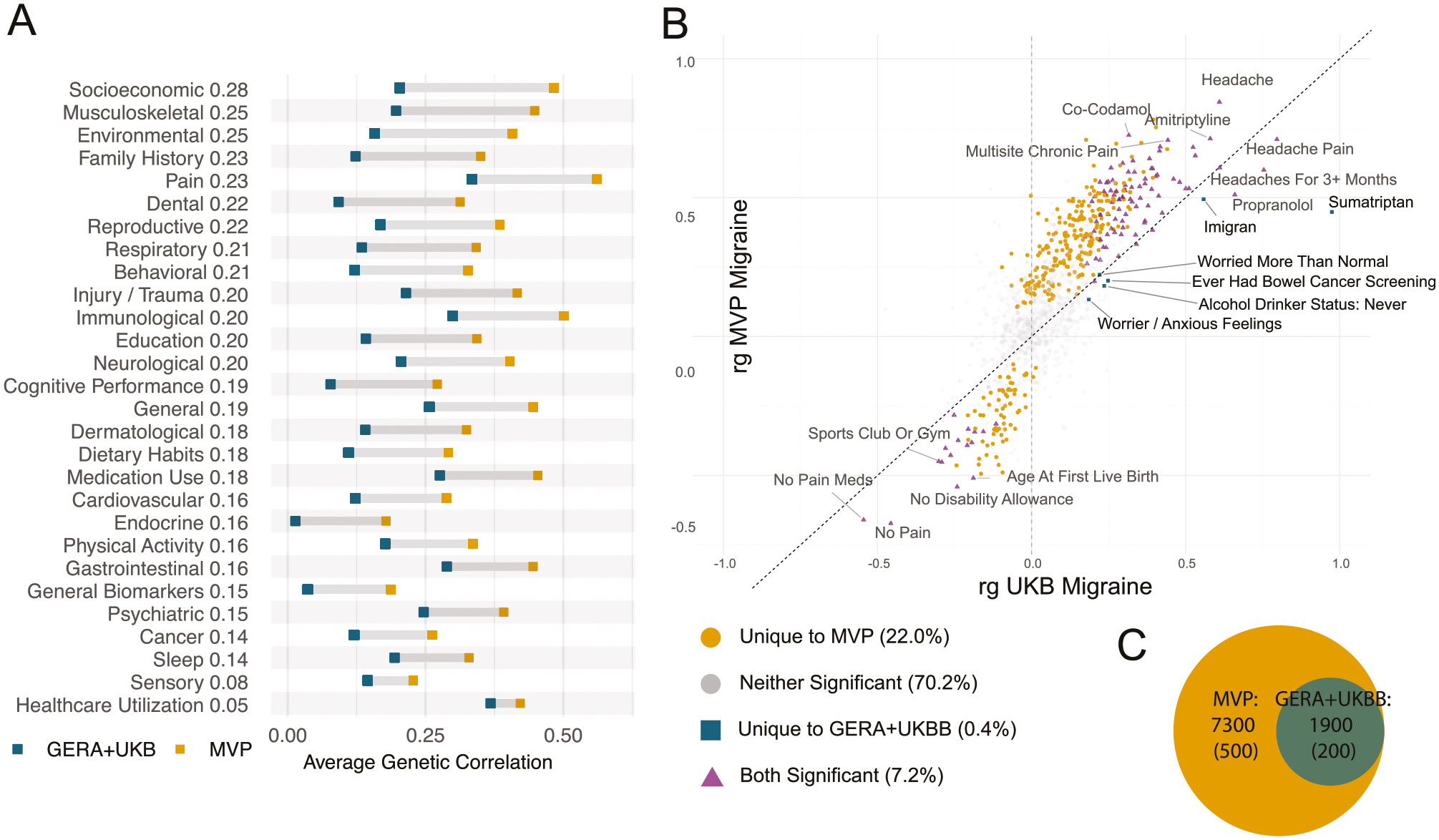
LDSC regression genetic correlations. **A** Average genetic correlation for groups of traits, for MVP (yellow) and blue (GERA-UKBB). Trait groups are sorted by those that differ most between MVP and GERA-UKBB. **B** A scatterplot showing the genetic correlation for all traits tested with GERA-UKBB migraine (x-axis) and MVP migraine (y-axis). The dotted line indicates x = y. Yellow circles are those traits that are uniquely significant in MVP; blue squares are those traits that are uniquely significant in GERA-UKBB; purple triangles represent traits that are significantly correlated with both GERA-UKBB and MVP-migraine traits, while gray circles are those traits that are significantly correlated with neither GERA-UKBB nor MVP-migraine. **C** Genetic overlap between MVP-migraine and GERA-UKB-migraine, computed with MiXeR. The Venn diagram depicts the number of causal variants (standard error) related to MVP-migraine (blue circle) and GERA-UKB-migraine trait (orange circle). All GERA-UKBB causal variants are contained within the set of MVP causal variants. Complete results in Supplemental Fig. 8.

We compared the genetic correlation from the unbiased LDSC screen between the MVP migraine cohort and the GERA-UKBB cohort. The genetic correlation between MVP EUR_C and the GERA-UKB migraine meta-analysis [53] was r_g_ = 0.76 (SE = 0.04). As expected, the MVP EUR_C and GERA-UKB showed similar associations related to headache pain (Fig. 4B; Supplementary Data 21). MVP EUR_C also showed associations with multisite chronic pain (r_g_ = 0.563, 0.055, *p* = 8.68 × 10^−25^) and lower back pain (r_g_ = 0.524, 0.060, *p* = 2.22 × 10^−18^) while GERA-UKB did not. We discerned differences unique to the MVP cohort, encompassing socioeconomic factors, musculoskeletal characteristics, and pain-related traits (Fig. 4A and B). Consistent with the genetic correlations with PGC data (Supplementary Data 17), the r_g_ data for GERA-UKB (Supplementary Data 17), indicates minimal significant correlations with psychiatric conditions from CTG-VL.

### Mendelian randomization

Next, we evaluated the potential causality between traits with nine significant genetic correlations with MVP migraine (Fig. 3A; TBI, ADHD, TS, anxiety, PTSD, alcohol dependence, depression, ICV, surface area) using the CAUSE software for MR. We compared null, shared, and causal models using expected log predictive density (ELPD) differences to test for statistical significance, set at *p* < 0.01. Of the traits tested, none showed evidence of a significant causal relationship on MVP-migraine (Supplementary Data 22), with all *p* > 0.05. TBI sharing model approached statistical significance when compared to the null model (DELPD = −10.50, SE_DELPD_ = 4.31, z = −2.45, *p* = 0.007). Conversely, there was no evidence that MVP migraine caused any of the tested traits (DELPD *p* < 0.05). The causal model did not provide a significantly better fit than shared polygenicity models, thus indicating no evidence that MVP migraine caused any of the tested traits.

### Polygenicity identified in MVP migraine, relative to previous migraine GWAS

We extended our comparison analyses between MVP-migraine and GERA-UKB-migraine genetic architecture using MiXeR, a Gaussian mixture modeling approach to estimate polygenicity. Quantification of the polygenic overlap between MVP-migraine and GERA-UKB-migraine revealed that MVP shared all ~1900 GERA-UKB loci, but there were ~7300 loci predicted to be unique to MVP-migraine (Fig. 4C; Supplementary Data 23, Supplemental Fig. 8). The large number of MVP-migraine unique loci leads us to conclude that MVP migraine is much more polygenic than GERAUKB despite the high genetic correlation (r_g_ = 0.76, SE = 0.04). The additional polygenicity of MVP-migraine could reflect features specific to the MVP, such as the predominantly men Veteran population. The polygenicity comparison of MVP migraine Men_C and Women_C was inconclusive due to the low sample size in the MVP women population.

### Drug-class and drug-set enrichment analyses

We evaluated each gene associated with any GWS loci for its presence within a comprehensive database of known drug targets (OpenTargets Platform) [54]. We identified 76 drugs that are known to target at least one gene identified in our study based on FUMA prioritization (Supplementary Data 24). Seven genes from loci previously associated with migraine in prior studies were linked to ten drugs, including monoclonal antibody drugs (tanezumab, fasinumab, fulranumab) that target nerve growth factor (NGF) function to mediate pain signaling, as well as metformin hydrochloride (which targets *NDUFAF4*), a well-established treatment for type 2 diabetes with possible neuroprotective effects and previous unsuccessful clinical trial (NCT02593097) for migraine, in addition to menthol, which targets TRPM8. Six novel loci from our study were linked to several medications associated with established migraine pathways. These include TLR4 antagonists (eritoran, resatorvid), which play a role in neuroinflammation targeting *TLR4*; p38 mitogen-activated protein kinase inhibitors (losmapimod, doramapimod, neflamapimod) targeting *MAPK14*; and peroxisome proliferator-activated receptor agonists (bezafibrate, seladelpar, lanifibranor) targeting *PPARD*. Nine novel loci mapped to additional drugs, including calcium channel modulators used in migraine treatment, GABA analogs used for pain management, and immunomodulators.

## DISCUSSION

Our multi-ancestry meta-analysis GWAS of migraine in the predominantly male MVP sample identified 49 GWS across all strata, including 36 loci novel to this study. Of the 36 novel loci, 7were novel and nominally-replicated with a previous migraine GWAS by Choquet et al. [21, 53]. The previously known loci mapped to 76 genes, novel-replicated loci mapped to 61genes, and novel-unreplicated loci mapped to 146 genes. Among individual strata results, most of the genetic discovery was driven by the EUR strata (23 loci), as the AFR and HIS ancestries were underpowered due to smaller sample sizes. In the cross-ancestry meta-analysis, the power to detect GWS variants was increased with the inclusion of AFR and HIS ancestries, and we identified 36 GWS loci, of which 12 loci had previous associations with migraine, including rs11172113, near *LRP1*, rs2864065 *LSAMP*, and rs61759167, near *PRDM16* [30, 55]. An additional 7 GWS loci replicated nominally, and 17 GWS loci were novel to this study. Two loci were GWS in the meta-analysis of women, a sample that was also underpowered compared to the men.

The 207 genes associated with migraine in novel loci in this study included *MAML3, CELF4, IRX1, ASXL1, SPOCD1*, *CXCL*, and *TLR4*. Notably, *MAML3* has previously been associated with chronic pain [56], suggesting a potential overlap in genetic mechanisms involving neural and inflammatory pathways between migraine and other pain conditions. *ASXL1* has been associated with decreased circulating triglyceride and glucose levels while *CELF4* was associated with a GWAS of “fed-up feelings” and obesity [57]. The *CELF4* locus identified as GWS in multiple strata and found in the promoter region of the gene contained multiple SNPs with high CADD scores (>15), indicating a potential regulatory impact on the gene’s expression. Functionally, *CELF4* is important to neuronal function and synaptic signaling and plasticity, mechanisms implicated in migraine physiology [58]. Similarly, *IRX1* is associated with neuronal development and function, suggesting a possible role in neurogenesis and neural signaling in migraine. The *SPOCD1* (rs329117) locus is associated with transcriptional regulation and cellular stress responses. *CXCL5* is a chemokine that regulates inflammatory responses and is an intriguing candidate given the known association of neuroinflammation in migraine. The association of *CXCL5* with vascular inflammation may point to a potential link to the vasodilation and immune activation association with migraine. Similarly, *TLR4* is involved in neuroinflammation and immune signaling, two processes increasingly associated with migraine [59, 60]. *TLR4* has functional and mechanistic links to migraine etiology but has not previously been reported in GWAS [61]. Among the novel-unreplicated loci were additional genes associated with neuronal development and function, including *LINGO2* (rs55938934) and *KIF3C* (rs13007894), cell metabolism including *IGF2* and *INS-IGF2* (rs7482510), and *DHCR24* (rs174529, rs509360) involved in cellular stress response. While these loci are as yet not replicated, they may point to possible pathways and mechanisms to evaluate in future migraine research. Together, these findings underscore the involvement of immune and inflammatory processes in migraine etiology and contribute genetic findings to the neuroinflammatory model of migraine involving vascular, immune, and neuronal dysregulation [62–64]. Identifying novel loci involved in synaptic signaling and neuronal plasticity, immune, and inflammatory mechanisms underscores the convergence of neural, immune, and vascular pathways in the etiology of migraine.

Sex and ancestry-specific results were limited due to the limited power in the women and non-EUR strata. We did identify one genomic locus that was GWS in the AFR sample (rs114364083, near *IRX1*) and one genomic locus that was GWS in the HIS sample (rs12453054, near *NSF*). Numerous loci significant in the EUR samples also trended towards significance within the AFR and HIS samples.

Functional characterization showed significant enrichment of multiple brain tissues crucial to migraine pathophysiology, involving pain modulation, sensory integration, and emotional regulation [18]. Enrichment was also observed in the uterus, suggesting a possible link with hormone regulation and sex-specific mechanisms in migraine etiology and potentially reflecting shared pathways with neurovascular and endocrine function. This finding aligns with a recent sex-specific transcriptome-wide association study of migraine using ovary, uterus, and vagina eQTL reference panels that also identified a significant uterine association [65]. Previous findings also show a positive genetic link between migraine and endometriosis, another chronic overlapping pain condition, lending support for shared neuroendocrine biology between migraine and endocrine function [66]. Because several loci responsible for uterine enrichment (e.g., *CELF4, TLR4*) also show significant expression in endocrine and neuroendocrine regions, including the hypothalamus and adrenal gland in GTEx, it is possible that the uterus functions primarily as a sentinel tissue within a broader domain of endocrine regulation that is active in both men and women, rather than signaling a women-specific mechanism. Because the MVP cohort is over 90% men, the uterine enrichment is more likely to reflect shared neuroendocrine pathways instead of direct influences on female reproductive organs. This finding was seen in both the multi-ancestry meta-analysis (META_C) and the men-only trans-ancestry META_Men analysis, highlighting the possibility that the uterine tissue enrichment may be a proxy for a broader biological process that remains unmeasured but is central to migraine pathophysiology. This aligns with the established association between hormone fluctuation and migraine etiology. Together, these findings reiterate the involvement of the central nervous system and hormone function in migraine.

Although previous migraine GWAS revealed a strong vascular connection, including enrichment of arterial tissue and NOTCH pathway genes [23, 30], our findings showed more limited vascular associations. Cohort differences may account for this disparity, including our predominantly male composition, which may obscure vascular loci if they are more pronounced in women, as well as a potentially elevated rate of environmental and trauma exposures among MVP Veterans than primarily community samples used in previous studies. Notably, although we did not observe an enrichment of vascular tissue, we successfully replicated several well-documented migraine loci that are associated with established arterial eQTL effects, including *LRP1, C1orf174, TSPAN2, NGF, PHACTR1, TBC1D7*, and *GFOD1*, suggesting that individual arterial regulatory signals continue to be present, despite the absence of overall tissue-level enrichment. We also observed modest genetic correlations between migraine and circulating biomarkers, including higher triglycerides, lower HDL, and higher circulating calcium, consistent with previous findings of lipid [40, 67, 68] and calcium associations [69]with migraine. Stronger genetic correlations were seen with cardiovascular conditions, including stroke and myocardial infarction, in line with previous findings [70].

Our cohort showed higher polygenicity than prior groups, likely due to the unique Veteran population. This may stem from the predominantly male makeup or characteristics like elevated trauma, PTSD, anxiety, depression, and TBI. Such experiences might enhance the genetic influence on migraine, with novel variants possibly interacting with environmental stressors. The polygenicity in the MVP sample may also be a function of etiologic heterogeneity due to ascertainment challenges using EHR and self-report. Future research incorporating polygenic risk scores may help identify individuals at risk of migraine, and enhance our understanding of migraine comorbidities, and support future personalized prevention and treatment strategies. Additionally, understanding gene-environment interactions between environmental hazards like TBI and migraine can contribute to better risk stratification and clinical decision making.

We observed robust genetic correlations between migraine and psychiatric disorders in line with the body of clinical and epidemiological literature linking migraine to psychiatric disorders, including anxiety disorders [71–73], MDD [74], and PTSD [75, 76], as well as ADHD [77, 78]. These results were consistent with the Brainstorm Consortium findings [79] but generally larger in magnitude (e.g., MDD, TS, ADHD) with MVP analyses also detecting associations with PTSD, anxiety disorders, AUD, and TBI, potentially consistent with the VA comorbidity profile. Our GSEM findings suggest that the association of migraine with TBI, ADHD, and PTSD is due to shared neuropsychiatric genetic architecture. In contrast, the association of migraine with MDD, ALCH, and ANX may be attributed to the genetic correlation between latent genetic architecture rather than direct genetic overlap. Consistent with prior findings [80], our analysis also showed a negative genetic correlation between migraine and ICV and enrichment of the MPO phenotype “increased brain weight.” Our results indicate this association is seen in both men and women (Fig. 3A) and also support a genetic relationship between migraine and brain stem consistent with previously reported volumetric changes in the brainstem in migraine patients [81, 82] and tissue enrichment.

Our analyses identified multiple candidate drugs. Candidates based on loci with prior associations with migraine included monoclonal antibody drugs (tanezumab, fasinumab, fulranumab) targeting *NGF*, which mediates pain signaling, and metformin hydrochloride (targeting *NDUFAF4*). Menthol, targeting TRPM8, is another candidate currently used for acute relief. Several TRPM8 antagonists were evaluated in phase 1 trials, including for the treatment of migraine (NCT01953341), but adverse effects curtailed their development [83, 84]. Extending these results, drugs associated with loci novel to the current study included p38 mitogen-activated protein kinase inhibitors (losmapimod, doramapimod, neflamapimod) targeting *MAPK14*, a gene associated with neuroinflammation. Some of these drugs, like losmapimod, have undergone trials in rheumatoid arthritis and other inflammatory conditions, suggesting potential for modulating neuroinflammatory processes associated with migraine. TLR4 antagonists (eritoran, resatorvid), target inflammation by inhibiting TNF-a and IL-6 production. While these drugs have shown mixed efficacy in treating sepsis and other inflammatory disorders [85, 86] their relevance to inflammatory pathways warrants further investigation in the treatment of migraine. Finally, peroxisome proliferator-activated receptor agonists (bezafibrate, seladelpar, lanifibranor) targeting *PPARD* may address mitochondrial and inflammatory processes involved in migraine. Finally, nine novel unreplicated loci from our results mapped to additional drugs, including calcium channel modulators, GABA analogs, and immunomodulators. Notably, calcium channel blockers have been used in migraine treatment and GABA analogs in pain management. Our findings highlight the strength of this analytical approach in identifying drugs with established relevance to migraine pathophysiology while uncovering new potential therapeutic targets.

To our knowledge, this is the first migraine genomic study of a predominantly male sample. Our multi-ancestry analyses enhance understanding and pave the way for future evaluations. However, the very nature of a veteran-based sample led to several notable limitations affecting power and generalizability. Our men-biased sample led to underpowered analyses for women, resulting in fewer observed GWS loci, particularly none in the EUR women population. Non-European analyses were similarly underpowered, with no GWS loci identified in AFR men or HIS women, although some loci approached significance. Non-EUR ancestries improved overall statistical power but did not clarify ancestry-specific findings. The small population of Asian ancestry Veterans prevented their inclusion in analyses. Future research must include more diverse samples to address sex and ancestry-specific results, including chromosome X data, which was not available at the time of this study. Our phenotype relied on EHR and self-report, which may underestimate migraine prevalence despite strong agreement between methods. While EHR provided a broad history of migraine, different sources and potential biases could lead to inaccuracies. As EHR diagnostic codes capture clinical encounters rather than the initial symptom onset, we did not have the true age of migraine onset, which limited our ability to assess age-related patterns. To prevent control misclassification, we excluded individuals without complete survey data, and ICD codes limited to the Veterans’ VA EHRs might miss historical care. Similarly, individuals with headaches – a proportion of which could be undiagnosed migraine cases – may have been included in the control sample. We were unable to evaluate migraine severity or differentiate between migraine subtypes (migraine with and without aura) because the MVP Baseline Survey did not capture this information, and aura is inconsistently coded in VA EHR data. Future studies can include natural language processing of clinical notes to improve subtype phenotyping. Because detailed information regarding trauma and abuse history, including childhood trauma, sexual abuse, and civilian experiences, was limited in our sample, we relied on PTSD diagnoses as an imperfect proxy in our analyses, noting as we had in our previous work [4] that PTSD rates were higher in those with migraine than in those without. Future studies may explore the relationship between injury, trauma, migraine chronification, and post-traumatic headache through richer phenotyping and targeted assessment. Together, ascertainment and phenotyping limitations may have contributed to phenotypic heterogeneity and in turn increased polygenicity in our sample. Lastly, our replication efforts were limited to publicly available migraine GWAS and significant SNPs from published studies. At the time of analysis, the data provided by Choquet et al. contained full GWAS summary statistics, while other studies were limited to lead SNPs. Future studies can conduct more comprehensive replication as additional results become available.

This work provides valuable insights into migraine, particularly within the predominantly male demographic, by identifying 36 novel loci and new candidate genes. Our study sets the foundation for future ancestry-specific and sex-specific genomic migraine research and points to the role of neuroinflammatory and immune pathways in migraine pathology, consistent with the existing framework of migraine pathophysiology. This study demonstrated the complex interplay between migraine and neuropsychiatric disorders, highlighting the role of the shared genetic architecture that contributes to this relationship.

## Supplementary Material

41380_2025_3392_MOESM1_ESM.docx

41380_2025_3392_MOESM2_ESM.xlsx

**Supplementary information** The online version contains supplementary material available at https://doi.org/10.1038/s41380-025-03392-4.

## Figures and Tables

**Table 1.** MIG-MVP study samples in the discovery GWAS by HARE-derived ancestry and ethnicity.

HARE Ancestry^a^	Total N = 433,010	Cases n = 87,859	Controls n = 345,151	P^b^
EUR (%)	338,743 (78.23)	59,975 (17.71)	278,768 (82.29)	
Age (SD)	65.24 (12.87)	55.85 (14.26)	67.26 (11.60)	<0.001
Men (%)	311,797 (92.04)	46,817 (15.02)	264,980 (84.98)	<0.001
Women (%)	26,946 (7.95)	13,158 (48.83)	13,788 (51.17)	
AFR	65,178 (15.05)	19,358 (29.70)	45,820 (70.30)	
Age (SD)	58.34 (12.07)	51.18 (12.07)	61.37 (10.73)	<0.001
Men (%)	54,172 (83.11)	12,834 (23.69)	41,338 (76.31)	<0.001
Women (%)	11,006 (16.89)	6524 (59.28)	4482 (40.72)	
HIS	29,089 (6.71)	8526 (29.31)	20,563 (70.69)	
Age (SD)	56.93 (14.94)	47.80 (14.14)	60.71 (13.56)	<0.001
Men (%)	25,653 (88.19)	6432 (25.07)	19,221 (74.93)	<0.001
Women (%)	3436 (11.81)	2094 (60.94)	1342 (39.06)	
TOTAL	433,010 (100.00)	87,859 (20.29)	345,151 (79.71)	
Age (SD)	63.64 (13.26)	54.04 (14.08)	66.09 (11.86)	<0.001
Men (%)	391,622 (90.44)	66,083 (16.87)	325,539 (83.13)	<0.001
Women (%)	41,388 (9.56)	21,776 (52.61)	19,612 (47.39)	

a Ancestry based on MVP HARE (harmonized ancestry and race/ethnicity) estimates. *EUR* European HARE-derived ancestry, *AFR* African HARE-derived ancestry, *HIS* Hispanic HARE-derived ancestry.

b *P* Values were based on chi-squared test of independence (migraine) and t-test (age).

## Data Availability

MVP summary data access can be obtained by submitting a data access request through dbGaP; raw data are protected and are not available due to privacy reasons. GWAS and meta-analysis summary statistics will be available in dbGaP (https://www.ncbi.nlm.nih.gov/gap/) upon publication under accession phs001672. GWAS summary statistics data (multiethnic meta-analysis) from the study of Choquet et al. are publicly accessible from the NHGRI-EBI GWAS Catalog (https://www.ebi.ac.uk/gwas/downloads/summary-statistics), study accession number GCST90000016.

## CODE AVAILABILITY

Data analysis was conducted using code from dbSNP, Regenie (https://rgcgithub.github.io/regenie/), Metal (https://github.com/statgen/METAL), LDSC (https://github.com/bulik/ldsc), SuSiE (https://annahutch.github.io/PhD/SuSiE.html), MiXeR (https://github.com/precimed/mixer), CAUSE (https://github.com/jean997/cause), GSEM (https://github.com/GenomicSEM/GenomicSEM) Open Targets (https://platform.opentargets.org), Complex Trait Genetics Virtual Lab (https://vl.genoma.io), ChEMBL (https://www.ebi.ac.uk/chembl/).
