# Supplementary material for: A multi-ancestry meta genome-wide association study of migraine among veterans: associations with traumatic brain injury, depression, and post-traumatic stress disorder
Source: Mol Psychiatry. 2025 Dec 19;31(5):2660–74. doi: 10.1038/s41380-025-03392-4 (PMC12863721; doi:10.1038/s41380-025-03392-4)
Supplement: Supplementary file 1 — Supplementary File [file 41380_2025_3392_MOESM1_ESM.docx]

**Supplementary File**

**Supplementary Figures**

**Supplementary Figure 1.** An example path model.

**Supplementary Figure 2 (a-l).** Quantile-quantile plots, by strata.

**Supplementary Figure 3 (a-l).** Genome-wide association study Manhattan plots of migraine in the MVP, by strata.

**Supplementary Figure 4 (a-kk).** Regional association plots for novel replicated and unreplicated migraine loci.

**Supplementary Figure 5 (a-l).** Tissue expression by strata.

**Supplementary Figure 6.** Path model for the relationship between the caudate nucleus and migraine.

**Supplementary Figure 7.** Parallel analysis scree plot.

**Supplementary Figure 8.** Bivariate causal mixture model results.

**Acknowledgments**

**Supplementary Figure 1.** An example path model to evaluate the relationship between the caudate nucleus (CN) and migraine (MG) while accounting for intracranial volume (ICV) and the correlation between ICV and CN. Straight arrows represent partial regression coefficients, and bidirectional curved arrows indicate genetic correlations. Umig represents the unexplained variance in migraine.


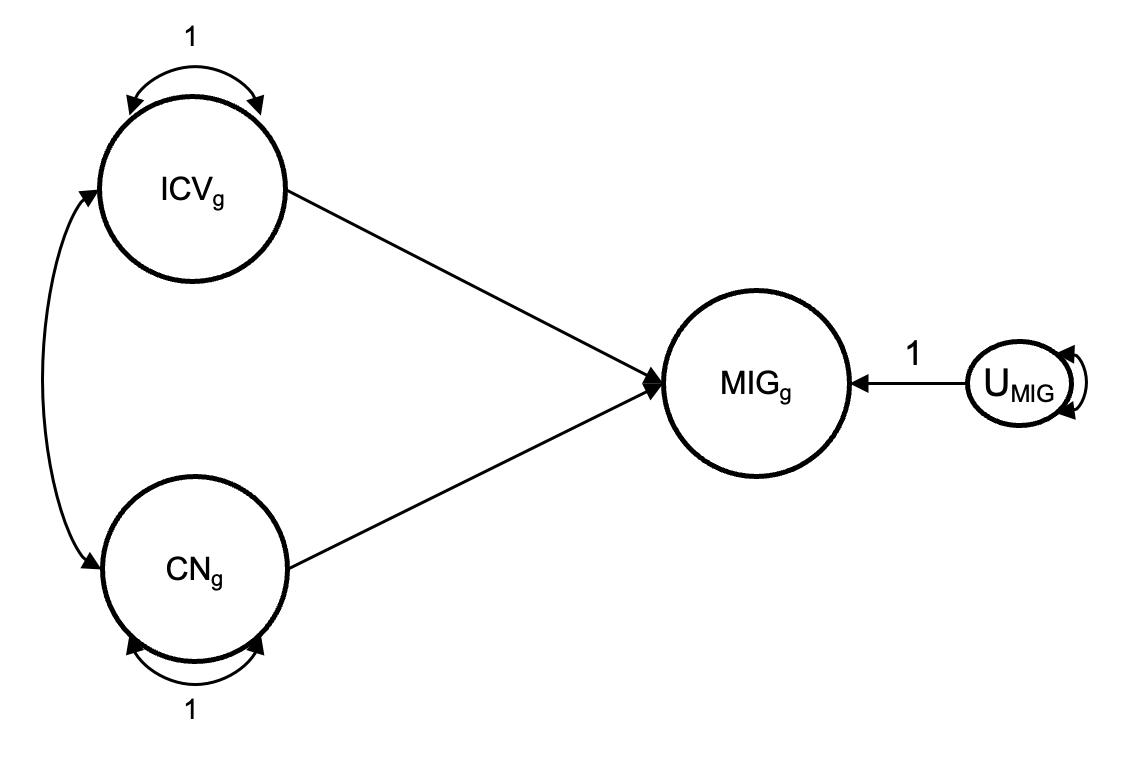


**Supplementary Figure 2.** Quantile-quantile plots of expected vs. observed p values (-log10) for genome-wide association studies of migraine across different strata.

| 1. **EUR men**   **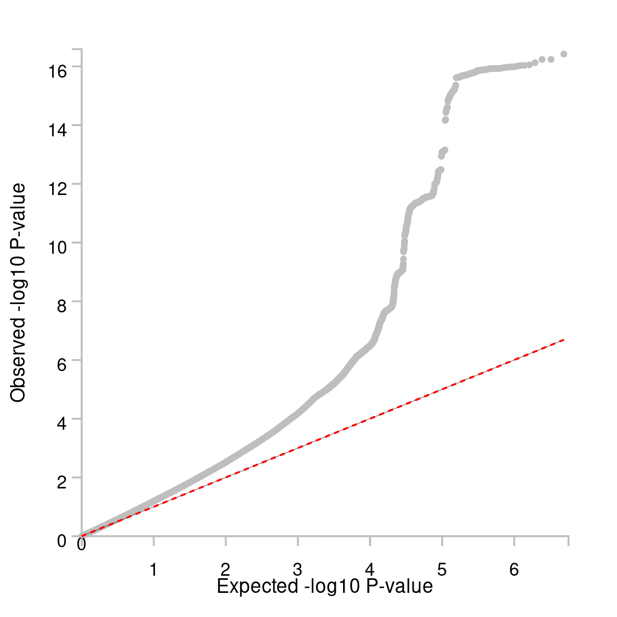**  GCλ = 1.305 | 1. **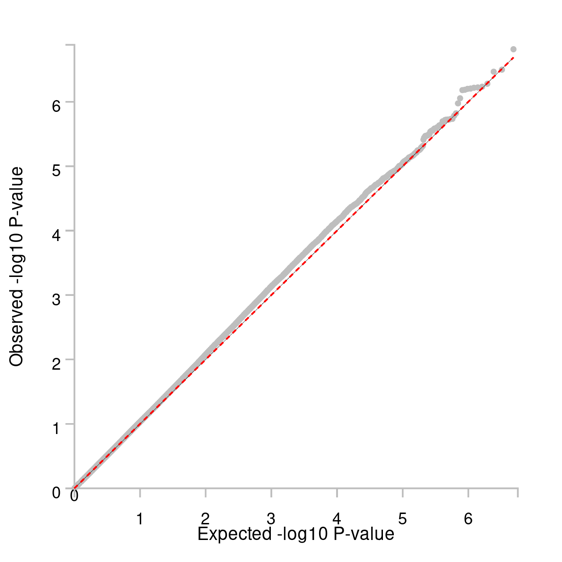EUR women**   GCλ = 1.056 | 1. **EUR combined**   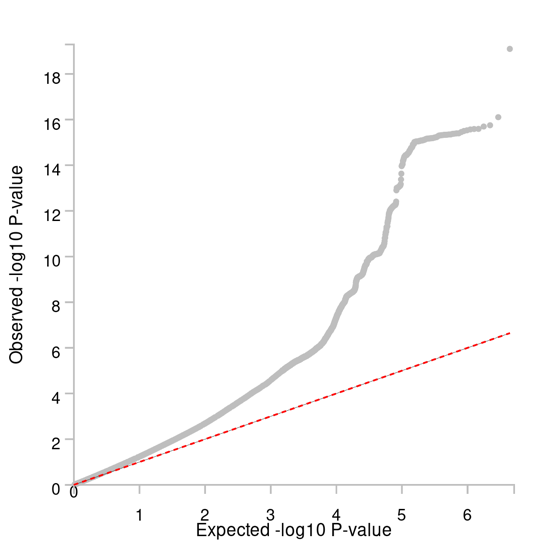  GCλ = 1.341 |
| --- | --- | --- |
| 1. **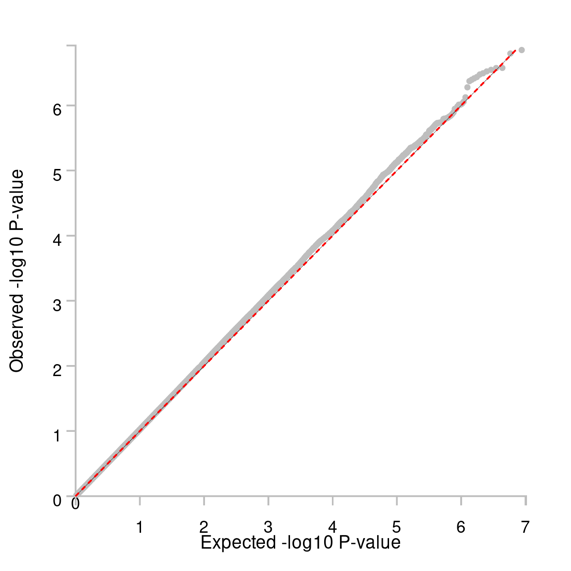AFR men** | 1. **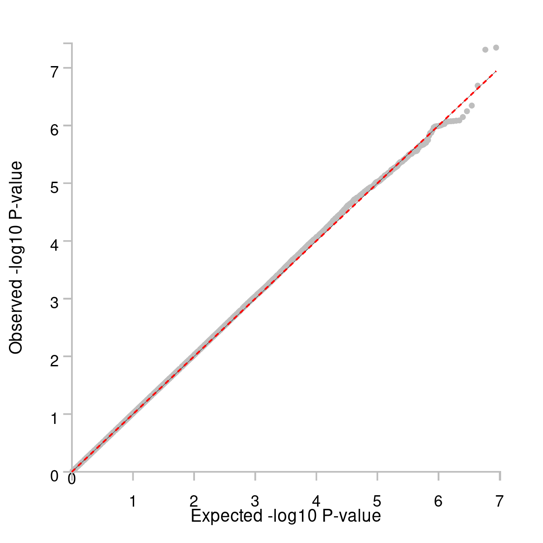AFR women**   GCλ = 1.094  GCλ = 1.022  GCλ = 1.048 | 1. **AFR combined**   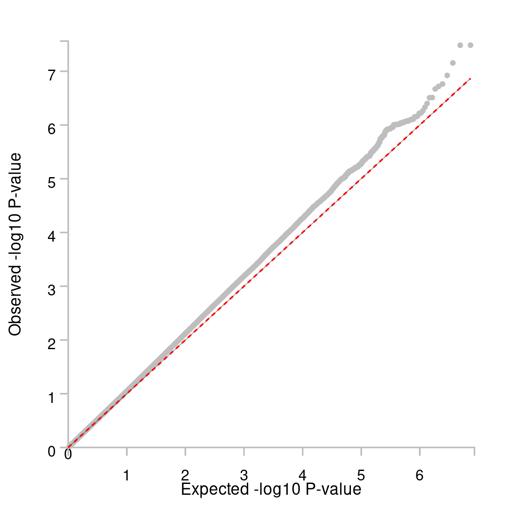 |
|  |  |  |
| 1. **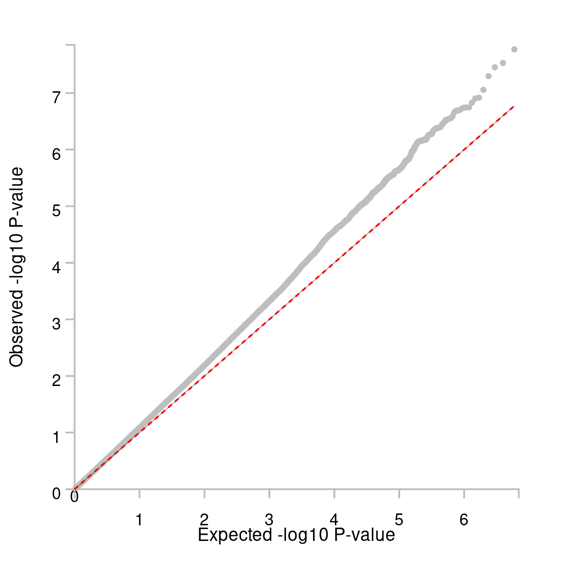HIS men** | 1. **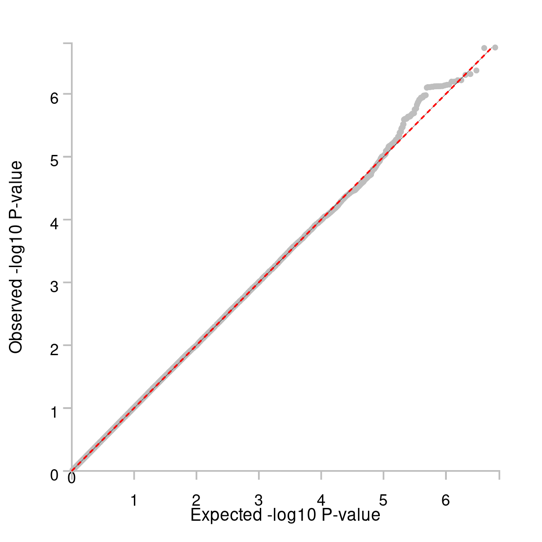HIS women**   GCλ = 1.137  GCλ = 1.013  GCλ = 1.137 | 1. **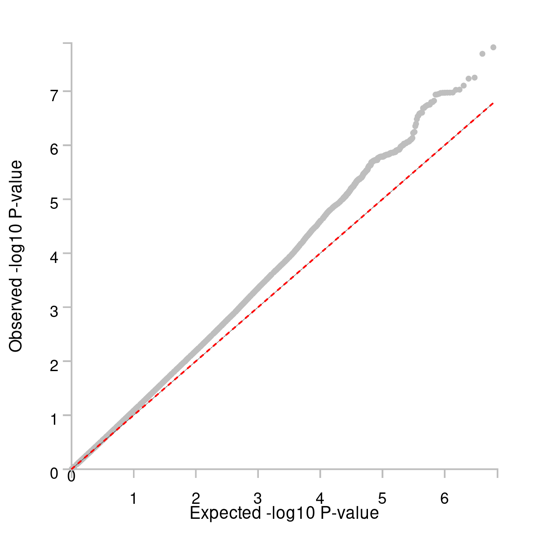HIS combined** |
| 1. **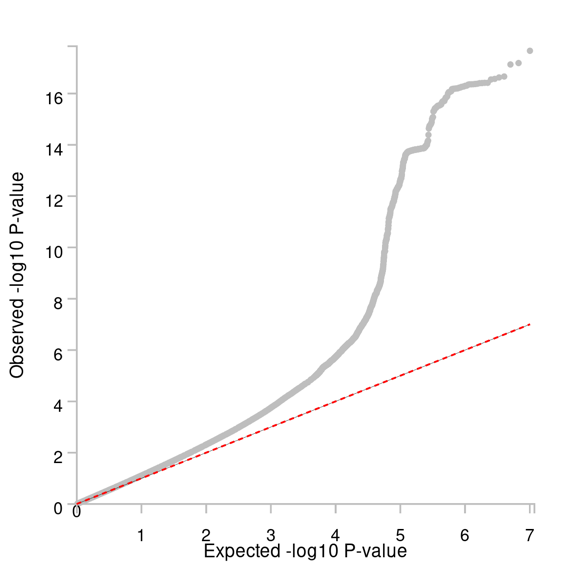META men**   GCλ = 1.318  GCλ = 1.064  GCλ = 1.365 | 1. **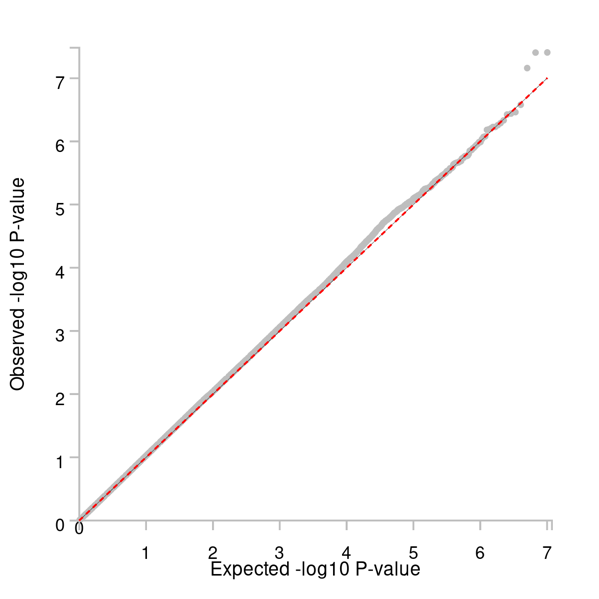META women** | 1. **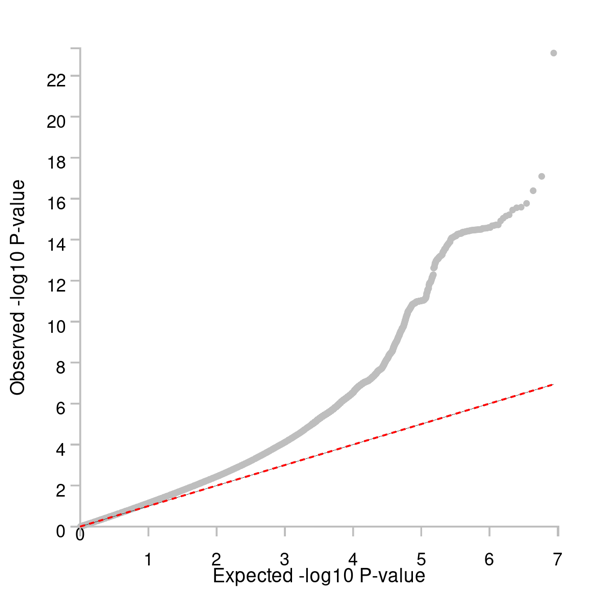META combined** |
|  |  |  |

**Supplementary Figure 3a.** Genome-wide association study Manhattan plots of migraine for **EUR men** strata in MVP. The x-axis represents the chromosome position, and the y-axis represents the GWAS significance level (*p*  on a -log_10_ scale). The dashed red line shows genome-wide significance at p>5x10^-8^.


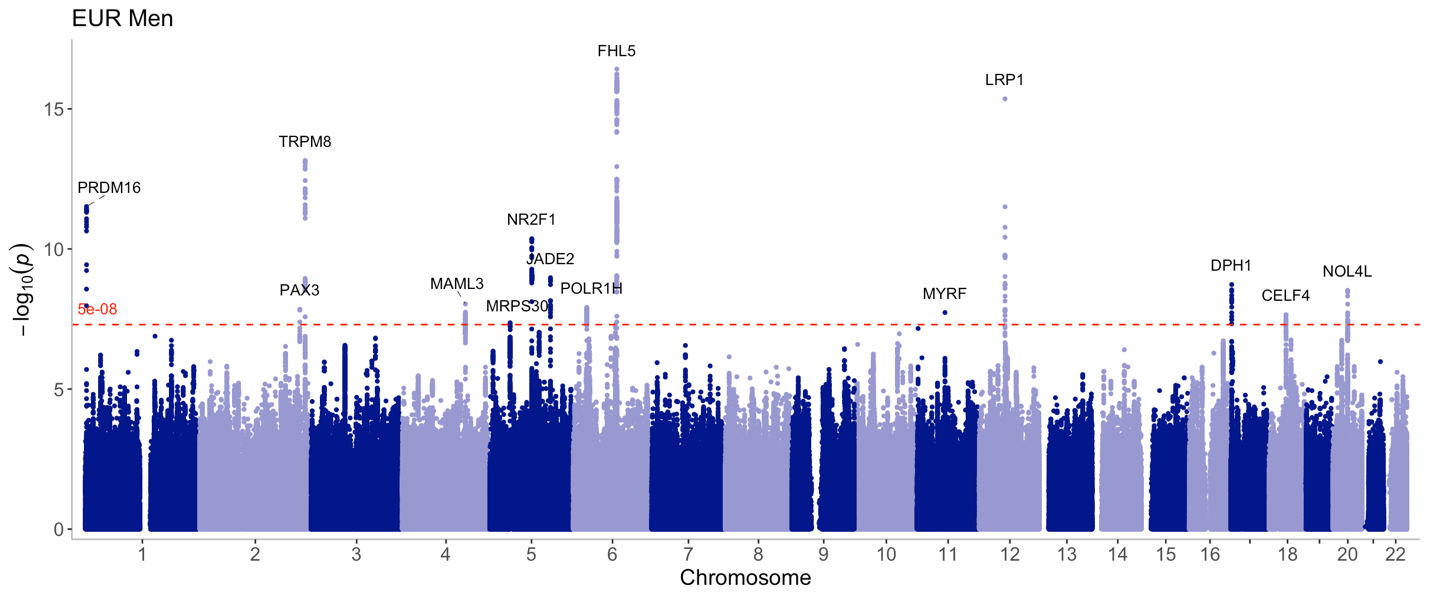


**Supplementary Figure 3b.** Genome-wide association study Manhattan plots of migraine for **EUR women** strata in MVP. The x-axis represents the chromosome position, and the y-axis represents the GWAS significance level (*p* on a -log_10_ scale). The dashed red line shows genome-wide significance at p>5x10^-8^.

**
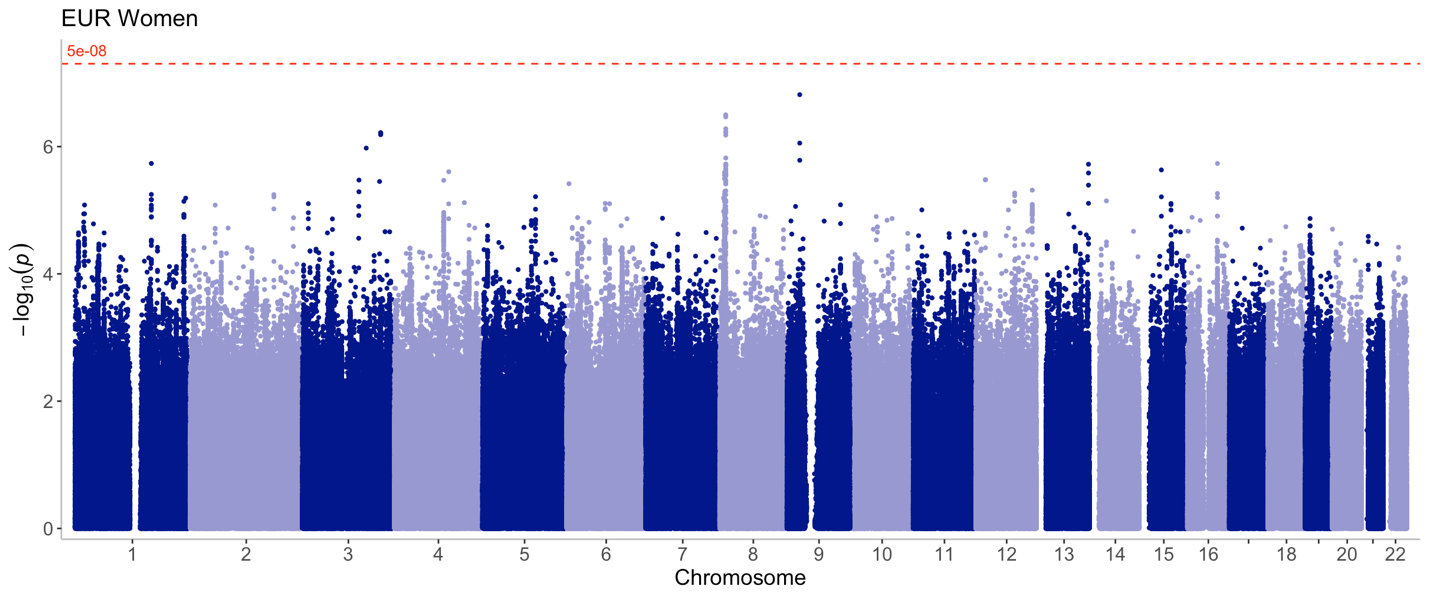
Supplementary Figure 3c.** Genome-wide association study Manhattan plots of migraine for **EUR** **combined** (men and women) strata in MVP. The x-axis represents the chromosome position, and the y-axis represents the GWAS significance level (*p* on a -log_10_ scale). The dashed red line shows genome-wide significance at p>5x10^-8^.

**
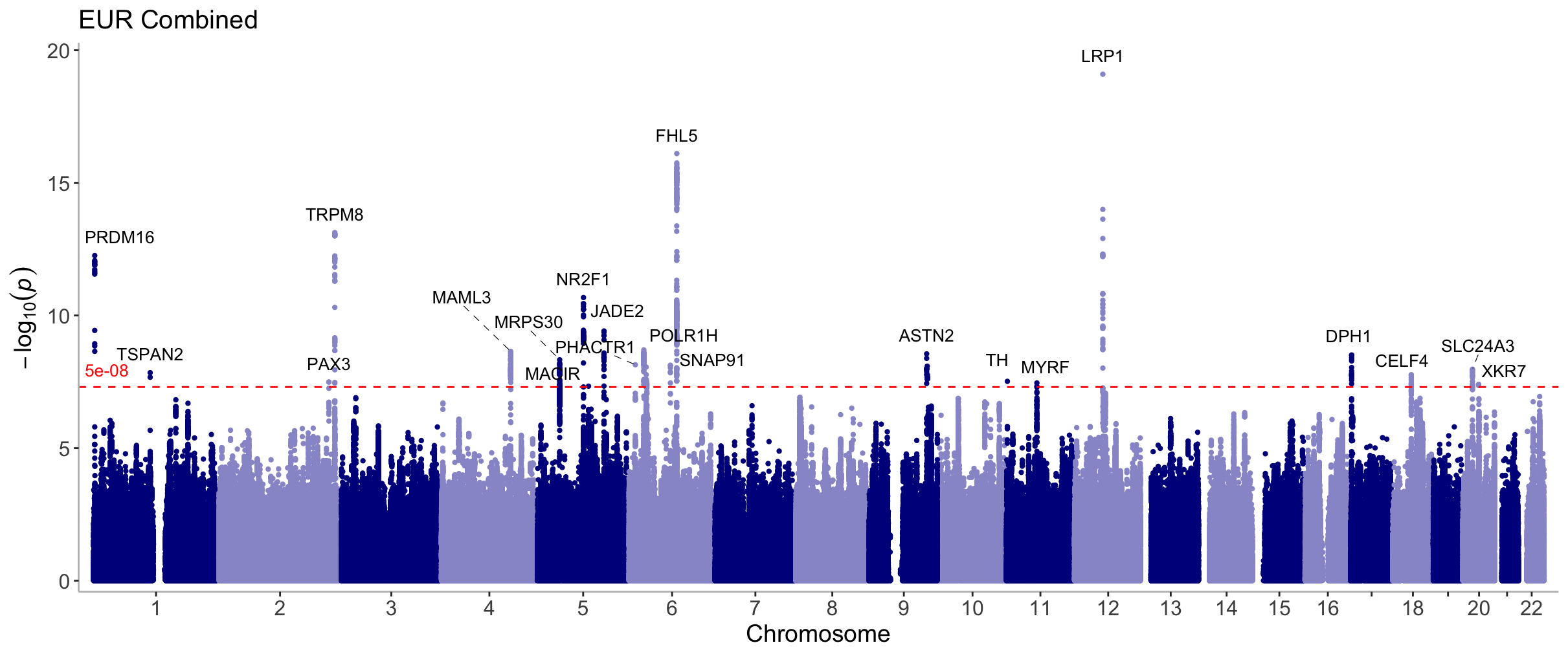
**

**Supplementary Figure 3d.** Genome-wide association study Manhattan plots of migraine for **AFR men** strata in MVP. The x-axis represents the chromosome position, and the y-axis represents the GWAS significance level (*p* on a -log_10_ scale). The dashed red line shows genome-wide significance at p>5x10^-8^.


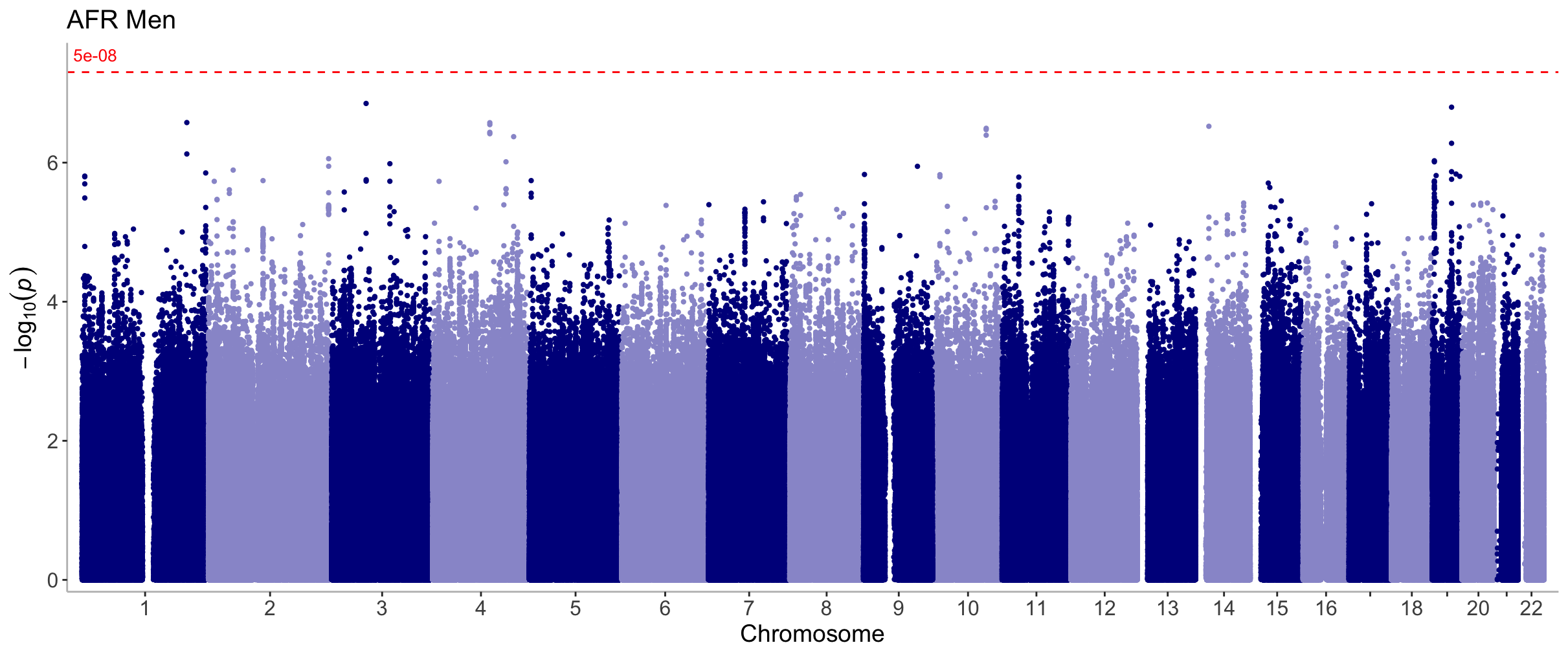


**Supplementary Figure 3e.** Genome-wide association study Manhattan plots of migraine for **AFR women** strata in MVP. The x-axis represents the chromosome position, and the y-axis represents the GWAS significance level (*p* on a -log_10_ scale). The dashed red line shows genome-wide significance at p>5x10^-8^.


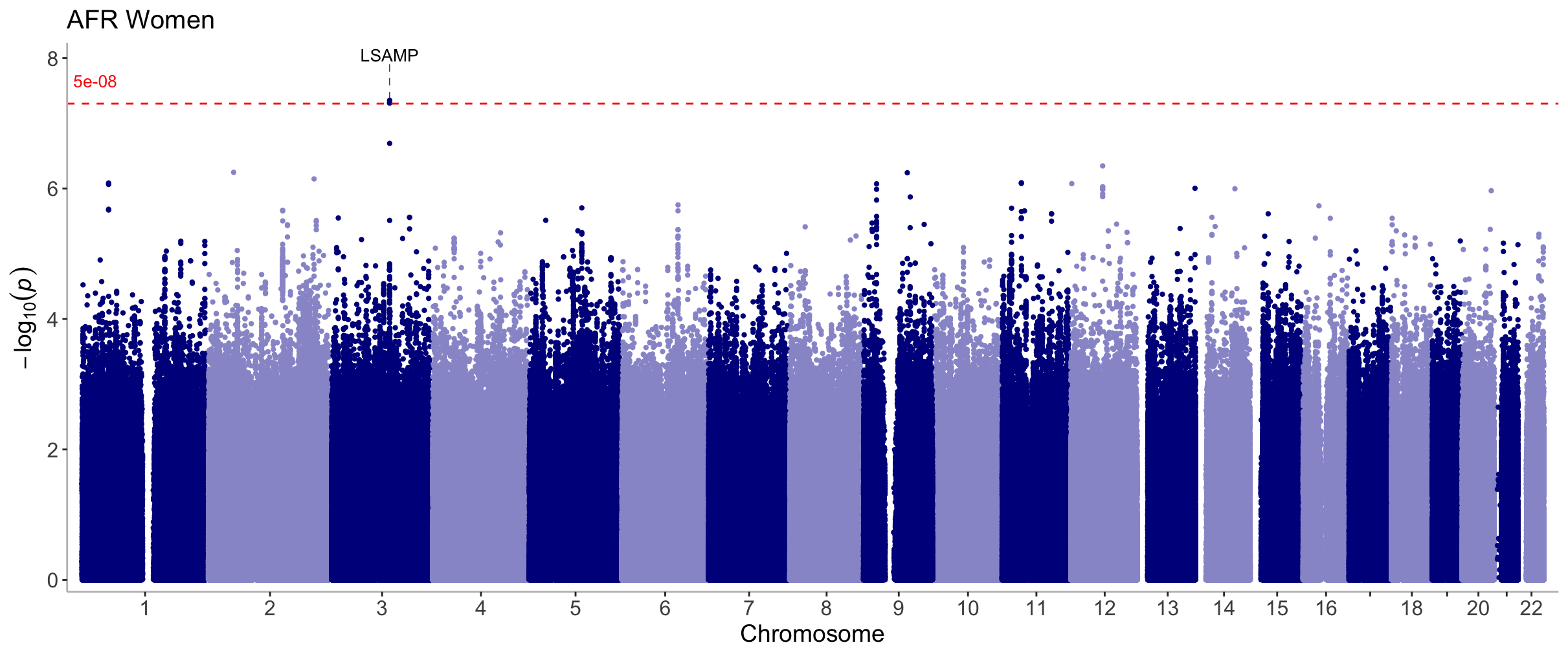


**Supplementary Figure 3f.** Genome-wide association study Manhattan plots of migraine for **AFR** combined (men and women) strata in MVP. The x-axis represents the chromosome position, and the y-axis represents the GWAS significance level (*p* on a -log_10_ scale). The dashed red line shows genome-wide significance at p>5x10^-8^.

**
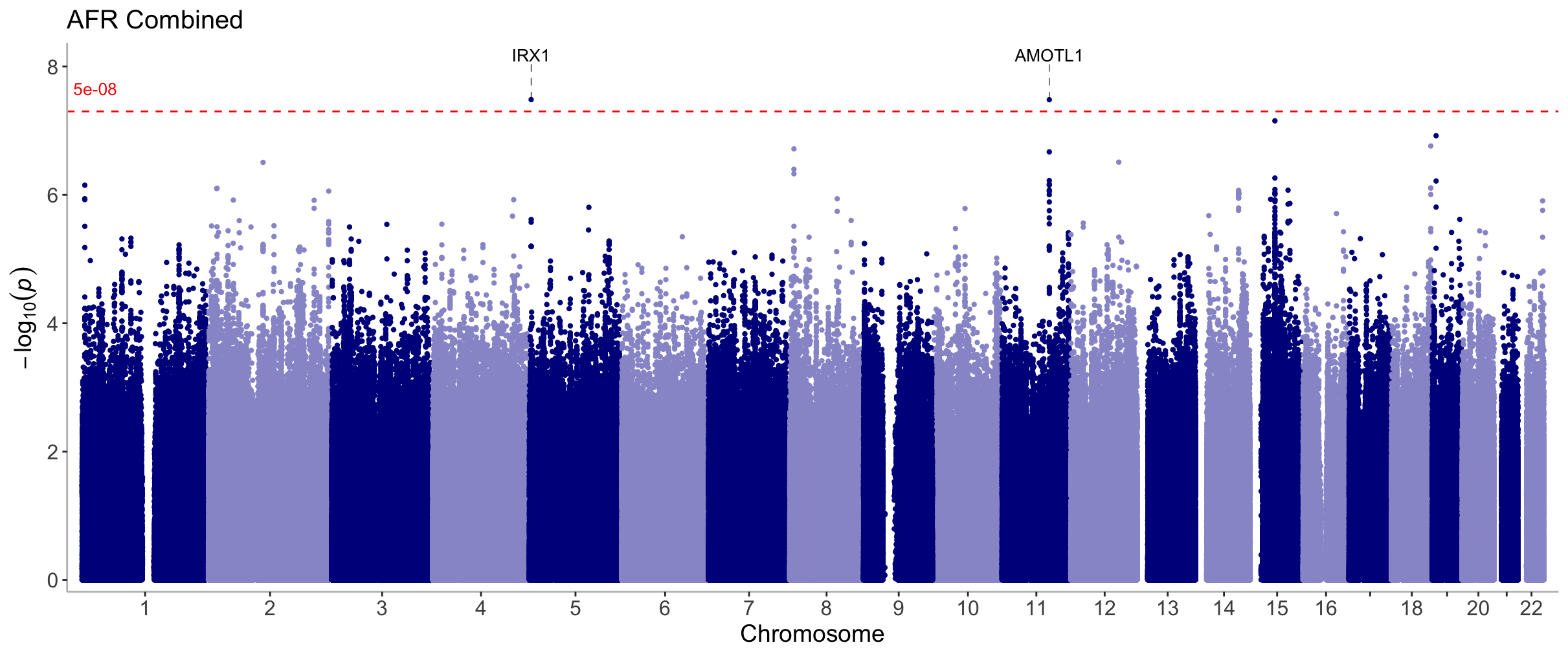
**

**Supplementary Figure 3g.** Genome-wide association study Manhattan plots of migraine for **HIS men** strata in MVP. The x-axis represents the chromosome position, and the y-axis represents the GWAS significance level (*p* on a -log_10_ scale). The dashed red line shows genome-wide significance at p>5x10^-8^.


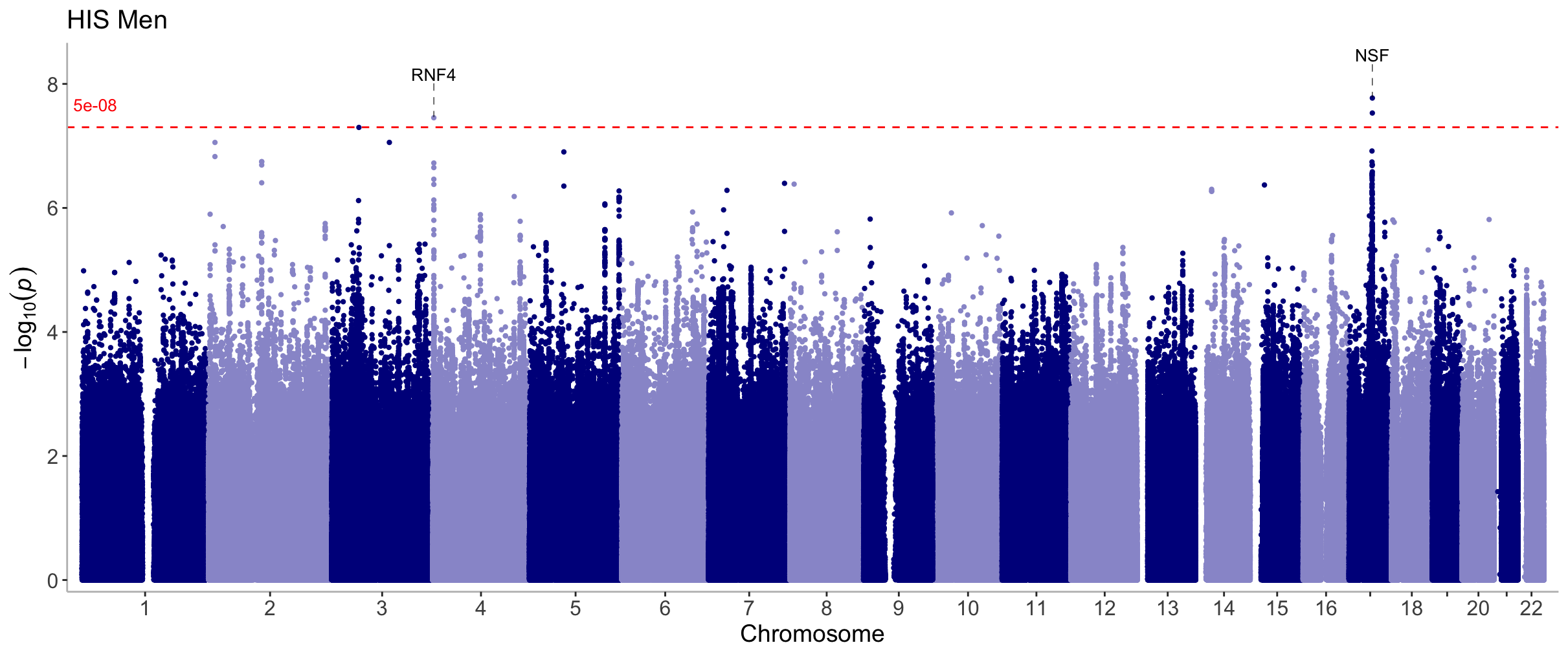


**Supplementary Figure 3h.** Genome-wide association study Manhattan plots of migraine for **HIS women** strata in MVP. The x-axis represents the chromosome position, and the y-axis represents the GWAS significance level (*p* on a -log_10_ scale). The dashed red line shows genome-wide significance at p>5x10^-8^.


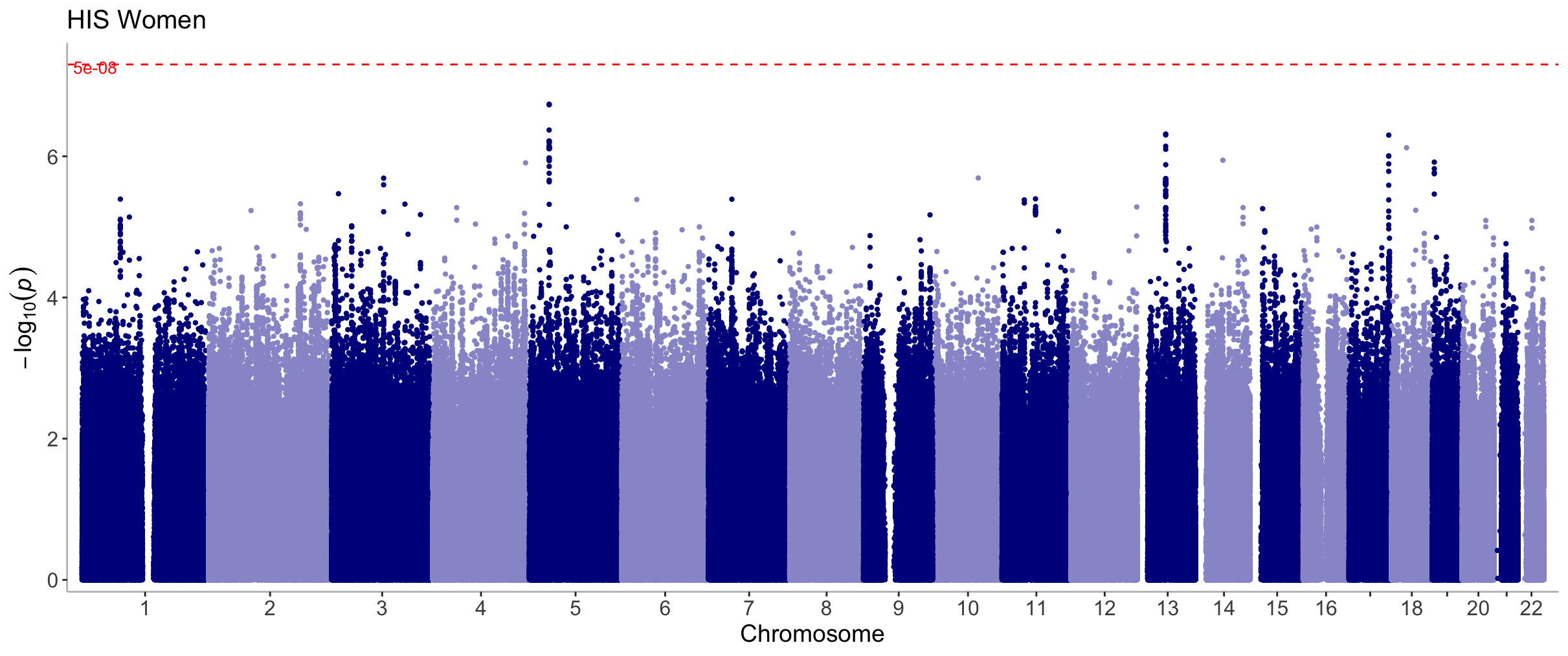
**Supplementary Figure 3i.** Genome-wide association study Manhattan plots of migraine for **HIS** **combined** (men and women) strata in MVP. The x-axis represents the chromosome position, and the y-axis represents the GWAS significance level (*p* on a -log_10_ scale). The dashed red line shows genome-wide significance at p>5x10^-8^.


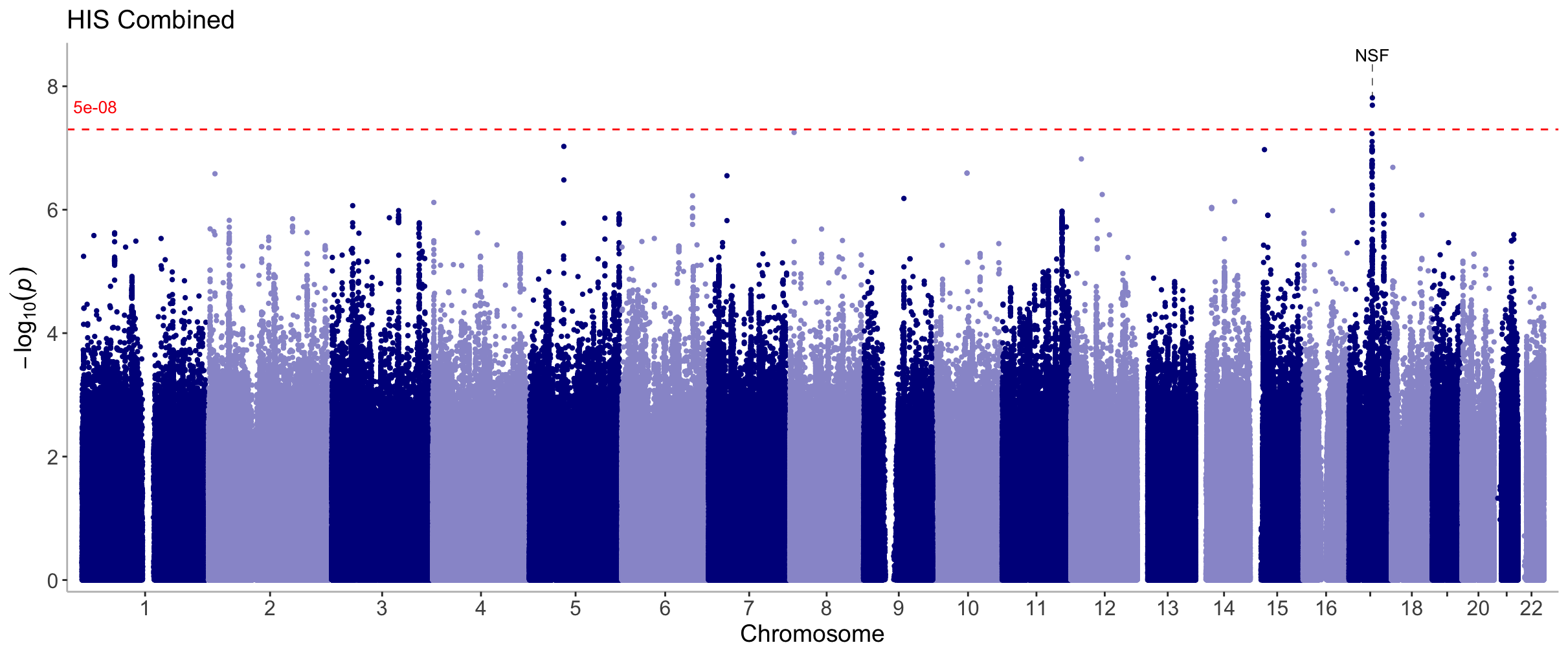


**Supplementary Figure 3j.** Genome-wide association study Manhattan plots of migraine for **META men** strata in MVP. The x-axis represents the chromosome position, and the y-axis represents the GWAS significance level (*p* on a -log_10_ scale). The dashed red line shows genome-wide significance at p>5x10^-8^.


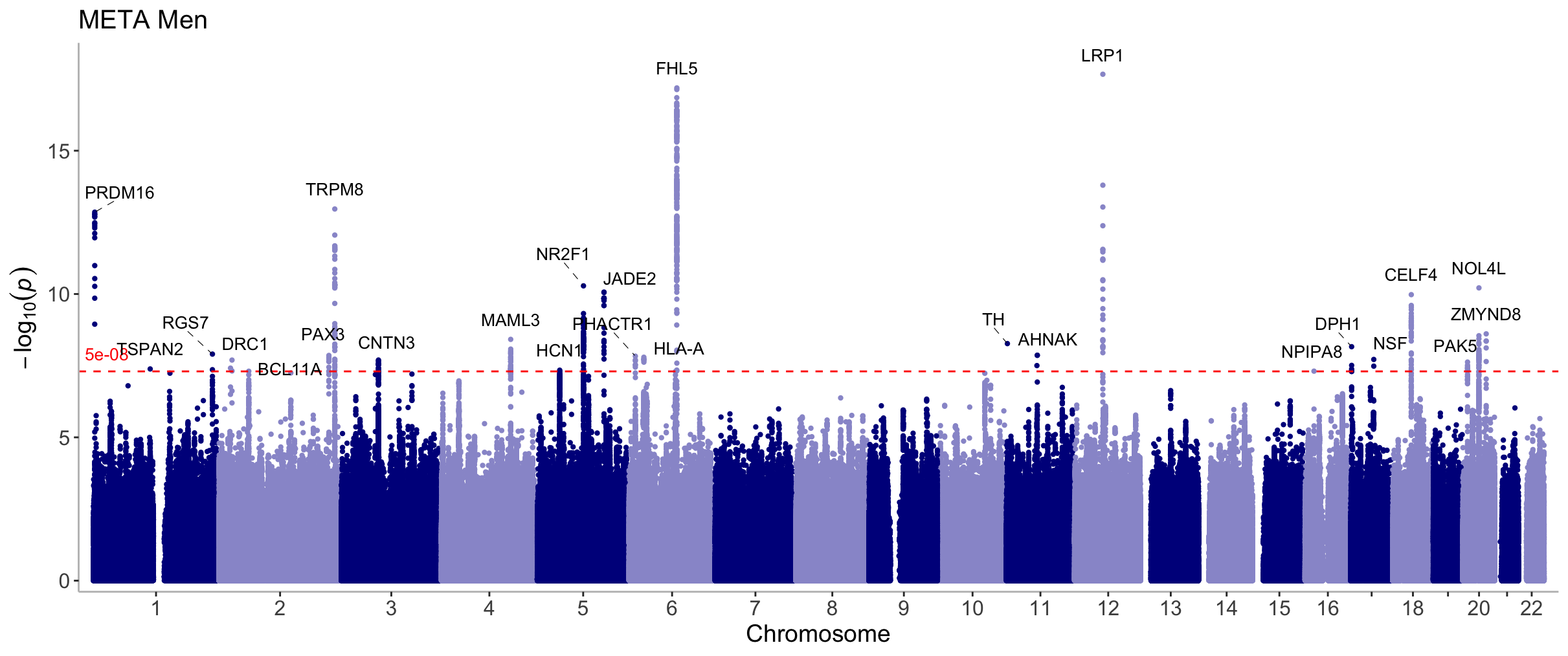


**Supplementary Figure 3k.** Genome-wide association study Manhattan plots of migraine for **META women** strata in MVP. The x-axis represents the chromosome position, and the y-axis represents the GWAS significance level (*p* on a -log_10_ scale). The dashed red line shows genome-wide significance at p>5x10^-8^.


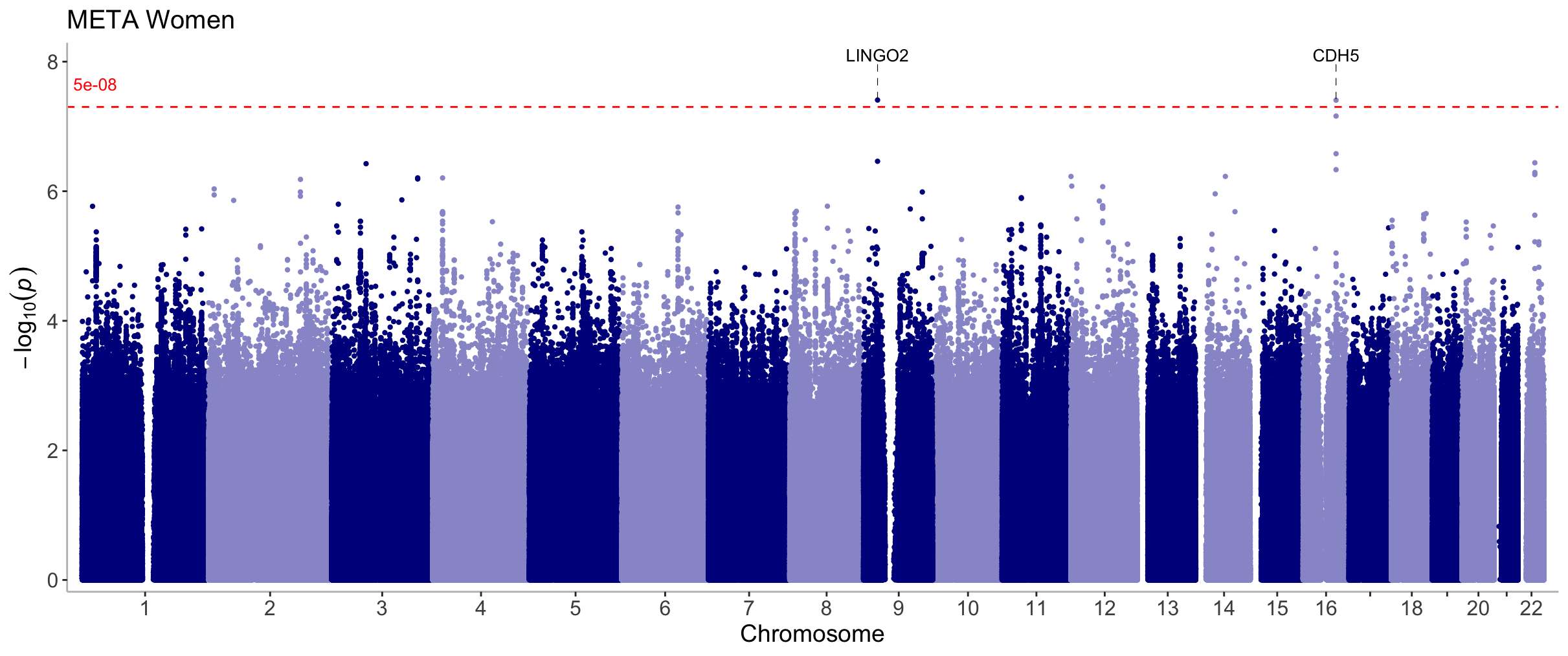


**Supplementary Figure 3l.** Genome-wide association study Manhattan plots of migraine for **META** **combined** (men and women) strata in MVP. The x-axis represents the chromosome position, and the y-axis represents the GWAS significance level (*p* on a -log_10_ scale). The dashed red line shows genome-wide significance at p>5x10^-8^.


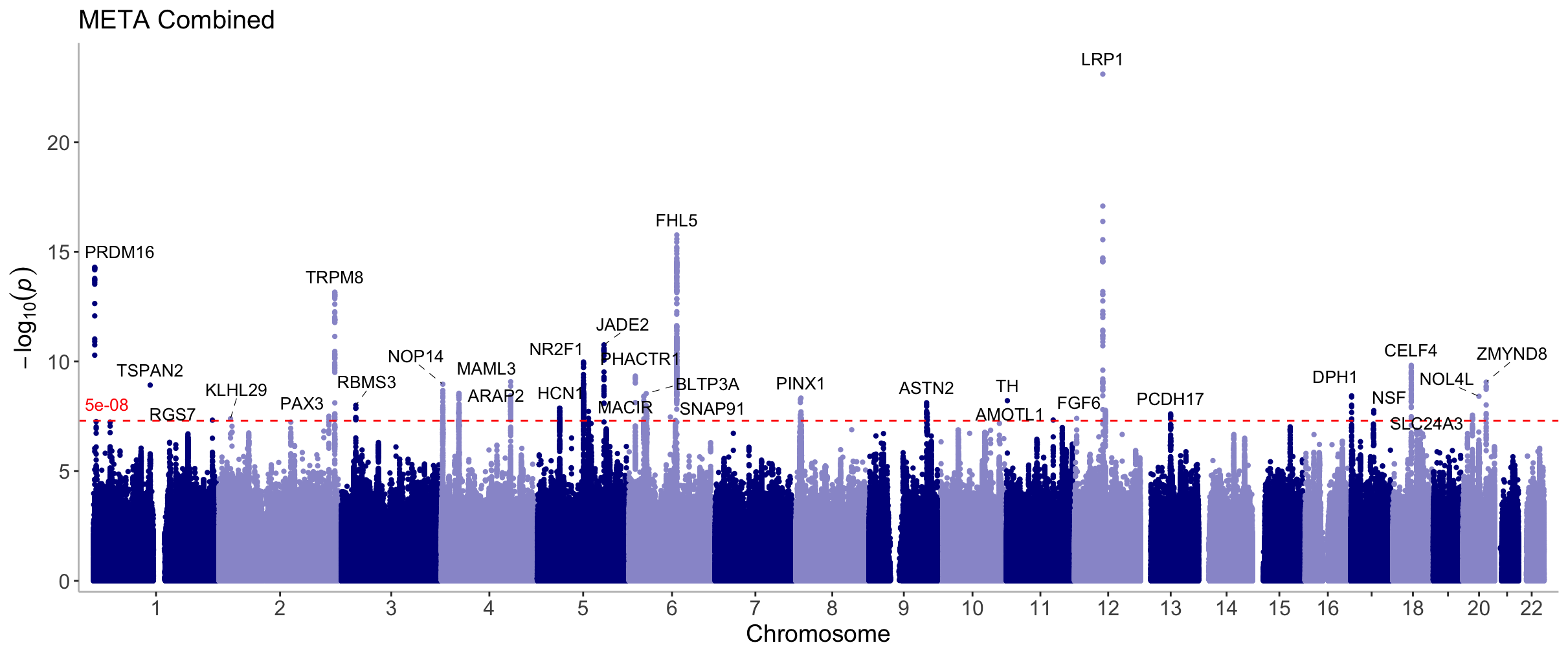


**Supplementary Figure 4 (a-kk). Regional association plots for novel replicated and unreplicated MVP migraine loci.**  The x-axis indicates the chromosomal position, and significance (-log10 p) values for each single nucleotide polymorphism (SNP) are indicated on the y-axis. The lead SNP appears in purple, while other SNPs in the locus are color-coded based on their linkage disequilibrium (r2) with the lead SNP. Annotated genes and biotypes are presented in the panel below. Only SNPs which are in LD of any of significant independent lead SNPs are displayed in the CADD plot. Of those SNPs, SNPs which did not used for mapping (SNPs that were filtered by user defined parameters) are colored grey.

Presented are loci that were novel replicated or novel unreplicated to this study. META_C loci (21) are presented in panels a-x; trans-ancestry metanalysis of Men (META_men) loci (7) in panels y-ee, trans-ancestry metanalysis of women (META_women) loci (2) in panels ff-gg, EUR mena and women combined (EUR_C) locus in hh, AFR men and women combined (AFR_C) locus in ii, AFR women (AFR_women) locus in jj, and HIS men (HIS_men) locus in kk. Loci presented in main manuscript Figure 1b correspond to the Supplementary Table 6 and the locus plots below. When the SNP was GWS in more than one stratum, it is presented for the Meta Combined (META_C) results . For example, global locus 1 was GWS as META_C_5 with lead SNP rs6714463, META_MEN_7 with rs4674625, EUR_C_3 with rs62178704, and EUR_MEN_2 with rs17394072 (Supplementary Table 6). The locus is presented from the META_C results in panel c below.

| 1. Meta Combined, Locus 3 (global_1). Lead SNP: rs7522181. |
| --- |
| 1. Meta Combined, Locus 4 (global_26). Lead SNP: rs13023745.    |
| 1. Meta Combined, Locus 5 (global_1). Lead SNP: rs6714463. |
| 1. Meta Combined, Locus 7. Lead SNP: rs9876003. |
| 1. Meta Combined, Locus 8. Lead SNP: rs2488813. |
| 1. Meta Combined, Locus 10 (global_3). Lead SNP: rs72712556. |
| 1. Meta Combined, Locus 11 (global_4). Lead SNP: rs62369897. |
| 1. Meta Combined, Locus 13 (global_15). Lead SNP: rs253754. |
| 1. Meta Combined, Locus 14 (global_6). Lead SNP: rs329117. |
| 1. Meta Combined, Locus 16 (global_17). Lead SNP: rs9469913. |
| 1. Meta Combined, Locus 17 (global_18). Lead SNP: rs851005. |
| 1. Meta Combined, Locus 18 (global_19). Lead SNP: rs34529044. |
| 1. Meta Combined, Locus 19. Lead SNP: rs28642196. |
| 1. Meta Combined, Locus 21. Lead SNP: rs6601327. |
| 1. Meta Combined, Locus 22. Lead SNP: rs1897207. |
| 1. Meta Combined, Locus 24 (global_21). Lead SNP: rs6478314; rs10123941. |
| 1. Meta Combined, Locus 25 (global_22). Lead SNP: rs7482510. |
| 1. Meta Combined, Locus 26 (global_24). Lead SNP: rs73527819. |
| 1. Meta Combined, Locus 29. Lead SNP: rs34339271. |
| 1. Meta Combined, Locus 30. Lead SNP: rs3012863. |
| 1. Meta Combined, Locus 31 (global_10). Lead SNP: rs1873975; rs2641435. |
| 1. Meta Combined, Locus 32 (global_13). Lead SNP: rs12453054. |
| 1. Meta Combined, Locus 33 (global_11). Lead SNP: rs7242923. |
| 1. Meta Combined, Locus 35 (global_12). Lead SNP: rs4911242. |
| 1. Meta Men, Locus 5. Lead SNP: rs13007894. |
| 1. Meta Men, Locus 6. Lead SNP: rs1553933. |
| 1. Meta Men, Locus 9. Lead SNP: rs143362757. |
| 1. Meta Men, Locus 17. Lead SNP: rs509360. |
| 1. Meta Men, Locus 18. Lead SNP: rs75855515. |
| 1. Meta Men, Locus 20. Lead SNP: rs1428102. |
| 1. Meta Men, Locus 24. Lead SNP: rs2072952. |
| 1. Meta Women, Locus 1. Lead SNP: rs55938934. |
| 1. Meta Women, Locus 2. Lead SNP: rs1094921. |
| 1. EUR Combined, Locus 23. Lead SNP: rs112671224. |
| 1. AFR Combined, Locus 1. Lead SNP: rs114364083. |
| 1. AFR Women, Locus 1. Lead SNP: rs2864065. |
| 1. HIS Men, Locus 1. Lead SNP: rs846238. |

**Supplementary Figure 5. MAGMA tissue expression by strata.**

For each ancestry, the first panel shows the general tissue results, and the second panel shows the specific tissue results.

| 1. **EUR men**   **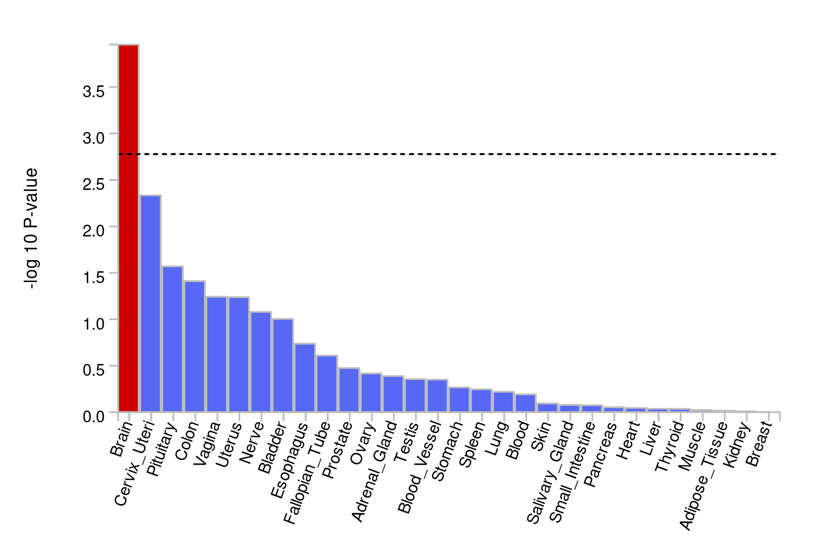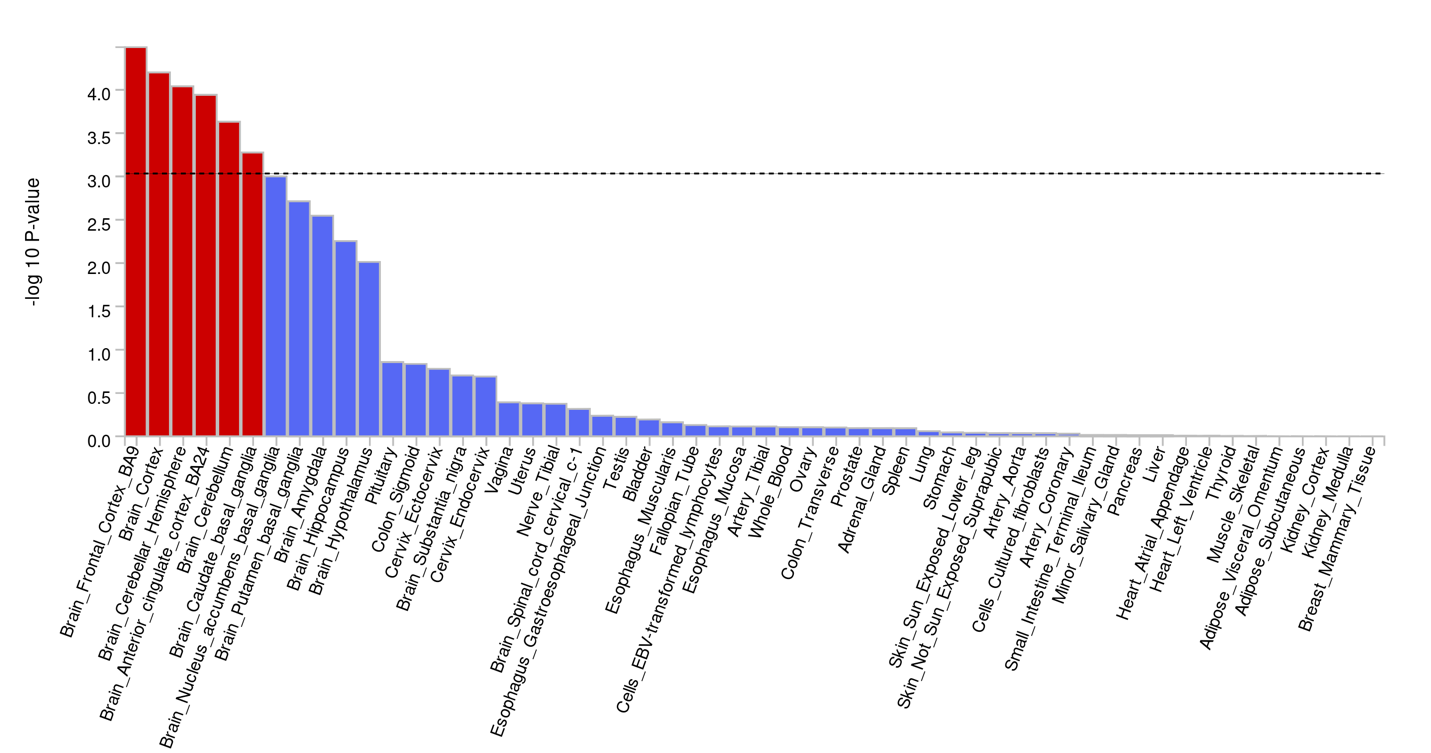** |
| --- |
| 1. **EUR women**   **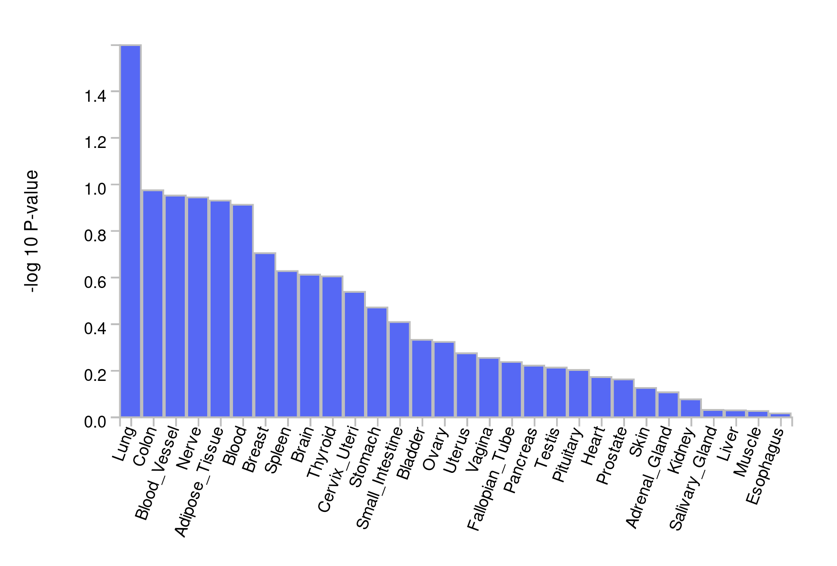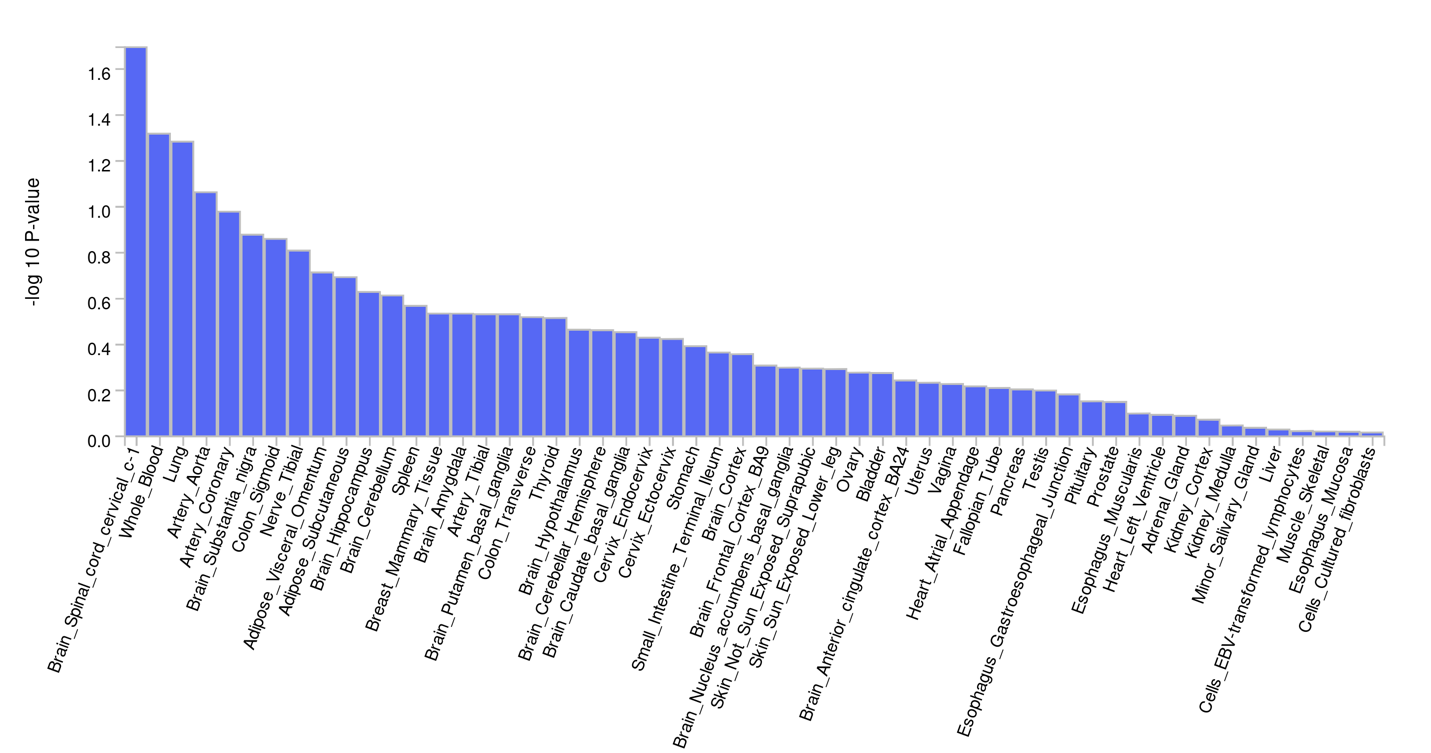** |
| 1. **EUR combined**   **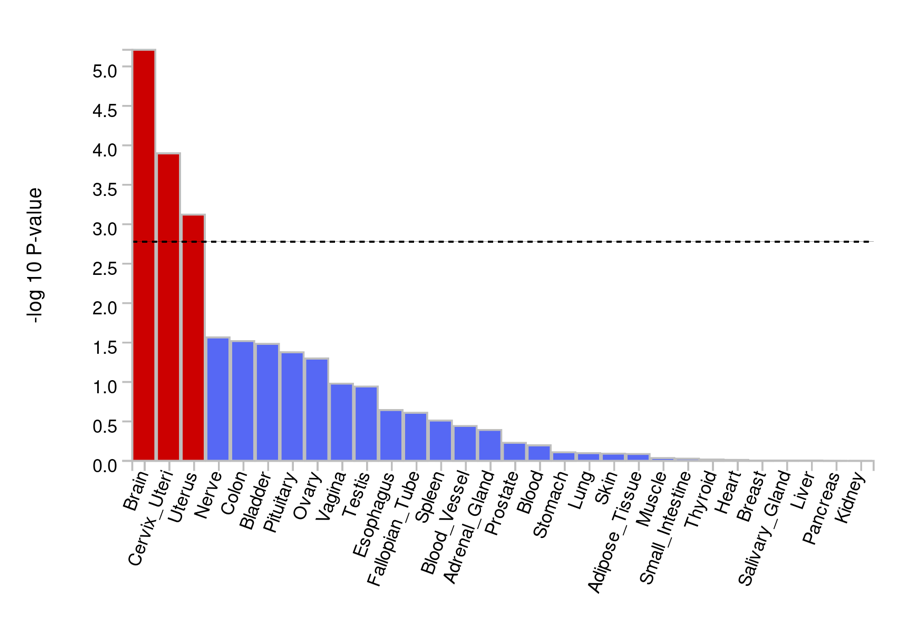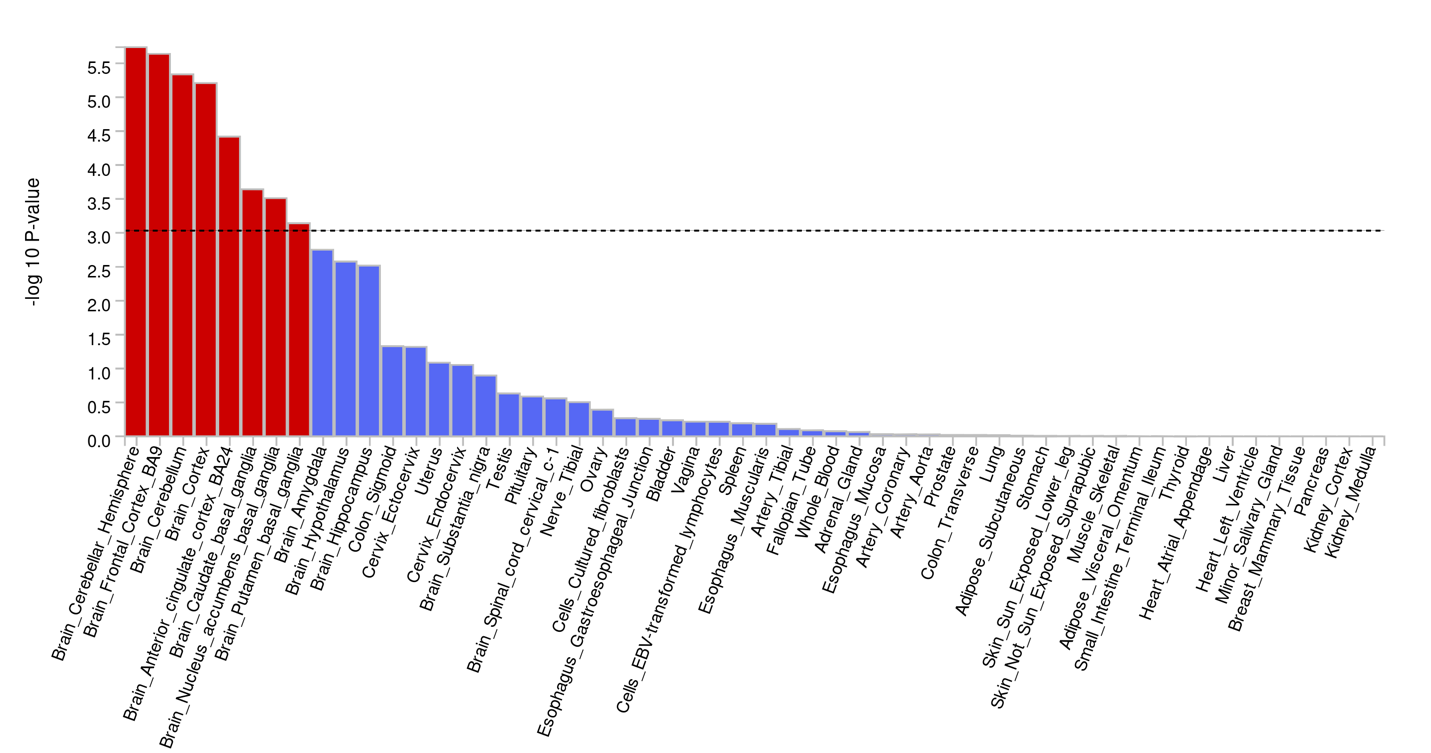** |
| 1. **AFR men**   **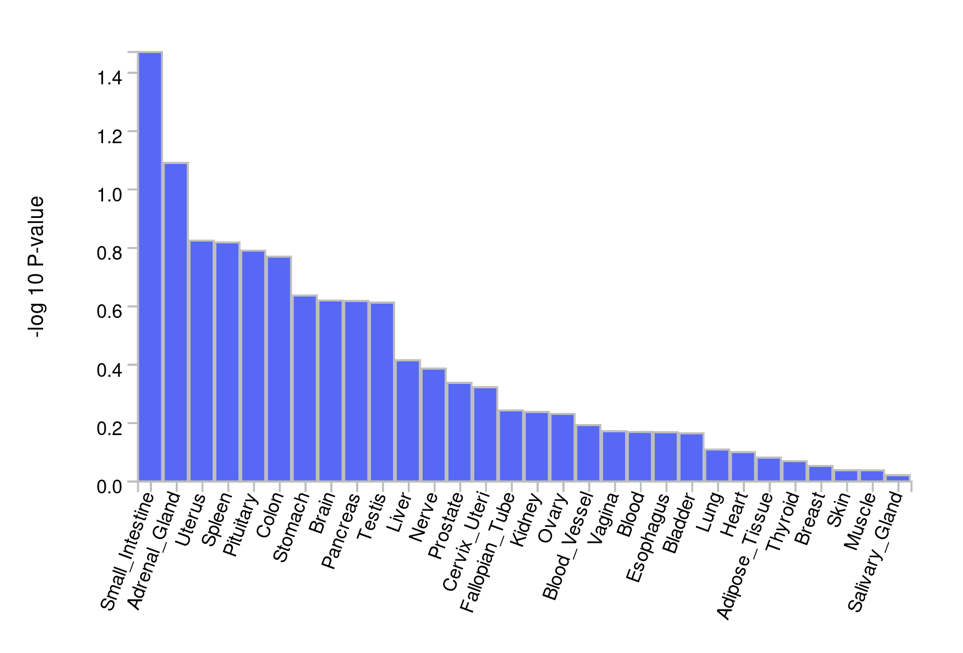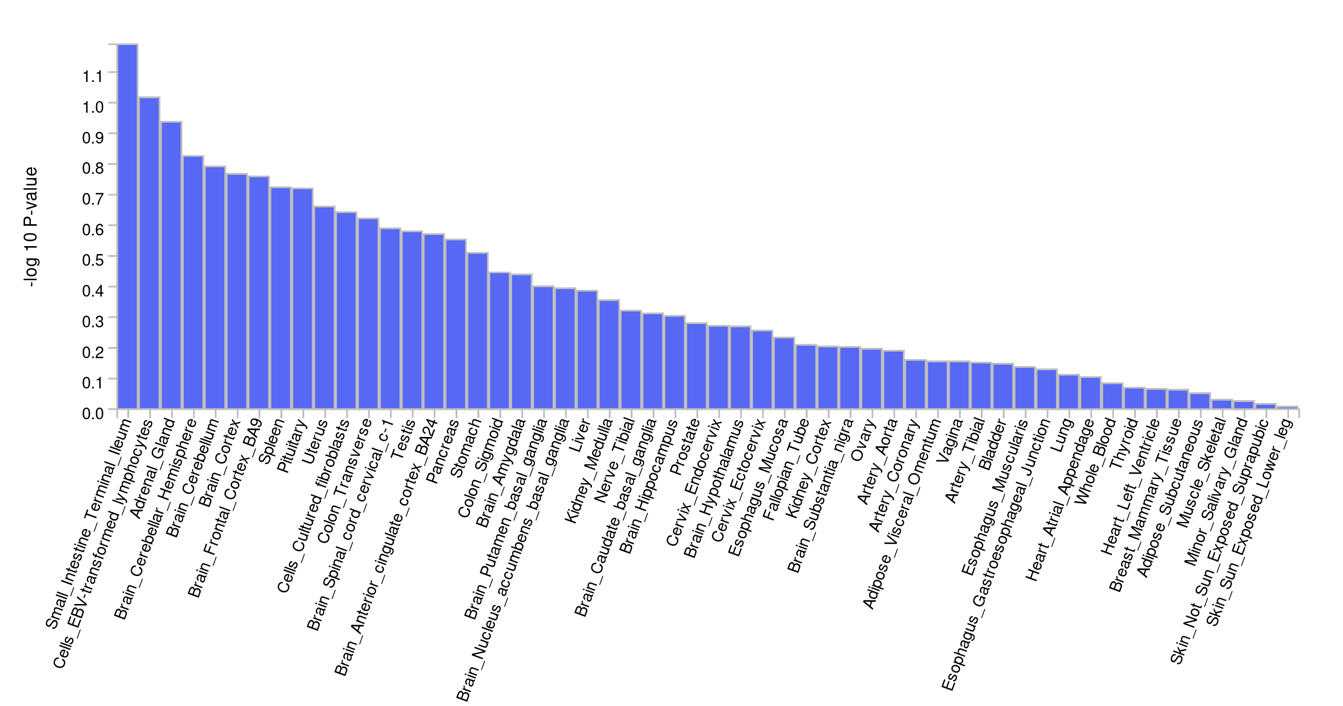** |
| 1. **AFR women**   **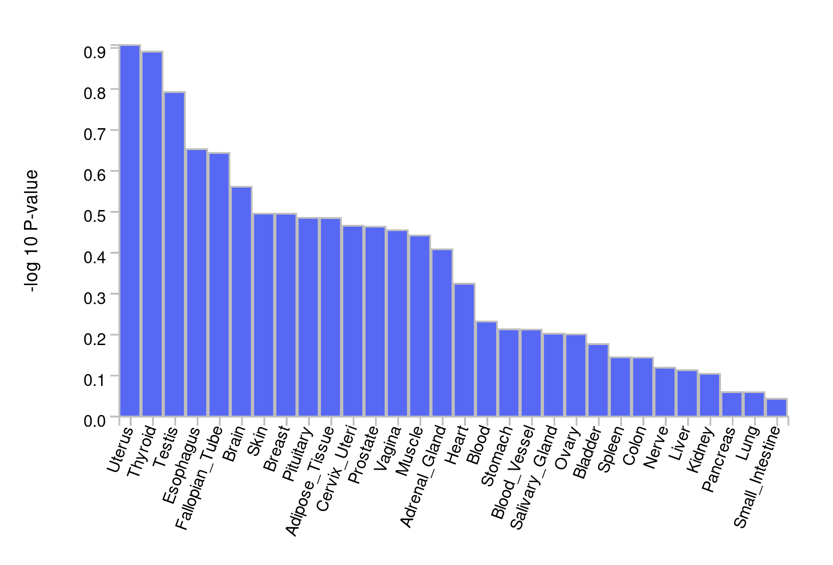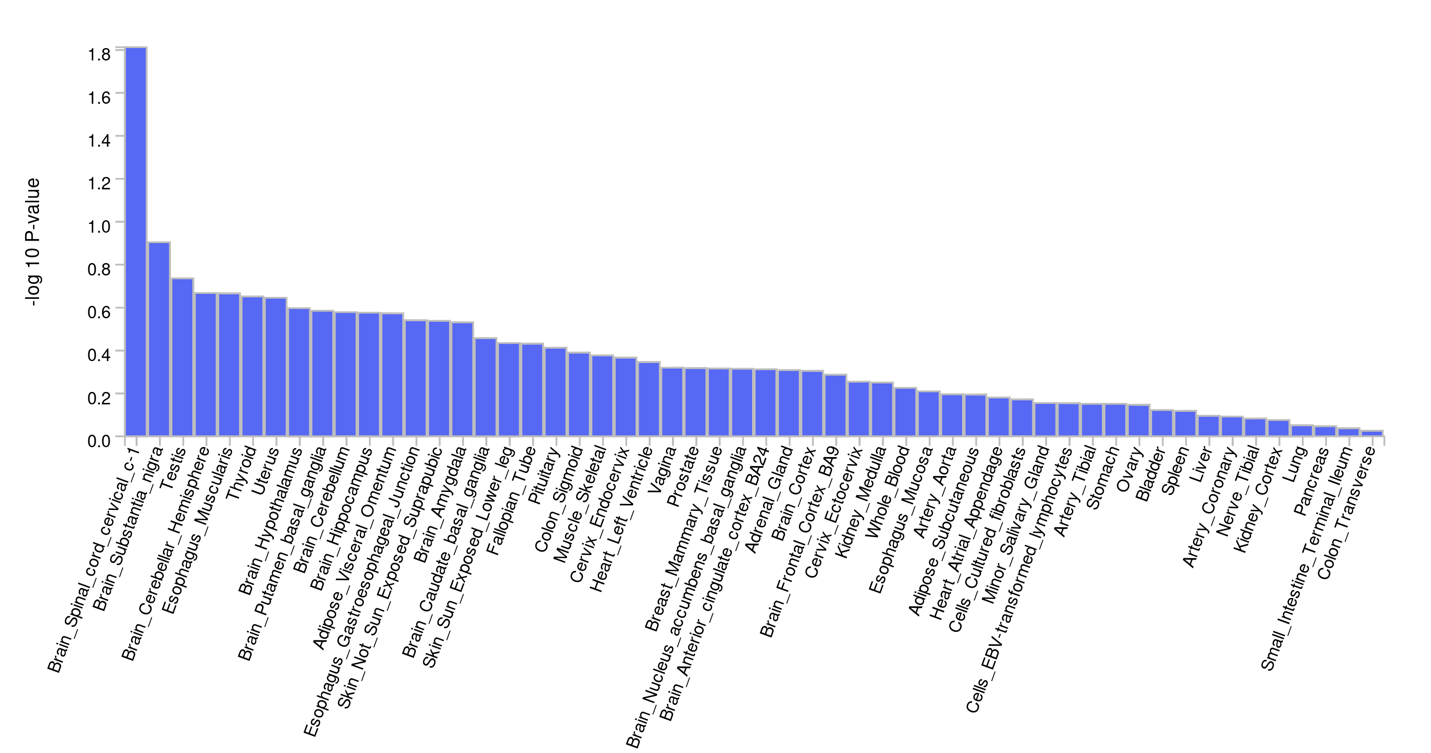** |
| 1. **AFR combined**   **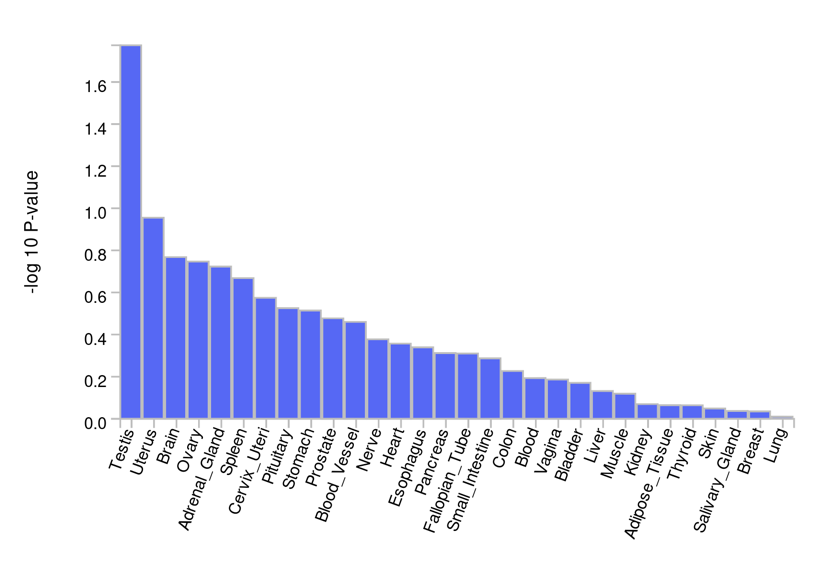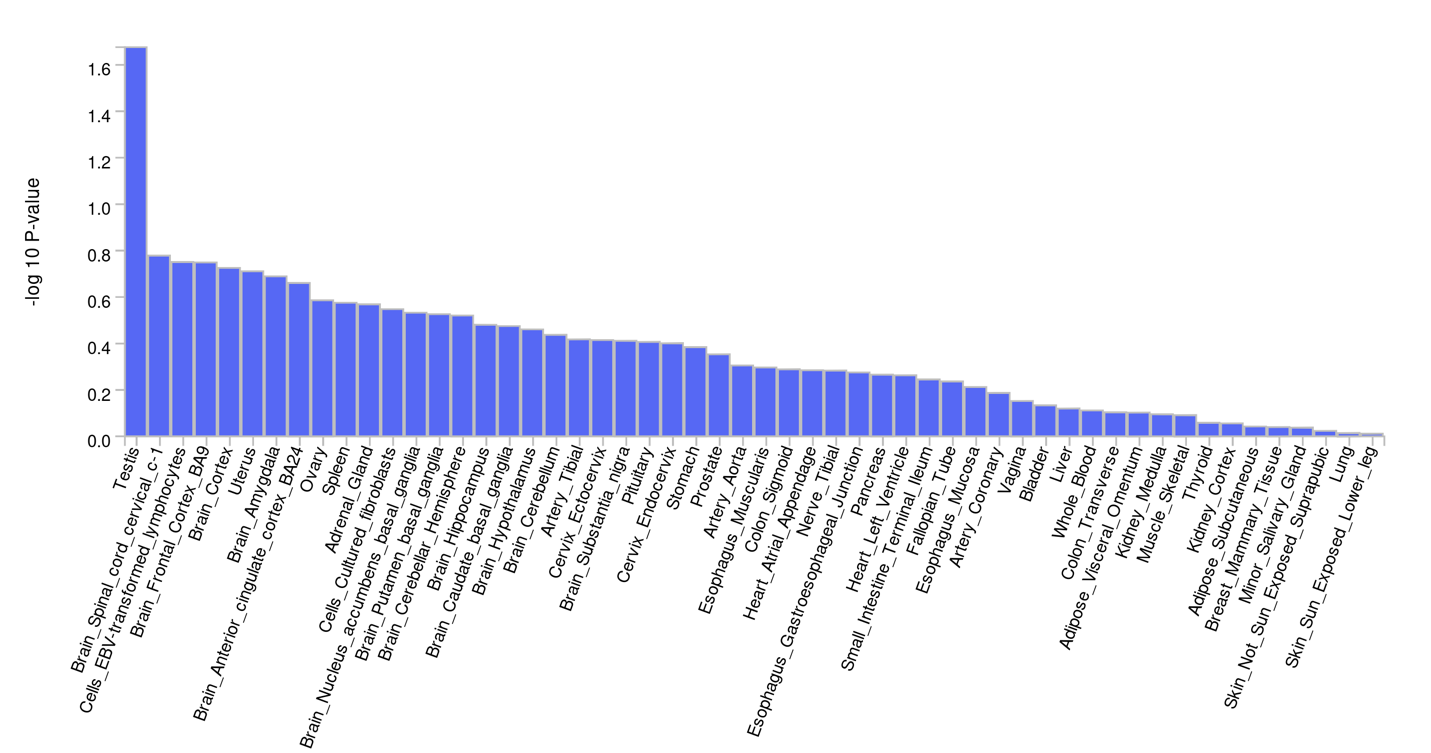** |
| 1. **HIS men**   **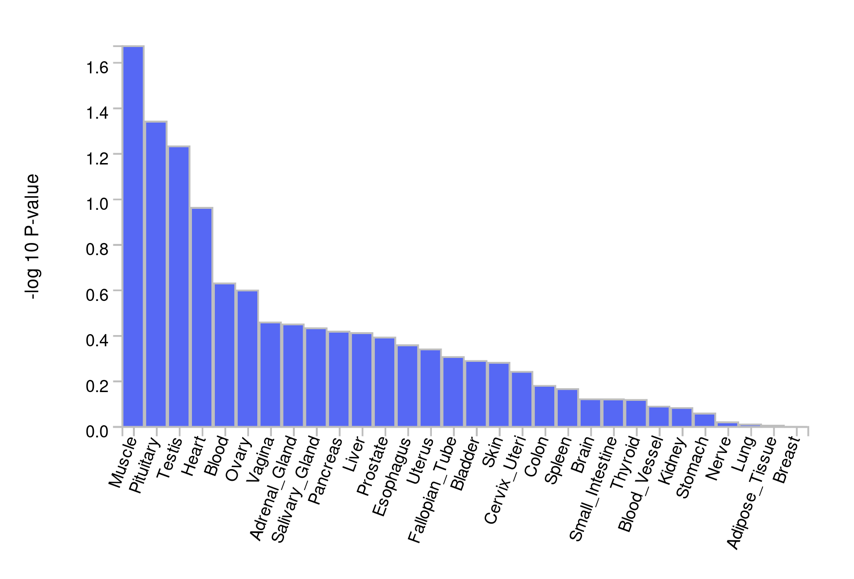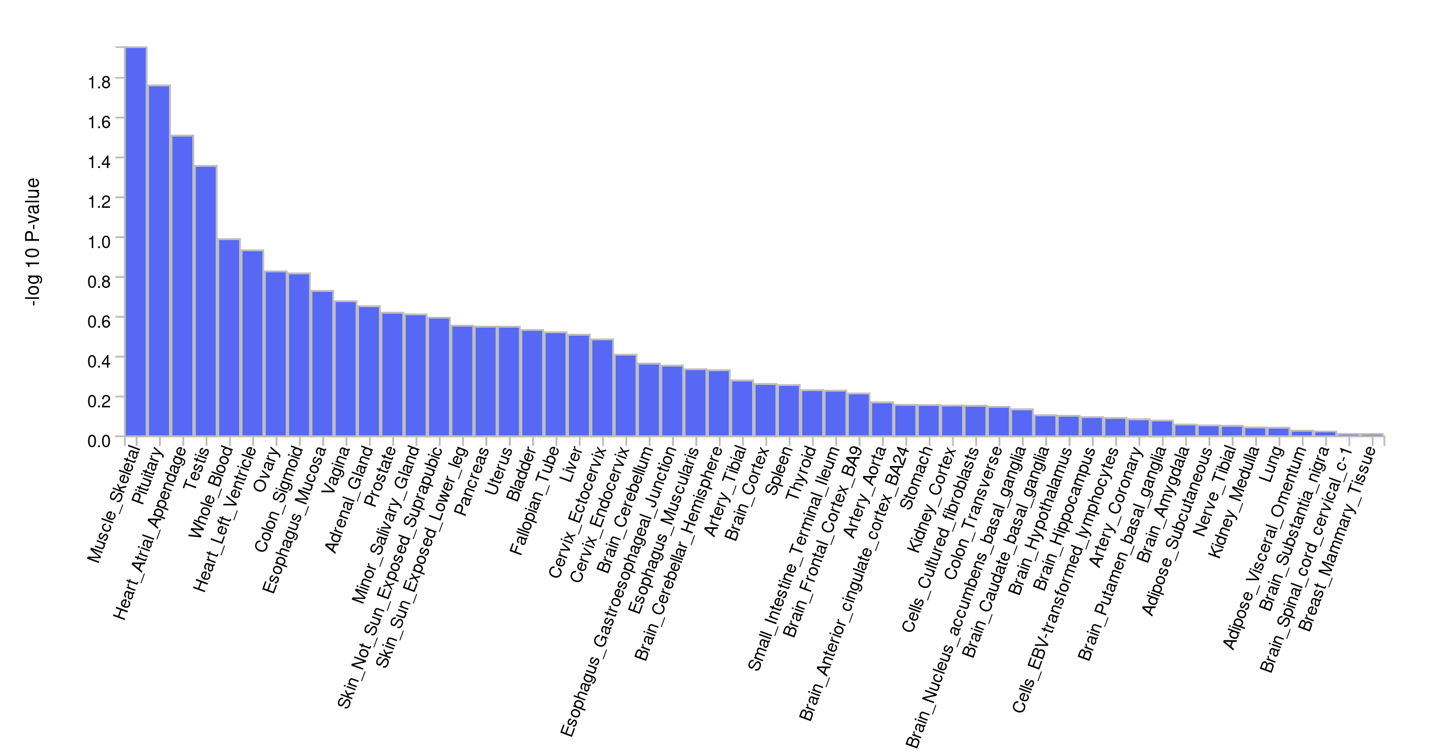** |
| 1. **HIS women**   **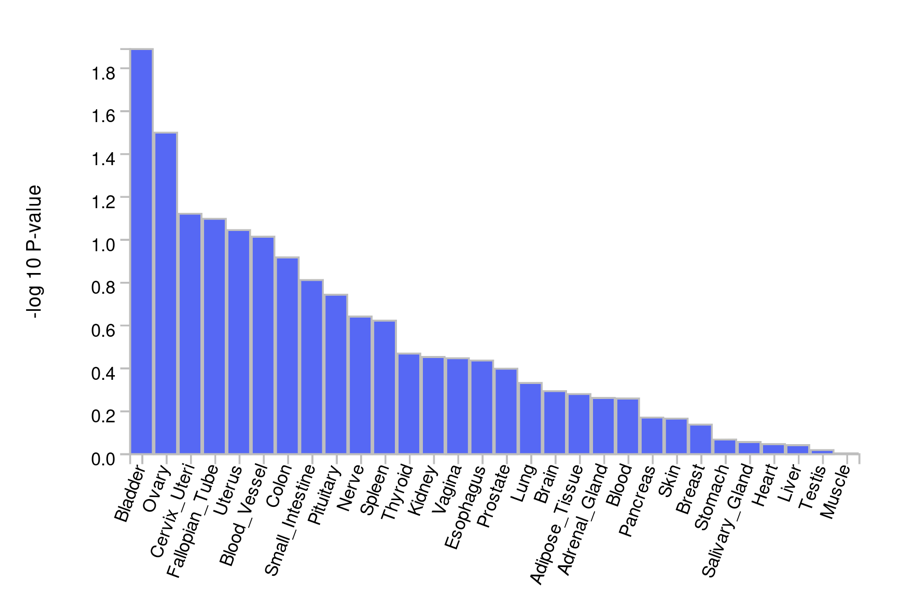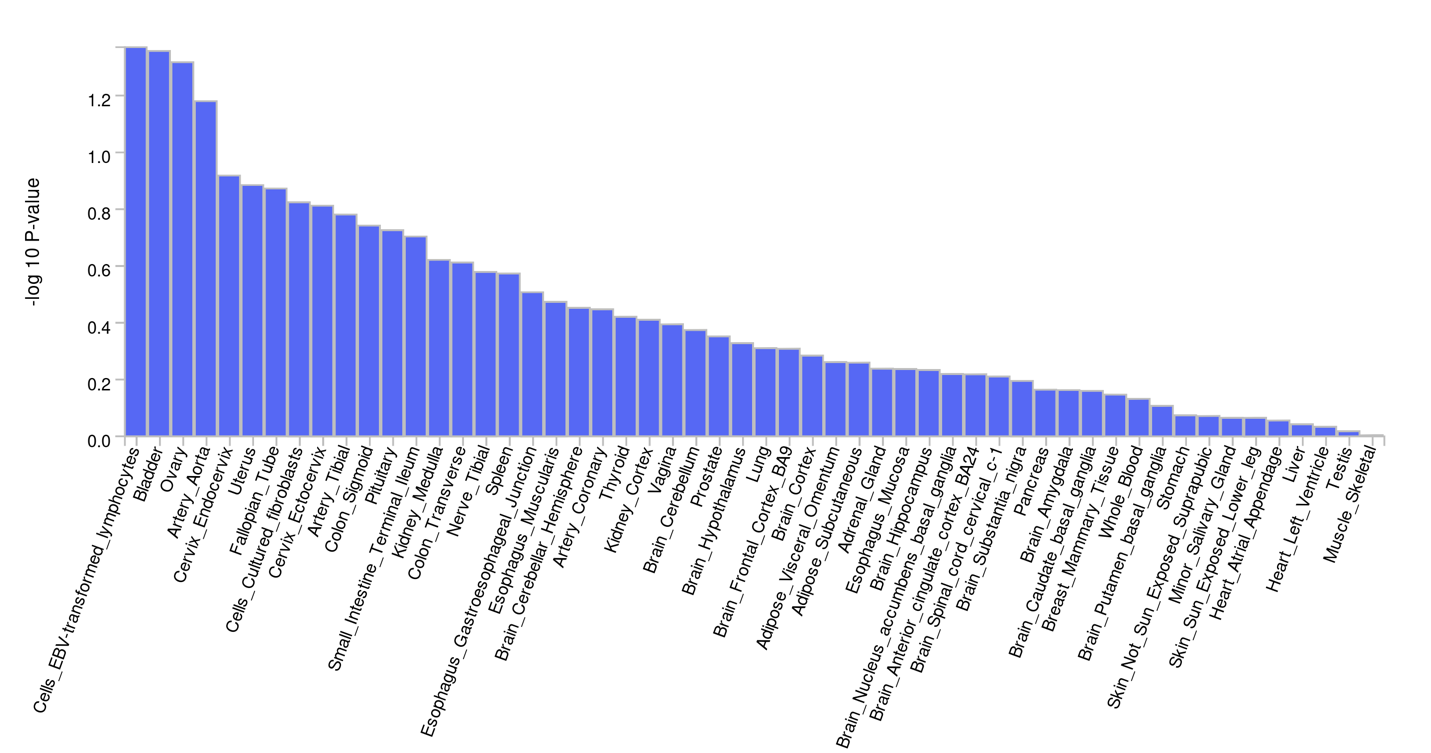** |
| 1. **HIS combined**   **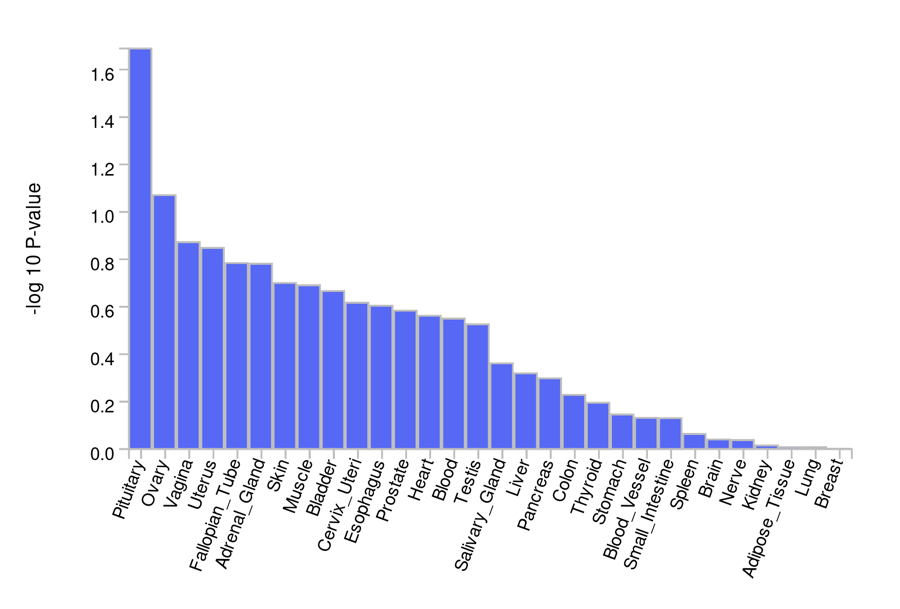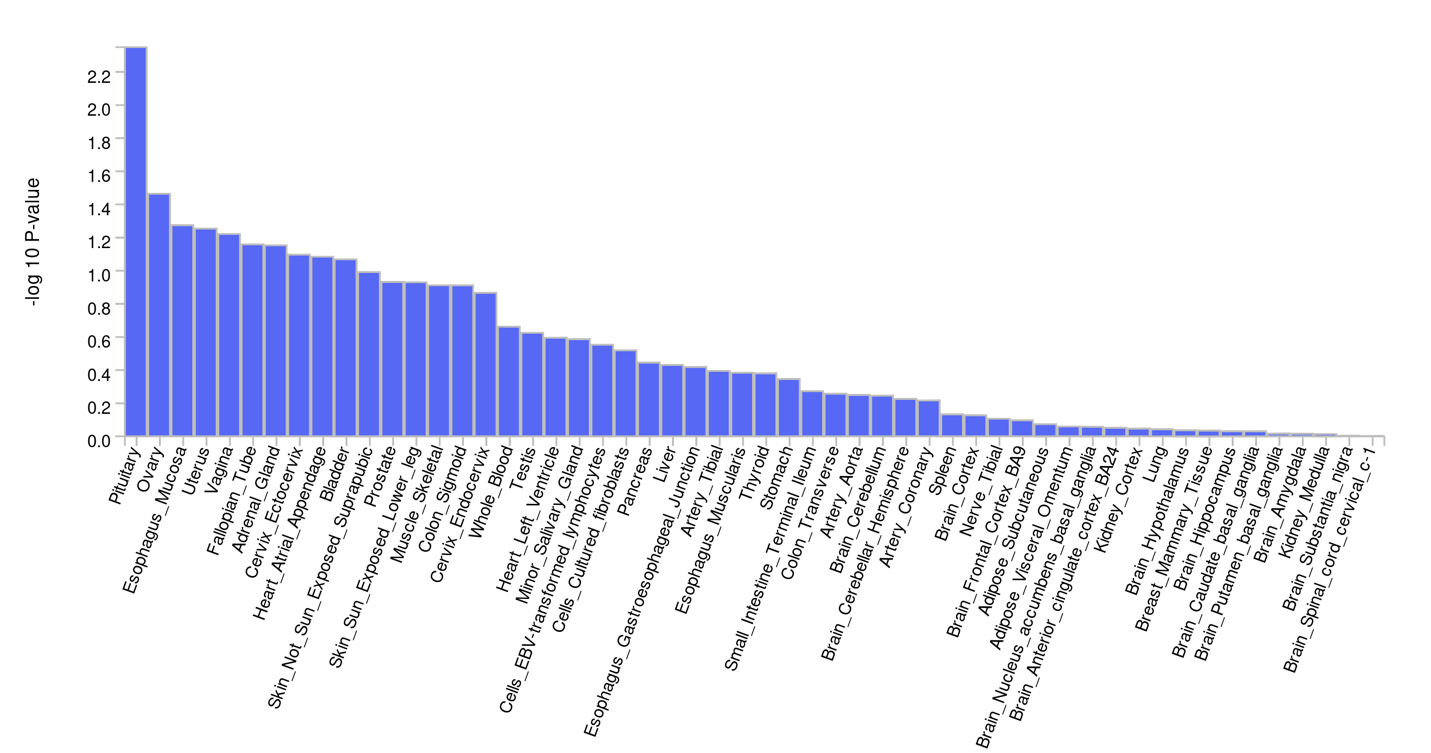** |
| 1. **META men**   **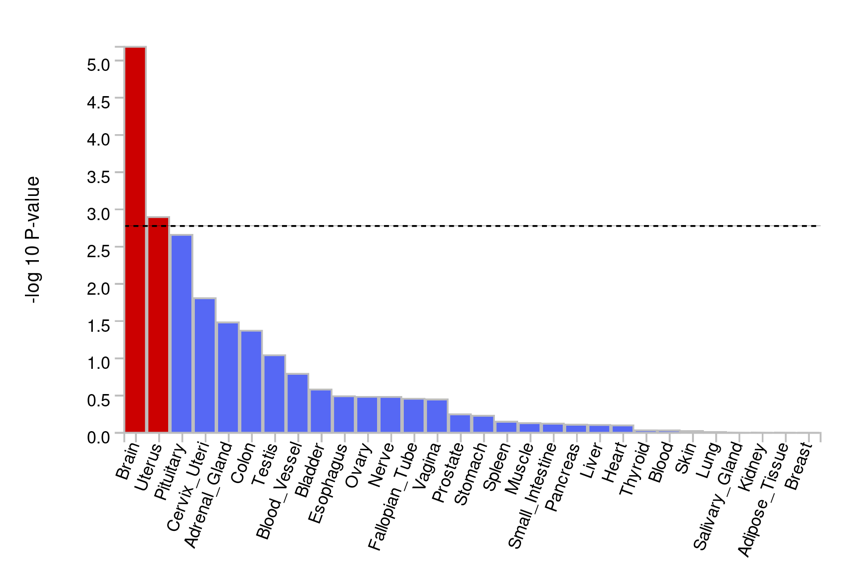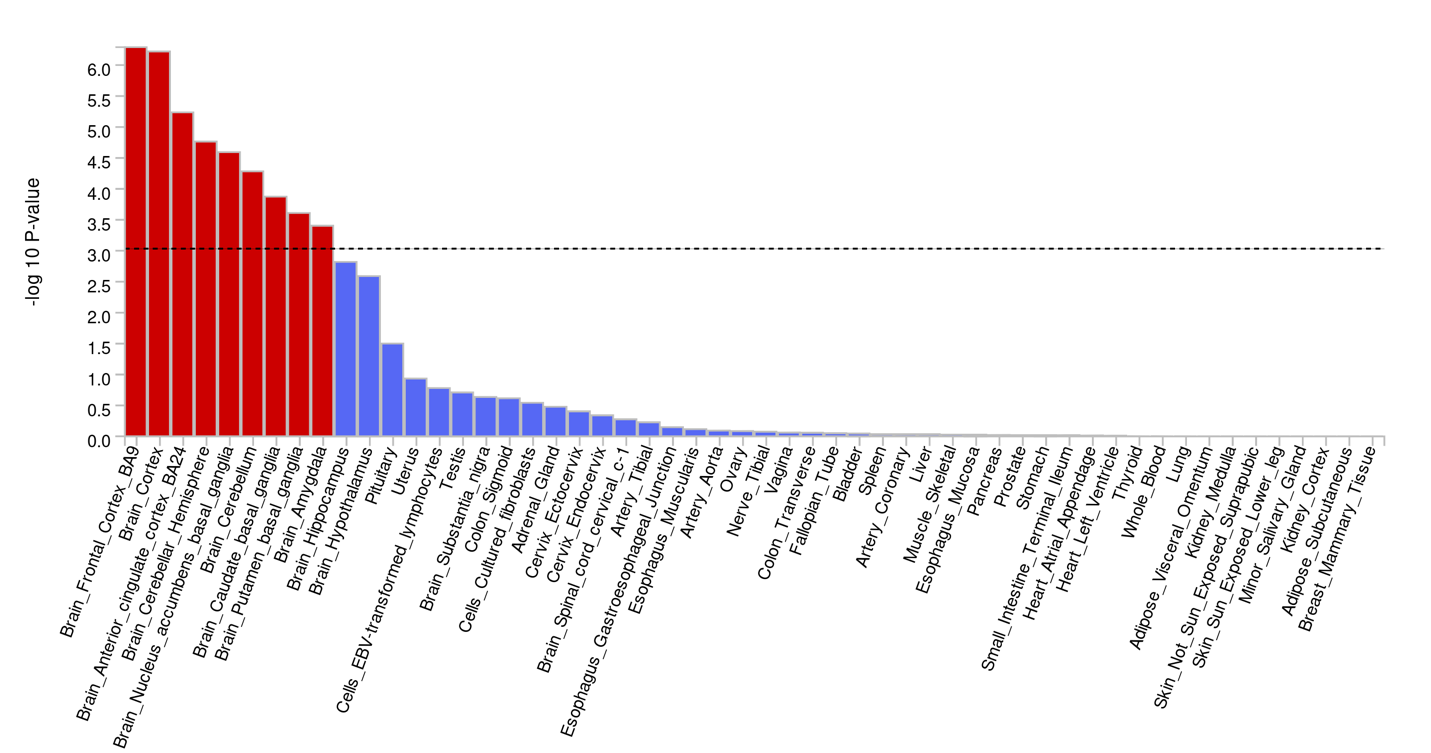** |
| 1. **META women**   **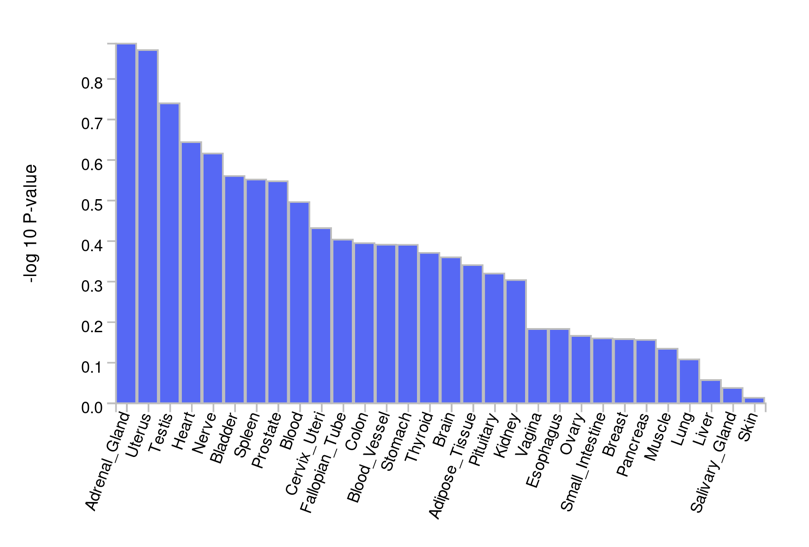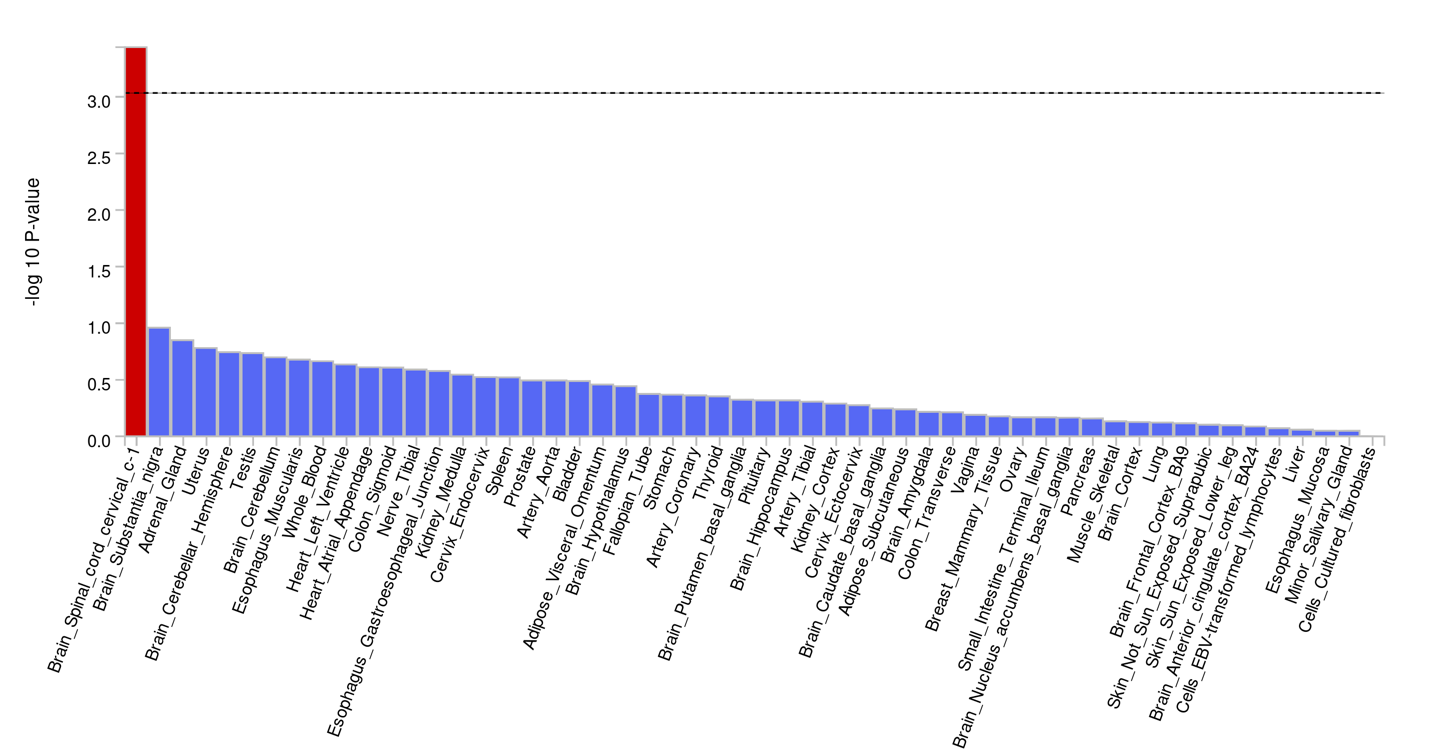** |
| 1. **META combined**   **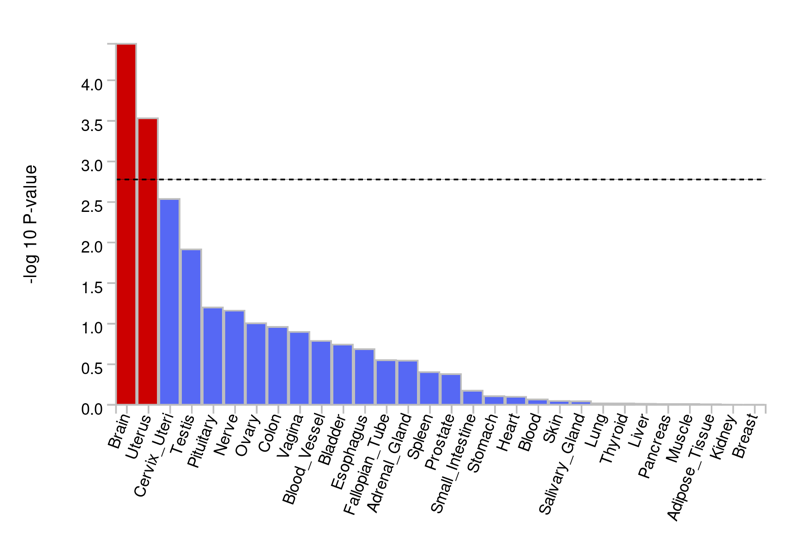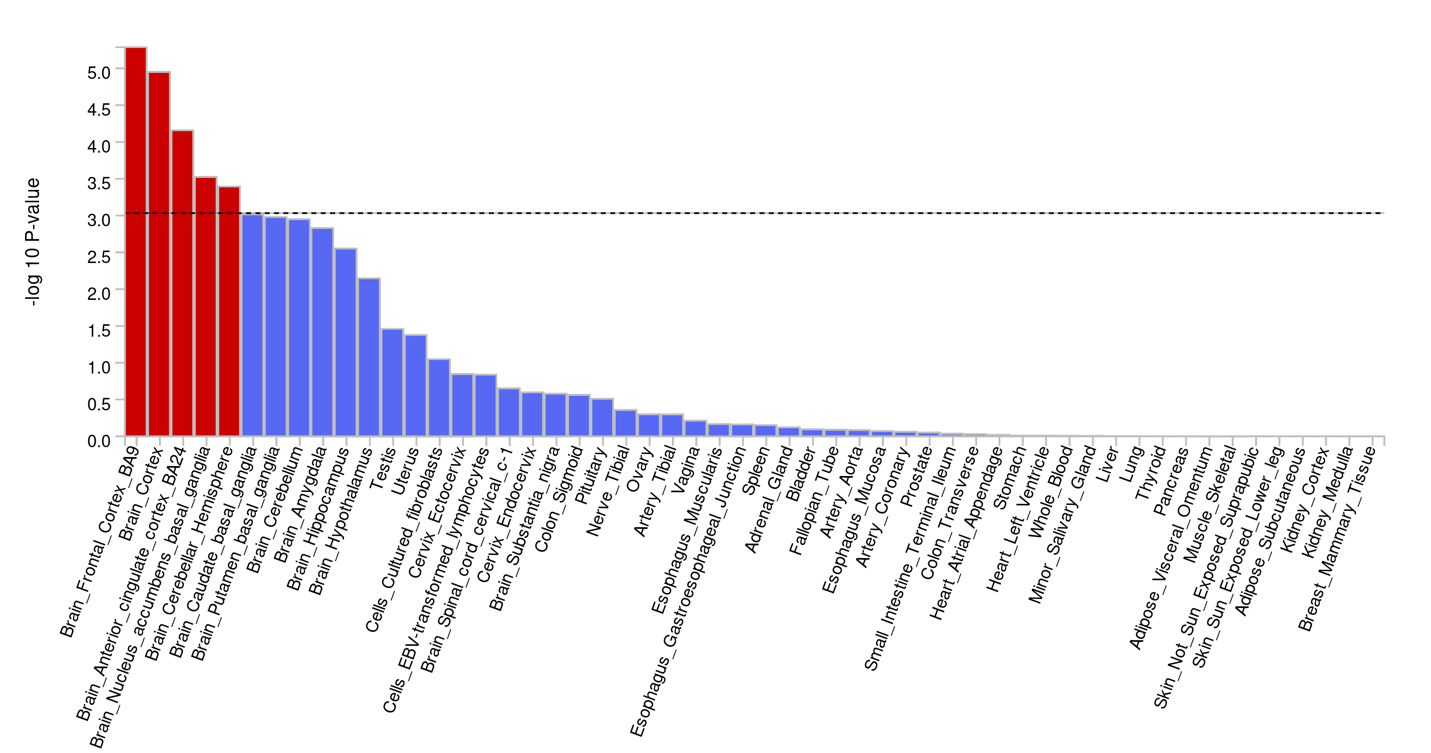** |

**Supplemental Figure 6.** Path model and standardized regression coefficients ( and standard error in parentheses) quantify the relationship between the caudate nucleus (CN) and migraine (MIG) while accounting for intracranial volume and the correlation between ICV and CN. When modeled together, 5% of the genetic variance in MIG is shared with CN and ICV, and Umig captures the unexplained variance in MIG (0.95, corresponding to 95%). The association between MIG and CN is positive after accounting for the negative relationship between MIG and ICV.


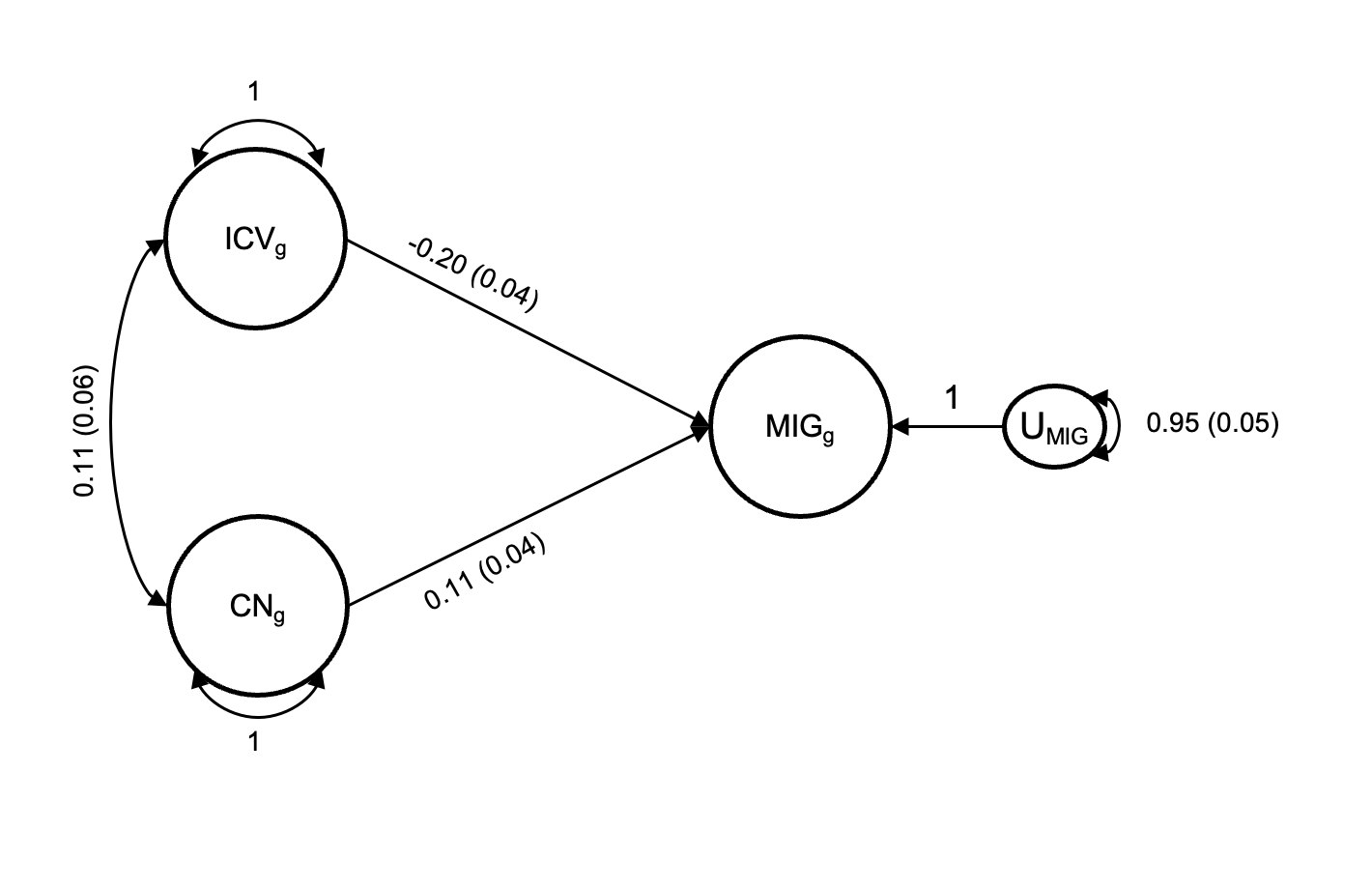


**Supplementary Figure 7.** Scree plot from parallel analysis of genome-wide association study summary statistics of MVP migraine in individuals of HARE-defined EUR ancestry and six psychiatric comorbidities (post-traumatic stress disorder, traumatic brain injury, major depressive disorder, anxiety, depression, attention-deficit hyperactivity disorder, problematic alcohol use) of EUR ancestry. Results indicate two eigenvalues above one, suggesting a two-factor solution to account for the relationship among these variables.


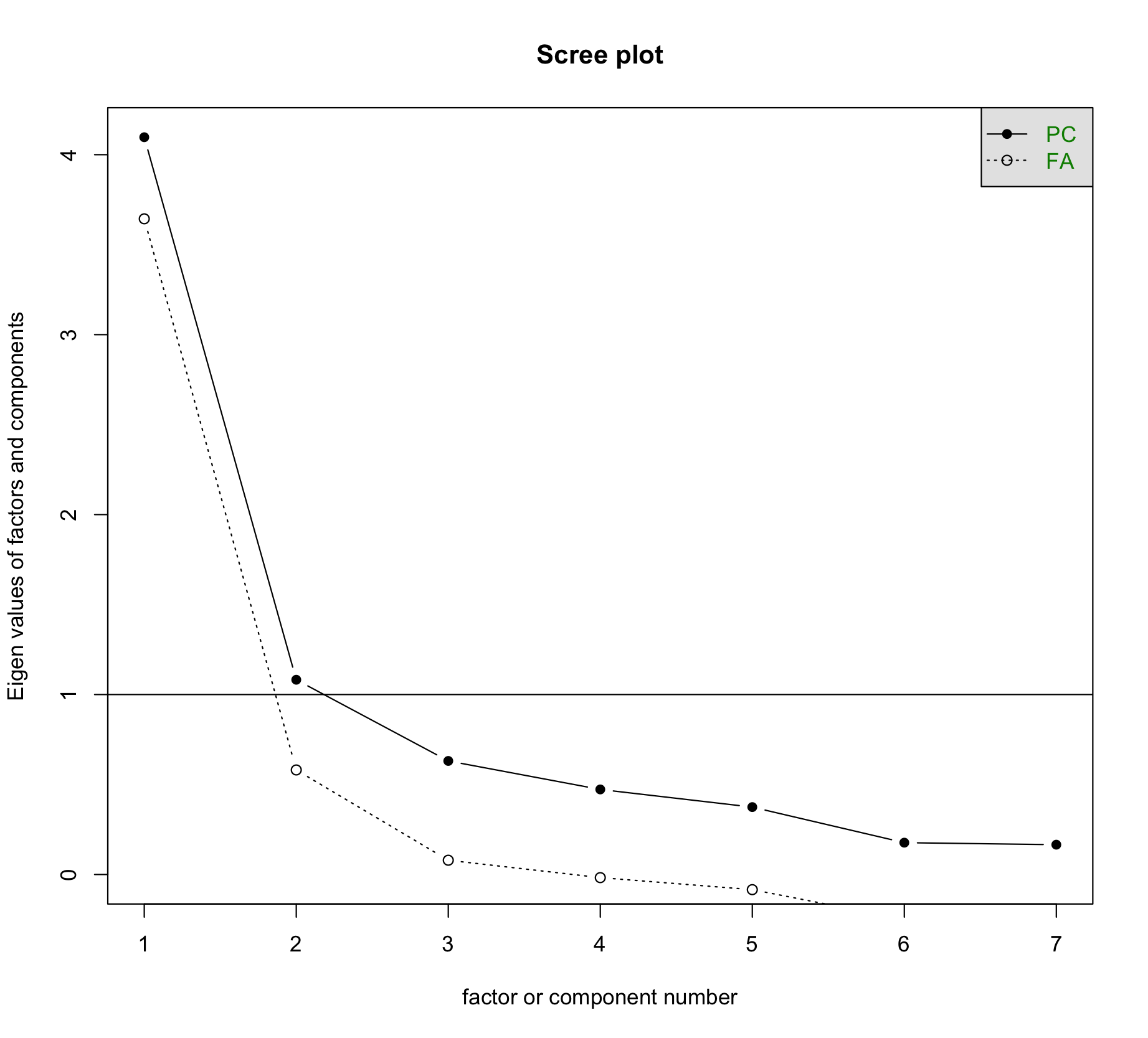


**Supplementary Figure 8.** Bivariate causal mixture model results. Presented are MiXeR analysis between MVP migraine META_Combined vs. GERA-UKB. **A)** The Venn diagram illustrates the number of causal variants (in thousands, with standard error) associated with MVP META_Combined migraine (indicated by the blue circle), GERA-UKB migraine (not displayed), and the shared variants represented in the grey overlap. Genetic correlation is a numerical value along with a red progress line bar. **B)** Conditional QQ plot of MVP migraine conditional on GERA-UKB migraine. **C)** Conditional QQ plot of GERA-UKB migraine conditional on MVP migraine. **D)** Model the log-likelihoods according to the number of causal variants. The solid blue line represents the average likelihood from the 20 MiXeR runs, while the dotted blue lines indicate the likelihoods for each individual run.

| **A.** | **B.** | **C.** | **D.** |
| --- | --- | --- | --- |


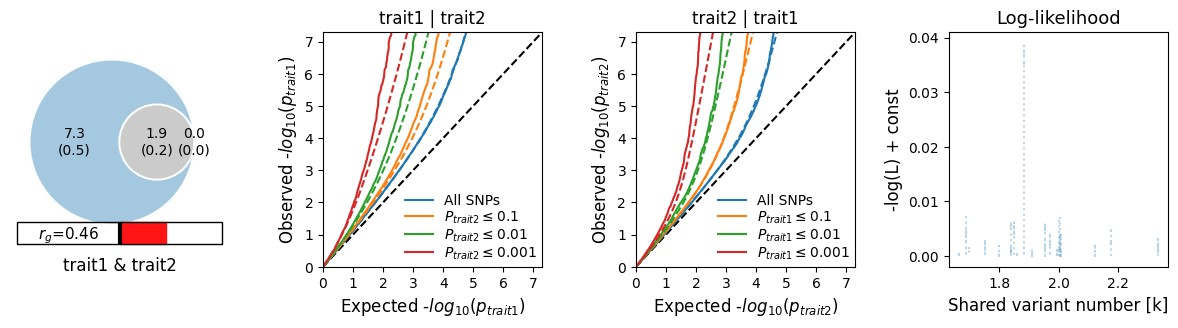


**Acknowledgments**

**VA Million Veteran Program**

**Core Acknowledgements for Publications**

**October 2025**

**MVP Program Office**

- Sumitra Muralidhar, Ph.D., Program Director

US Department of Veterans Affairs, 810 Vermont Avenue NW, Washington, DC 20420

- Jennifer Moser, Ph.D., Associate Director, Scientific Programs

US Department of Veterans Affairs, 810 Vermont Avenue NW, Washington, DC 20420

- Jennifer E. Deen, B.S., Associate Director, Cohort & Public Relations

US Department of Veterans Affairs, 810 Vermont Avenue NW, Washington, DC 20420

**MVP Steering Committee**

- Co-Chair: Philip S. Tsao, Ph.D.

VA Palo Alto Health Care System, 3801 Miranda Avenue, Palo Alto, CA 94304

- Co-Chair: Sumitra Muralidhar, Ph.D.

US Department of Veterans Affairs, 810 Vermont Avenue NW, Washington, DC 20420

- J. Michael Gaziano, M.D., M.P.H.

VA Boston Healthcare System, 150 S. Huntington Avenue, Boston, MA 02130

- Adriana Hung, M.D., M.P.H.,

VA Tennessee Valley Healthcare System, 1310 24th Avenue, South Nashville, TN 37212

- Dave Oslin, M.D.

Philadelphia VA Medical Center, 3900 Woodland Avenue, Philadelphia, PA 19104

- Deepak Voora, M.D.

Durham VA Medical Center, 508 Fulton Street, Durham, NC 27705

**MVP Co-Principal Investigators**

- J. Michael Gaziano, M.D., M.P.H.

VA Boston Healthcare System, 150 S. Huntington Avenue, Boston, MA 02130

- Philip S. Tsao, Ph.D.

VA Palo Alto Health Care System, 3801 Miranda Avenue, Palo Alto, CA 94304

**MVP Core Operations**

- Jessica V. Brewer, M.P.H., Director, MVP Cohort Operations

VA Boston Healthcare System, 150 S. Huntington Avenue, Boston, MA 02130

- Mary T. Brophy M.D., M.P.H., Director, VA Central Biorepository

VA Boston Healthcare System, 150 S. Huntington Avenue, Boston, MA 02130

- Kelly Cho, M.P.H, Ph.D., Director, MVP Phenomics

VA Boston Healthcare System, 150 S. Huntington Avenue, Boston, MA 02130

- Lori Churby, B.S., Director, MVP Regulatory Affairs

VA Palo Alto Health Care System, 3801 Miranda Avenue, Palo Alto, CA 94304

- Jacob T. Kean, Ph.D., Acting Director, VA Informatics and Computing Infrastructure (VINCI)

VA Salt Lake City Health Care System, 500 Foothill Drive, Salt Lake City, UT 84148

- Saiju Pyarajan Ph.D., Director, Data and Computational Sciences

VA Boston Healthcare System, 150 S. Huntington Avenue, Boston, MA 02130

- Robert Ringer, Pharm.D., Director, VA Albuquerque Central Biorepository

New Mexico VA Health Care System, 1501 San Pedro Drive SE, Albuquerque, NM 87108

- Luis E. Selva, Ph.D., Director, MVP Biorepository Coordination

VA Boston Healthcare System, 150 S. Huntington Avenue, Boston, MA 02130

- Shahpoor (Alex) Shayan, M.S., Director, MVP PRE Informatics

VA Boston Healthcare System, 150 S. Huntington Avenue, Boston, MA 02130

- Brady Stephens, M.S., Principal Investigator, MVP Information Center

Canandaigua VA Medical Center, 400 Fort Hill Avenue, Canandaigua, NY 14424

- Stacey B. Whitbourne, Ph.D., Director, MVP Cohort Development and Management

VA Boston Healthcare System, 150 S. Huntington Avenue, Boston, MA 02130
